# Biomechanics-guided computational design of virtual terrestrial vertebrate morphogenesis

**DOI:** 10.1016/j.isci.2026.117876

**Published:** 2026-10-15

**Authors:** Hui-Kai Zhang, Xi-Qiao Feng, Xing Xu

**Affiliations:** 1 Institute of Biomechanics and Medical Engineering, AML, Department of Engineering Mechanics, Tsinghua University, Beijing 100084, China; 2 Mechano-X Institute, Tsinghua University, Beijing 100084, China; 3 College of Mechanical and Electrical Engineering, Beijing University of Chemical Technology, Beijing 100029, China; 4 Key Laboratory of Vertebrate Evolution and Human Origins, Institute of Vertebrate Paleontology and Paleoanthropology, Chinese Academy of Sciences, Beijing 100044, China

**Keywords:** virtual vertebrate, computational morphogenesis, digital embryos, spatiotemporal topology assembly, biomechanics

## Abstract

To date, experimental development biology still faces challenges to efficiently model the morphogenesis of cross-species vertebrates, spanning from the embryonic development to postnatal growth. No effective computational methods are yet available to reproduce this complex and long-term process. However, recent advancements in large-scale computation and multi-voxel assembly theory have created unprecedented opportunities for establishing a virtual platform to simulate these morphomechanical processes. In this context, we propose a spatiotemporal topological evolution (STTE) method that can investigate the morphogenesis and postnatal growth of three-dimensional multi-voxel virtual embryoids. In this model, the optimization goal is to achieve extensive motion capabilities for the heads of virtual organisms in the vertical direction, thereby facilitating the formation and maintenance of conserved morphological structures characteristic of vertebrates. This proposed theory can rapidly and massively generate digital vertebrates with diverse adult body shapes, and this virtual morphogenesis pipeline offers novel perspectives and technologies for developmental biology.

## Highlights

• A biomechanical function-guided principle is proposed for terrestrial vertebrate morphogenesis
• A 3D spatiotemporal topological assembly is established to simulate allometric growth effects
• Massively diverse morphologies of virtual vertebrates are provided for examples

## Graphical abstract

**Figure:**
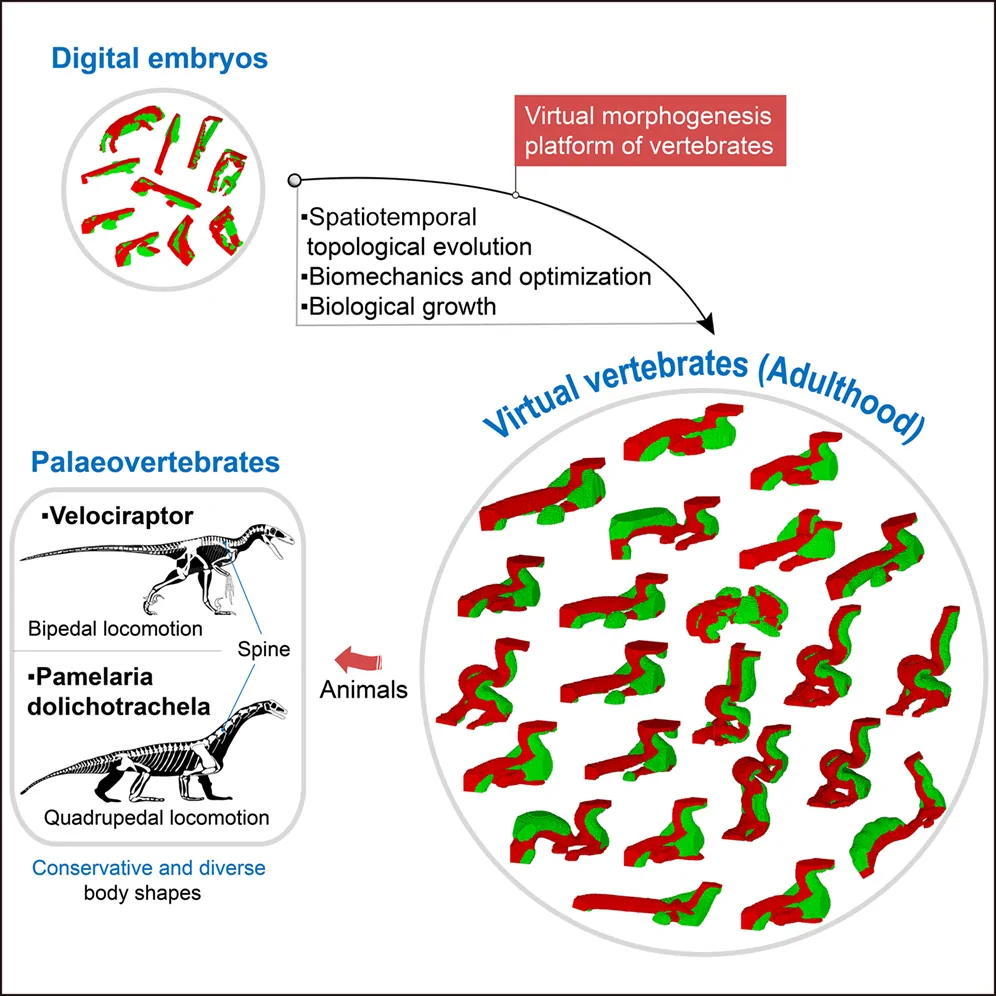

## Teaser

Biological sciences; Developmental biology; Biophysics; Biomechanics

## Introduction

Virtual digital life design is an essential component of the 21st-century biological science, and it remains a challenge to design vertebrates from embryonic to adult stages (Figure 1A). Recent groundbreaking advancements in artificial intelligence (AI) technology has revolutionized various fields, including DNA molecular switches,^1^ protein structures,^2^^,^^3^^,^^4^ virtual cells,^5^^,^^6^ and virtual animal development,^7^^,^^8^ indicating a potential shift in further life sciences research methods from classical experimental models to virtual digital models.^9^ The development of large–scale computational optimization methods^10^ and multi-voxel topological assembly theories^11^ will be instrumental in establishing a digital virtual platform for vertebrate development, facilitating deeper understanding of the lengthy processes of morphogenesis and species diversity, and advancing life design (Figure 1A). However, it remains unclear how biomechanical functions regulate the vertebrate morphogenesis.

**Figure 1:**
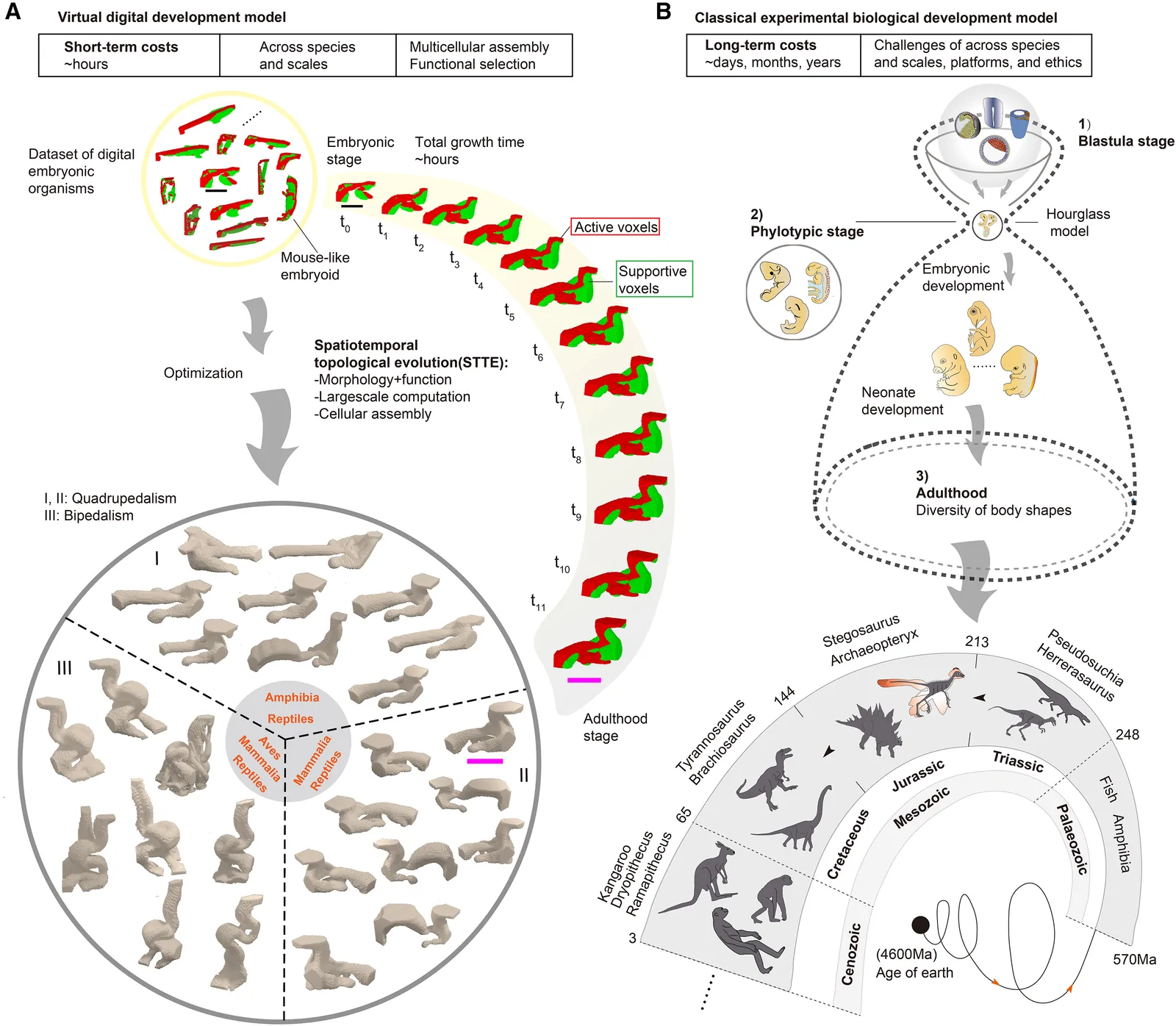
Classical experiment and virtual digital development model (A) A dataset of digital embryonic organisms is generated using the biomechanical function-guided multi-voxel inverse optimization method. In this model, red voxel can perform active contraction, simulating the muscle tissue in vertebrates. Subsequently, by employing the large-scale spatiotemporal topological evolution optimization method, we can rapidly obtain virtual cross-species adult vertebrates within a few hours. (B) The classical hourglass model of vertebrate development comprises three primary stages: (1) blastula formation: during this initial phase, collective embryonic cells form a neural tube that develops to the antero-posterior (A-P) body axis; (2) phylotypic stage: ss development advances, vertebrate embryos manifest distinct features such as heads, spines, and limbs; (3) late embryonic period: as embryos transition into newborns and beyond, the disparities in body shapes among different vertebrate populations become increasingly pronounced. Vertebrates exhibit a remarkable range of physical diversity through the process of natural selection. However, the biomechanical optimization principles that underline this highly conserved yet diverse developmental framework remains elusive, significantly impeding the establishment of the theoretical model for the virtual digital development platform. Note: 1Ma (million years ago) = 1,000,000 years.

Experimental embryology models (Figure 1B) have traditionally been the primary approach for investigating vertebrate body plans.^12^^,^^13^^,^^14^ To fully comprehend the vertebrate embryonic development, it is essential to track the fates of cell behaviors and the distribution of biochemical signals over long developmental periods and across large spatial scales. In murine development models, the organogenesis typically occurs between embryonic days E9.5-E14.5,^15^^,^^16^ while maturity can take nearly 50 days post-birth. Although *in vitro* embryonic development engineering has yielded insights into the mechanisms governing early multicellular development in vertebrates, current technologies permit mouse embryos to develop *in vitro* only up to approximately E11,^17^ at which point they have exhibited clear anterior-posterior (A-P) body axis characteristics. Additionally, due to ethical constraints and the scarcity of human embryonic samples, human *in vitro* embryonic development^18^ has reached a maximum of E25. Consequently, traditional embryological models and *in vitro* techniques face limitations in observing body shape formation across species and over lengthy periods, particularly concerning the development of paleovertebrates (Figure 1B) and some undiscovered vertebrates.

Modeling vertebrate embryonic development over extended periods through computer simulations necessitates considerations of deep physical optimization mechanisms that link genomics, cell movements, topological assemblies,^19^ and body plan (such as symmetry breaking and body axis formation).^20^ Studies indicated that the Hox gene family^12^^,^^21^ and BMP regulatory mechanisms^22^ play significant roles in forming the conserved A-P and dorsal-ventral (D-V) body axes in vertebrates, respectively. Moreover, the pronounced structural differences observed in mature individuals across vertebrate lineages highlight that embryonic cell behaviors and growth ultimately shape the diverse forms of vertebrates.^14^^,^^22^ However, it remains unclear how one can effectively optimize both the conserved and diverse phenomena of vertebrate development within a biomechanical function-guided strategy, significantly hindering the establishment of digital embryonic development models (Figure 1A).

To address these challenging issues, we propose a multi-voxel spatiotemporal topological evolution (STTE) method driven by biomechanical functions. The core principle involves an inverse search for the optimal topological configuration of multi-voxel systems that meets the defined functional objectives. This method can generate multi-voxel vertebrate-like digital embryoids of varied morphologies, which can further develop into mature virtual vertebrates based on a growth theory. The success of this strategy relies on the following three key components. First, we introduce voxels with active contraction capabilities (red) to simulate muscle tissue (Figure 1A), while green voxels represent supportive tissues such as epithelial cells or bone cells. Second, the proposed biomechanical framework defines head actuation as optimization objective and characterizes the standing behavior of the limbs, ensuring the random distribution of collective voxels to generate vertebrate-like body morphologies. Third, the growth process of digital embryoids is simulated by increasing the number of voxels, which is similar to the coarse-grain^23^ and cellular Potts model,^24^ diverging from the traditional volume growth theory.^25^^,^^26^ This approach offers significant advantages in three-dimensional (3D) topological evolution and large-scale growth simulations.

Using this theory, we have temporarily set aside the interactions between genes and cells to more effectively capture the critical factors involved in vertebral column formation. Future research will prioritize tackling these challenges and attempt to apply them to simulations of mouse embryonic developments.

## Results

### Model of STTE

By examining the body characteristics and common functions of various vertebrates (Figure S1), we define *γ* = *l*_2_/*l*_1_ as the body characteristic parameter that approximately describes the body axis of vertebrates during growth, where *l*_2_ = *l*_*y*_ and *l*_1_ = $\sqrt{2}l_{x}$ represent the body height and length of vertebrate animals, respectively (Figures 2A and S1), where *l*_*x*_ is the edge length of the design region along the *x*-direction (Figure S3). Notably, the embryonic body characteristic parameter at *t*_0_ is defined as *γ*_E_ = *γ*. Moreover, the head mobility shared by all terrestrial vertebrates is selected as the optimization objective (Figure S2). To obtain the vertebrate-like digital animals for both embryonic and mature stage, the primary red voxels and green supportive voxels is adapted to assembly the optimal morphology through the combination of topological variables **x**_1_ and **x**_2_ (Figures S3B and S3C) with the initial random values in [0, 1], which can converge to 0 or 1 by the proposed inverse optimization strategy in a design space (Figure S3A). In further studies, the initial values of **x**_1_ and **x**_2_ can be determined by using the spatial omics information^27^ of related embryoids.

**Figure 2:**
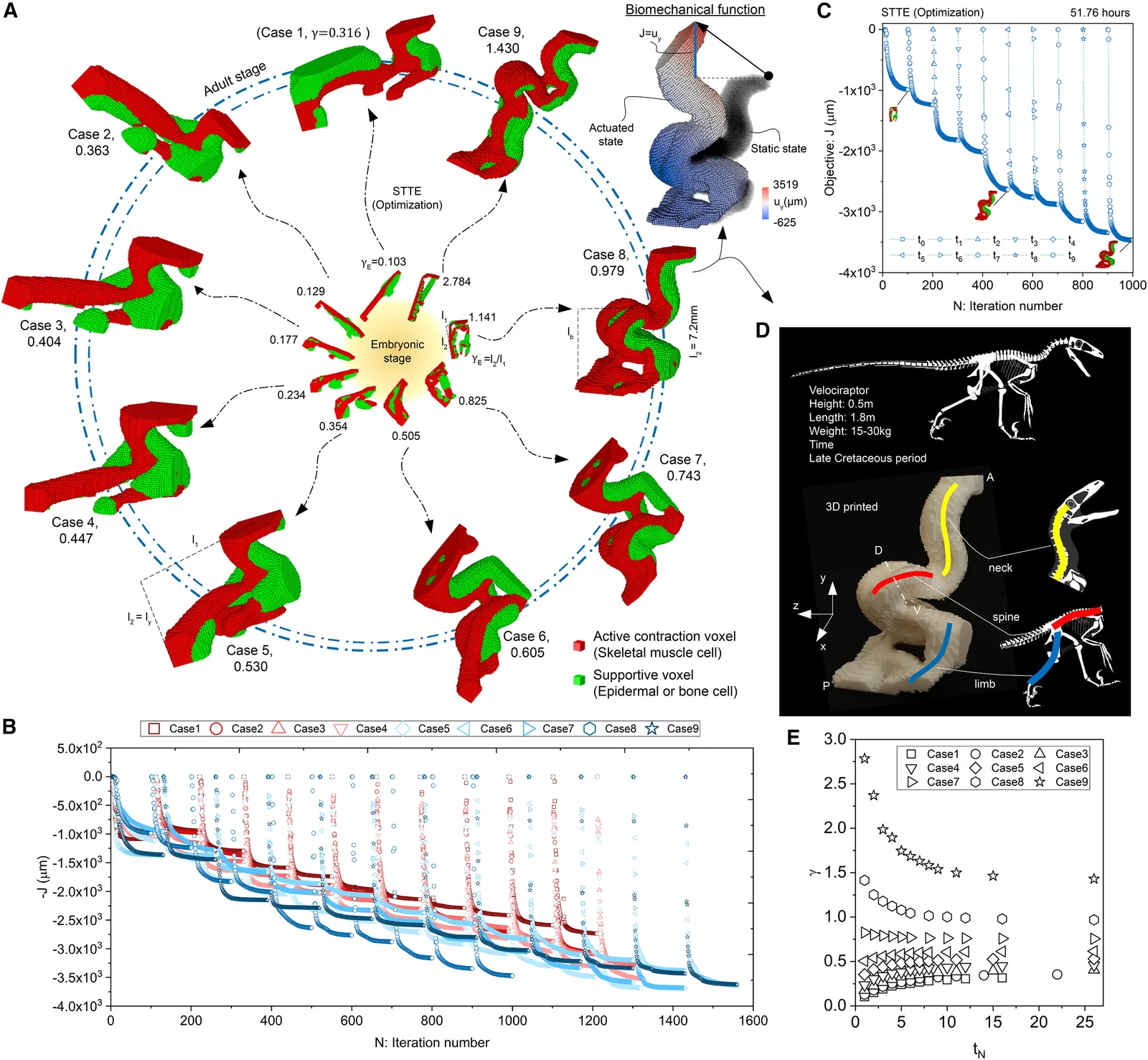
Spatiotemporal topology evolution of virtual vertebrates from embryonic to adult stage (A) Various optimized multi-voxel embryoids composed of active contraction (red) and supportive voxels (green) gradually develop into distinct virtual vertebrates; the sizes of embryonic and adult digital organisms in case 1 are *l*_*y*_ = 0.7 mm, $l_{1}=\sqrt{2}l_{x}$, *l*_*x*_ = *l*_*z*_ = 4.8 mm, and *l*_*y*_ = 3.4 mm, *l*_*x*_ = *l*_*z*_ = 7.6 mm, respectively. (B) Optimization histories for all cases. (C) The spatiotemporal evolution process of case8. (D) Comparison of the morphology of Velociraptor (graph is redrawn based on https://reptileevolution.com/velociraptor.htm) to the 3D-printed optimized vertebrate (case 8). (E) Changes in body parameters *γ* over growth time for all cases.

For instance, during the phylotypic characteristic period^14^ (Figure 1), multi-voxel embryonic organisms with distinct body topologies, ranging from case 1 to 9 (Figures 2A and S4–S6), are composed of red voxels and supportive voxels (green). These digital embryoids are the starting development point (t_0_) for their following growth and can be automatically generated using the proposed topological assembly method with initial random spatial distributions of voxels, with optimization objective $J=\max:u_{h\_y}$ (e.g., Equation 25) indicating that the heads exhibit maximum actuation ability along the *y*-direction (Figures S4 and S5). The red voxels assemble to an active tissue that mainly distributed on the dorsal position, which has the similar structure and function to the muscles in mouse embryo.^28^

The motion of the head drives the entire A-P body axis, which in turn facilitates the activity of the muscles in the limbs. Therefore, maximizing the locomotion capacity of the head aims to optimize distribution of contractile red voxels throughout the body, simultaneously promoting the formation of the head, neck, spine, and limbs. With an identical number of red and green voxels, they automatically assemble into various vertebrate-like embryonic structures characterized by diverse topological morphologies. These optimized virtual embryoids approximate the features of heads, limb buds, spines (Figure 2A), while exhibiting limited biomechanical actuation abilities (Figure S6).

In the STTE method (Figure S7), the growth process is contingent on the topological configurations and biological functions established during previous growth stages (Figure 2B). Studies indicate that during individual growth, the mass or volume must satisfy specific conditions, such as *dm*/*dt*→0 (see STAR Methods),^29^^,^^30^ where *m* represents the mass of the organism. For example, in case 8 (Figures 2A and 2C), the multi-voxel organism at embryonic stage comprises *n*_*x*_×*n*_*y*_×*n*_*z*_=(20×40×20)×20% voxels (Figures S8 and S9), with a corresponding body shape characteristic parameter of *γ*_E_ = 1.141. Under the same growth rate (*g*_*x*_ = *g*_*y*_ = *g*_*z*_) in the *x*, *y*, and *z*-directions, the optimization results at different growth moments are illustrated in Figure S9. In the early growth phase, significant changes occur in topological morphologies of multi-voxel structures, which gradually stabilize the growth progress. Specially, the optimized head size *A*_h_/*A*_0_, where *A*_0_ represents the area of the design space in the *x*-*z* plane, converges over time along with back height *l*_b_ and bottom limb area (Figure S10). The mature virtual organism in case 8 (*γ* = 0.979) shown in Figures 2A and 2D is composed of *n*_*x*_×*n*_*y*_×*n*_*z*_=(42×72×42)×20% voxels (the maximum number of voxels is dependent on the computational power of the computer, see STAR Methods) and has a height of 7.2 mm in the *y*-direction. It demonstrates well-defined vertebrate-like A-P and D-V body axes. The red contractile voxels predominantly localize at the vertebral positions on the back (Figure 2A). This enables them to fully exploit their active contraction capabilities, thereby enhancing the biomechanical propulsion abilities of the virtual vertebrate (Figure 2G). Along the A-P axis, voxels automatically assemble into key structures, including the head and spine, where the central nervous system is situated. Furthermore, the optimized virtual animal closely resembles the body characteristics of theropod dinosaurs, such as *Velociraptor* (Figure 2D), as well as modern vertebrates such as ostriches and swans (Figure S2). The optimized structure touching the design-space floor simulates the robust hind limbs of bipedal terrestrial animals. Despite limb morphological differences from real bipeds, it offers stable effective biomechanical function and body support.

In Figure 2A, we further investigate the topological morphologies of various virtual embryonic animals under isotropic growth conditions (Figures S11–S13). The mature virtual vertebrates across cases 1–9 (Figure 2A) exhibit an identical number of voxels, with body shape characteristic parameter *γ* progressively increasing (Figure 2C). In cases with smaller *γ*_E_ (Figure 2A), the mature vertebrates display body shapes akin to quadrupedal terrestrial reptiles (Figures 2A and S1; Table S1), characterized by a body length (along A-P axis) that significantly surpasses height, accompanied by short necks. In cases 1–4, the limbs of the optimized animals are far from the A-P body axis, resembling reptiles such as crocodiles or lizards. As *γ* increases, the angle between the A-P axis and the *x-z* plane also increases, leading to significant changes in the topological distribution of both types of voxels (Figure S11). In case 5, the limbs of optimized vertebrates are closer to the body axis, reflecting features of quadrupedal mammalian species (Figure S2). From cases 6 to 7, the forelimbs of the mature vertebrates progressively shit posteriorly, resulting in the formation of robust hind limbs that converge toward the body axis (Figure S14). Such changes in limb topology influence the locomotion patterns of mature vertebrates, marking the transition from quadrupedal to bipedal walking. For instance, ornithischians exhibit a quadrilateral belt,^31^ while theropods display a trilateral belt that enhances hind limb strength and load-bearing capacity.^32^^,^^33^

Moreover, the optimized results from case 9 (Figure 2A) reveal a body shape characteristic parameter of *γ* = 1.42, indicating an A-P axis nearing the vertical orientation, closely resembling the vertebral features of modern vertebrates, such as penguins—*Petradyptes stonehousei* (Figure S15). These findings highlight the significance of embryonic body characteristics (post-phylotypic stage) in shaping the diversity vertebrate species.

### Diversity of vertebrate body morphologies

Gene expressions at various temporal and spatial scales play a crucial role in regulating biochemical pathways that facilitates diverse organ development in vertebrates.^12^^,^^34^^,^^35^^,^^36^^,^^37^ For instance, ground finches exhibit earlier and higher BMP-4 expression, resulting in longer beaks compared to cactus finches.^38^ Fossil studies of Qianlong (a herbivore sauropodomorph dinosaur from Guizhou, China) show accelerated development of juvenile hind limbs,^39^ offering valuable insights into *sauropodomorph* evolution. However, conducting gene and fossil studies require substantial resources, and developing effective methodologies remains a challenge.

In response to these challenges, Figure 3 illustrates the effects of allometry on the model of spatiotemporal topology evolution. To elucidate the allometric pattern of vertebrates, multi-voxel digital embryoids exhibit variable growth rates along *x*, *y*, and *z*-directions, denoted as *g*_*x*_, *g*_*y*_, and *g*_*z*_. To achieve symmetrical multi-voxel digital vertebrates along the A-P axis, we stipulate that the growth rates in the *x*-*z* plane satisfy the condition of *g*_*x*_ = *g*_*z*_. Here, *g*_*y*_/*g*_*x*_ > 1 represents that the vertical growth rate exceeds that of either length or width; conversely, the growth rate in the *x*-*z* plane surpasses that in the vertical direction.

**Figure 3:**
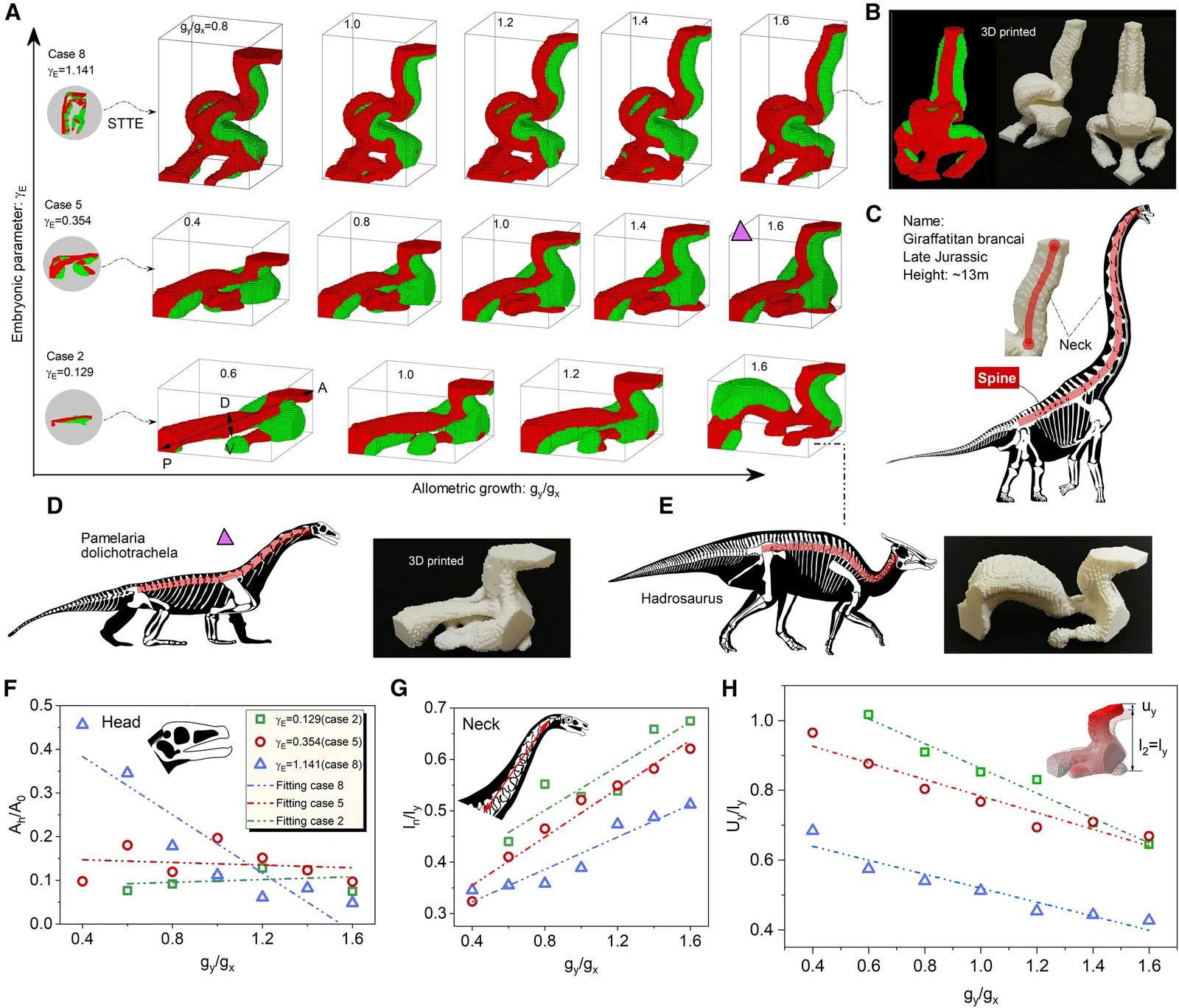
The impact of allometric growth on the development of virtual multi-voxel vertebrates (A) Three types of multi-voxel embryoids with different values of *γ*_E_ = 1.141(first line), *γ*_E_ = 0.354 (second line), and *γ*_E_ = 0.129 (third line) are taken to study the effects of allometric growth on mature digital vertebrate morphologies. (B) The final optimized vertebrate (g_*y*_/*g*_*x*_ = 1.6) is shown alongside its 3D-printed model. (C) *Giraffatitan brancai*^40^ (graph is redrawn based on Paul’s work^41^). (D) A comparison of *Pamelaria dolichotrachela*^42^ (graph is redrawn based on https://reptileevolution.com/pamelaria.htm) with its 3D-printed model is presented. (E) *Hadrosaurs* (graph is redrawn based on Słowiak et al.,^43^ an open access) is compared to the 3D-printed model. (F and G) Head and neck sizes of the optimized digital vertebrates vary according to their growth rates is shown. (H) The relationship between head mobility and allometric growth is illustrated.

Figure 3A presents three types of multi-voxel digital embryoids, including cases 2 (*γ*_E_ = 0.129), 5 (*γ*_E_ = 0.354), and 8 (*γ*_E_ = 1.141, as depicted in Figure 2A; Table S3 in supplemental information), each characterized by distinct body characteristics. Once the allometric growth rates are established, voxel increments in different directions can be computed according to Equation 3. The first line of Figure 3A illustrates the relationship between voxel increments and growth rates, as detailed in Figure S19. As *g*_*y*_/*g*_*x*_ increase, voxel number increments in the *y*-direction exceed those in the *x*-*z* plane. Additionally, Figure 3A displays all optimized outcomes at the mature stage (with all optimization processes are shown in Figures S16–18 and S20. Statistical analysis of these optimized vertebrates indicates that, as the growth rate *g*_*y*_/*g*_*x*_ increases, the size *A*_h_/*A*_0_ of the heads gradually decreases while the neck length *l*_n_/*l*_*y*_ increases (Figures 3F and 3G). All vertebrates depicted in Figure 3A possess an equal number of active contraction voxels, resulting in comparable magnitudes of active forces and head displacements *u*_*y*_ (Figure S20); however, the value of *u*_*y*_/*l*_*y*_ decreases with *g*_*y*_/*g*_*x*_ increasing (Figure 3H).

An increased growth rate *g*_*y*_/*g*_*x*_ in the *y*-direction facilitates anterior voxel migration, thereby enhancing embryo length.^14^^,^^22^ Excessive migration in this direction exhibits insufficient stiffness to withstand greater functional outputs in the spine and limbs, prompting an evolutionary strategy proposed by the intelligent STTE method to reduce the head size, thereby suppressing excessive voxel accumulation in the head region. This evolutionary strategy aligns with the anatomical body characteristics of quadrupedal *saurischia* and long-necked *sauropodomorph*, such as the approximate 10-meter-long neck and small head of the *Brachiosaurs*^35^^,^^44^^,^^45^ and *Giraffatitan brancai*^40^ from late Jurassic period (Figures 3B and 3C).

In the first line of Figure 3A, the embryonic organism has the body characteristic parameter of *γ*_E_ = 1.141, as *g*_*y*_/*g*_*x*_ increases (such as *g*_*y*_/*g*_*x*_=1.6), a pronounced bending feature of the neck emerges, accompanied by a larger angle of inclination relative to the horizontal plane (Figure 3B). Research reveals that *Brachiosaurs*, possessing thirteen cervical vertebrae,^31^^,^^46^ exhibit greater neck curvature and flexibility compared to modern giraffes. This anatomical adaptation enhances stability during locomotion, facilitating the evolutionary transition from bipedal (e.g., *Lufengosaurus*^47^ with short neck) to quadrupedal movement in large-bodied sauropods.^31^^,^^35^ Bipedal carnivorous theropod dinosaurs typically exhibit short, robust necks, and large heads, as exemplified by *Tyrannosaurus* rex (Figure 3F), which has a head length of 1.55 m,^48^^,^^49^ compared to *Brachiosaurs* head length of 0.8 m.

To support our theoretical findings, optimized topological morphologies of spines are all mapped onto the sample space of multiple vertebral skeletons from various animals (souring from https://reptileevolution.com/), as illustrated in Figure 4 (Figures S21 and S22; Table S4 in supplemental information). This mapping includes vertebrae from Aves (Figure 4A), long-necked sauropod dinosaurs (Figure 4B, souring from https://blogevolved.blogspot.com/2009/11/sauropod-gallery.html, and other animals (Figure 4C). For instance, the spine shapes of the optimized digital vertebrates (*g*_*y*_/*g*_*x*_ = 0.8, 1.0, case 8 in Figure 3A) are similar to the morphologies of real birds, such as the *Goura* and *Buteo buteo*. Additionally, we observe that the spine of *Daanosaurus* (Brachiosauridae) closely resembles the optimized morphology with *g*_*y*_/*g*_*x*_ = 1.6 (case 8, in Figure 3A).

**Figure 4:**
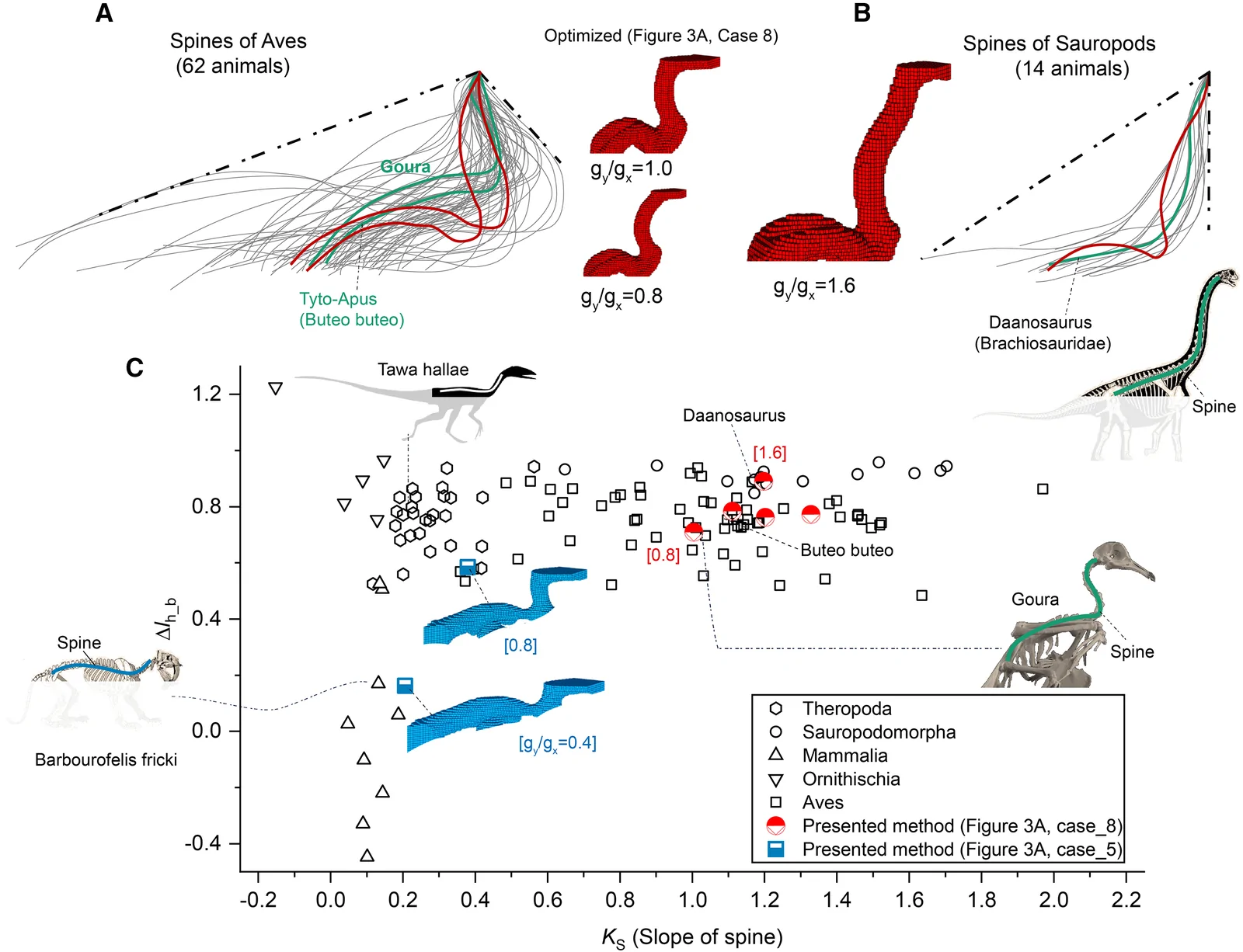
Vertebral morphology This figure illustrates the vertebral morphology of Aves (A), sauropod dinosaurs (B), and theropod dinosaurs (C). The vertebral data for Aves, theropods, sauropods, Mammalia, and Ornithischia are obtained from (https://reptileevolution.com/, Aves and dinosauria in vertebrata); and all animals are listed in Table S4 (supplementry information). The sauropod dinosaurs are sourced from (https://blogevolved.blogspot.com/2009/11/sauropod-gallery.html). *Daanosaurus* is sourced from (https://dinosaurpictures.org/Daanosaurus-pictures). All datasets are shown in Table S6.

Moreover, we define spine slope *K*_S_ and relative height from animal’s back to its head $\Delta l_{h\_b}$ (Figure S23) to perform comparisons between the optimized results and real spine morphologies. A larger *K*_S_ indicates a longer neck alongside the vertical direction of the animal, as seen in Aves and sauropods. In contrast, cases with smaller *K*_S_ typically correspond to animals with shorter necks (Figure 4C). When the spine slope *K*_S_ is specified (Figure 4C), such as cases in *K*_S_∈[0.1, 0.2] with a smaller $\Delta l_{h\_b}$, it suggested that the height of the animal’s back is closer to its head, often presenting a significantly curved spine in the dorsal region, as is evident in the comparison between *Mammalia* and *Theropoda* (Figure 4C). Our optimized long-neck vertebrates (indicated by red points) are positioned in the approximate regions with the $K_{S}-\Delta l_{h\_b}$ space, providing further support for the similar spine morphologies observed in Figures 4A and 4B.

In the second line (*γ*_E_ = 0.354), optimized animals display short, robust necks (Figure 4C, blue spines), with limbs positioned beneath their body (Figure 3A), mirroring the morphological traits of quadrupedal mammals (such as *Barbourofelis fricki*) or *ornithischian* dinosaurs^31^ (Figure 4C). As growth rate *g*_*y*_/*g*_*x*_ increases, such as *g*_*y*_/*g*_*x*_ = 1.6, significant neck enlargement in the digital vertebrate reflects the body shape of the *Pamelaria dolichotrachela*^42^ from the Triassic period (Figure 3D). In third line in Figure 3A (*γ*_E_ = 0.129), despite sharing an initial virtual embryonic configuration, asynchronous growth leads to notable changes in body shapes. The optimized vertebrate (Figure 3E) with *g*_*y*_/*g*_*x*_ = 1.6 is comparable to *hadrosaurs*.^43^

These findings indicate that the proposed function-driven principle is adopted by different vertebrate lineages, thereby enhancing the credibility of the proposed optimization model. Taken together, embryonic structural variation and allometric growth factors are essential in the evolution of terrestrial vertebrates. Figure 3A highlights the key characteristics of optimized quadrupedal and bipedal vertebrates, supported by comparative anatomical analyses of ancient and modern species (Figure 4), thus emphasizing the conservativeness of this evolutionary trajectory. In further, our virtual developmental model may present a valuable tool for exploring the critical transition from quadrupedal to bipedal locomotion, particularly in avian evolution^33^ (Figure S24), where the rotational adaptations of the ilium and femur are key anatomical changes.^32^ However, replicating this complex process in current experiments presents significant challenges.

### Predicting exotic vertebrates

Abnormal development of neural tubes and limbs is still relatively common in vertebrates, evident in cases such as conjoined twins^50^^,^^51^ (Figure S25) and six-legged mice.^37^ Shared chorion and amnion may lead to incomplete separation of embryos (Figure S23), resulting in bicephalic fetuses. Researchers can manipulate key protein networks to study vertebrate patterns^12^^,^^34^^,^^52^ and malformations^13^ highlighting the importance of understanding these mechanisms in embryonic development and regenerative medicine.

Our study focuses on bicephalic development in vertebrates, particularly the two-headed *Hyphalosaurus lingyuanensis* fossil from the Cretaceous period^53^ (Figure 5A). To create digital organism with two heads and necks during the embryonic stage, we set two independent output functions as optimization objectives, simulating head mobilities (Figure S26). In our optimization model, the objective function is redefined as $\max:\left(L_{1}^{T}+L_{2}^{T}\right)\;u$, where **L**_1_ and **L**_2_ represent the *i*- and *ii*-th head number vectors in the embryonic stage (Figure S26A). The design space for the bicephalic embryonic organism is initially set at 4 mm in height and 2 mm in width, with each type of voxel (red and green voxels) taking up 10% of the volume fraction. The development process is illustrated in Figure S26 along with a comparation to normal multi-voxel vertebrate-like embryoids (Figure 2A).

**Figure 5:**
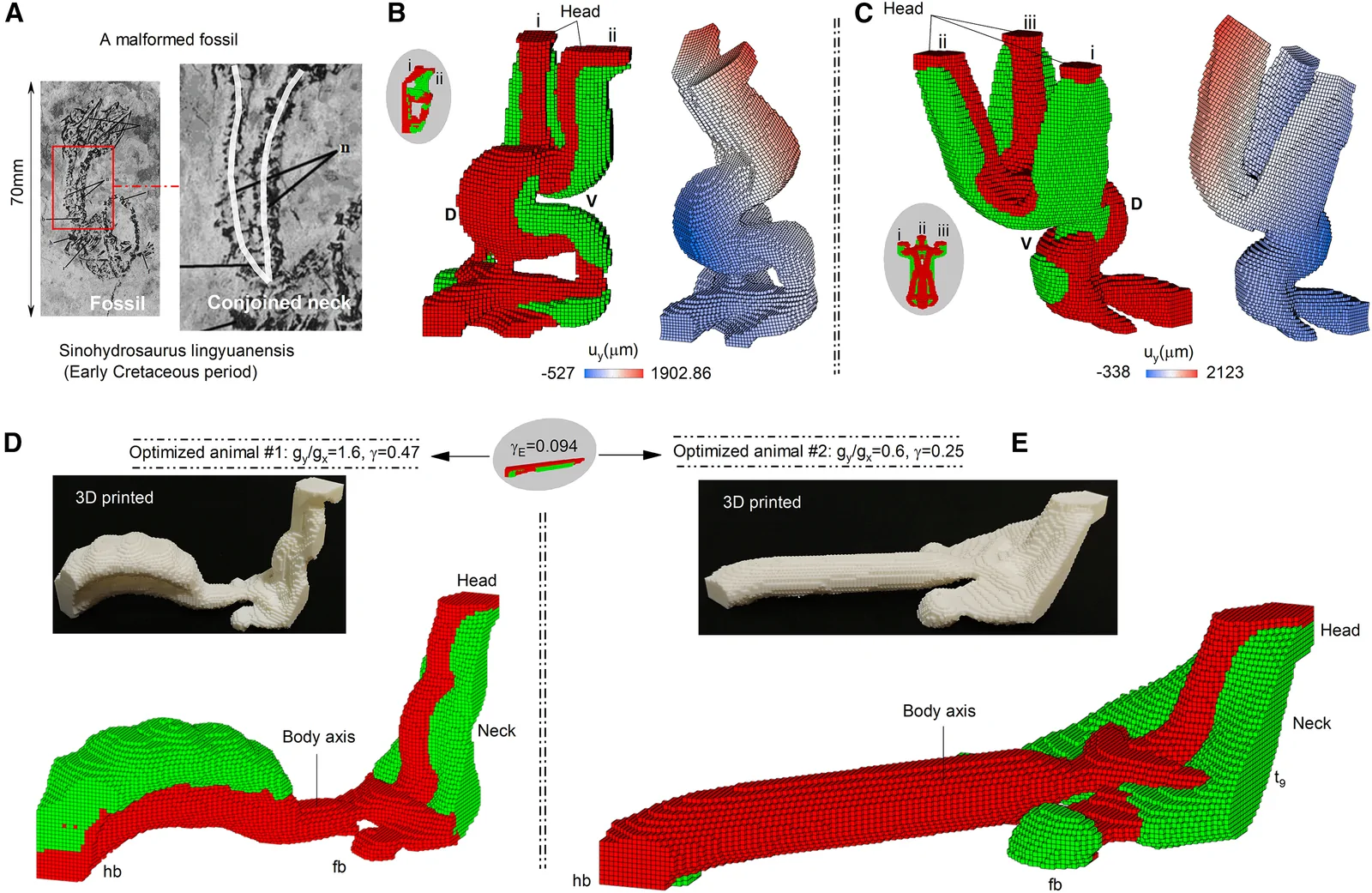
Predicting exotic vertebrates (A) Fossil of the bicephalic *hyphalosaurus lingyuanensis*^53^ (early Cretaceous period, reproduced with permission). (B) A mature multi-voxel vertebrate featuring two heads and necks, predicted by the spatiotemporal topology evolution method. (C) The optimized three-headed abnormal animal is presented. (D and E) Based on the embryonic organism with *γ*_E_ = 0.094, the adult optimized vertebrates exhibit a longer body axis with allometric growth *g*_*y*_/*g*_*x*_ = 0.6 in (E) compared to the case of *g*_*y*_/*g*_*x*_ = 1.6 in (D). The virtual vertebrate depicted in (E) closely resembles *Proteus anguinus*.^54^

The digital embryoid at *t*_0_ (Figure S26A) displays a marked second body axis (Figure 5B). As growth progresses, distinct structures corresponding to the two A-P axes in the malformed digital embryo develop and mature, ultimately resembling bicephalic *Hyphalosaurus lingyuanensis* in the adult from (Figure 5A). The model also predicts the characteristics of contemporary bicephalic *crocodiles* (Figure S27). To explore the universality of the STTE method, we present optimized virtual organisms with three body axes (Figures 5C and S28).

We examine the effects of spatiotemporal optimization when the characteristic parameter *γ*_E_ is significantly minimized, such as *γ*_E_ = 0.094, indicating that the body axis length substantially exceeds height. This scenario is assessed under two distinct growth modes with *g*_*y*_/*g*_*x*_ = 1.4 (Figures 5D and S29) and 0.6 (Figures 5C and S30), resulting in optimized vertebrates exhibiting a slender body shape (Figure 5E) and pronounced crawling limb structures. To support these theoretical findings, we reference two extant species known for their slender bodies and crawling capabilities, such as *Proteus anguinus*^54^ (Figure 5E) and the *Takydromus sauteri*,^55^ reinforcing the biomechanical optimization principles in such vertebrate existence.

### Virtual mouse-like vertebrate development

To investigate the application of our method in mimicking mouse development (Figure 6; Table S5), we established a computational domain based on computed tomography (CT) data of mouse morphology^56^ on the first day postnatally (Figure 6A) instead of the body shape at the phylotypic stage due to the limitation of the 3D dataset being unobtained. The design structure aims at optimizing the multi-voxel digital embryo morphology at growth time t_0_ (Figure 6B, the similar process in Figure S5), where the displacement boundary conditions and optimization objectives for the digital mouse’s limbs are illustrated in Figure 6B. The optimized active red tissues have the similar morphology (Figure S33) to the muscles during the early mouse embryo development.^28^ Although the optimized digital mouse has a tail at t_0_, we temporarily disregard this feature for simplification in subsequent analyses. Utilizing spatiotemporal evolution theory (Figure S7), Figures 6C and 6D present the digital vertebrates at different growth times from t_0_ to t_6_. The entire optimization history also demonstrates a stable convergence process (Figure 6E). Figure 6D depicts the resting and active states of the digital mouse-like animals at various growth time points (see Videos S1 and S2). As the red voxels undergo active contraction, significant changes are observed in the digital mouse’s limbs, trunk, and head, reflecting the multi-voxel morphology imparted by the optimization (Figure 6F) and its biomechanical driving functions. Figure 6G illustrates the stress distribution in the mouse during the driving process. The study indicates that regions with red voxel distribution exhibit higher stress, which is a result of the active contraction of red voxels during the driving of the digital mouse-like animals. According to this platform, we further consider early embryo development by taking cell behaviors^20^^,^^28^^,^^57^ into the theoretical model.

**Figure 6:**
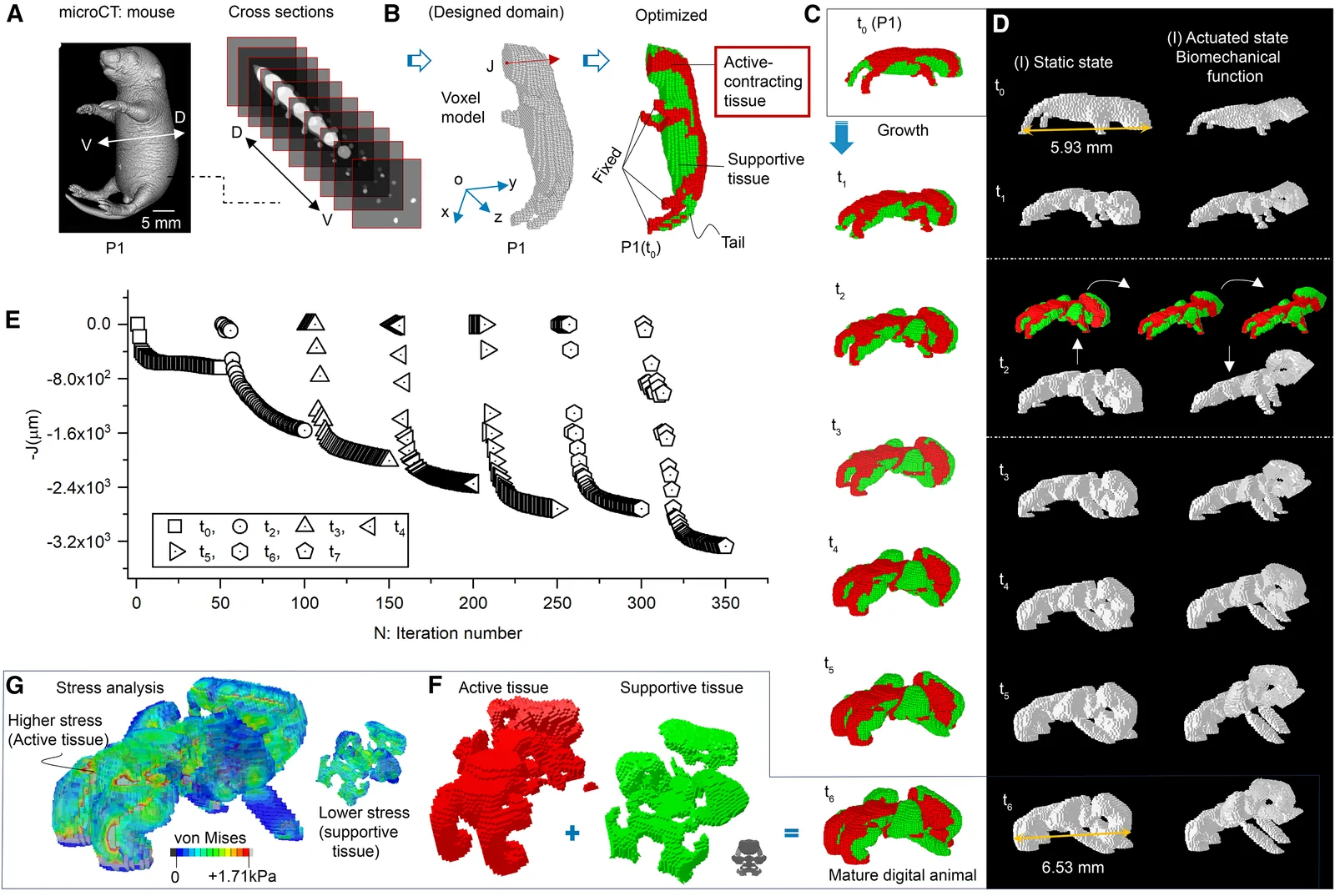
Virtual mouse-like animal (A) 3D model of early postnatal mouse and its cross sections samples from CT datasets^56^ (reproduced with permission). (B) Optimization domain (left) and the optimized digital animals (right, at t_0_). (C) All growth process of the virtual animal development. (D) Biomechanical functional simulations of the optimized animals during the growth processes. At the static stage (I), all active red voxels do not work during this stage, while they start to contract during the second actuated stage (II). (E) Optimization histories for the growing processes. (F) The morphologies of the optimized active and supportive tissues. (G) Stress analysis of the actuated mature vertebrate-like animal (t_6_).

Video S1. The topological structures of the optimized mouse-like vertebrate and its active actuation are presented, related to Figure 6D (*t*_2_)

Video S2. Displacement fields of the optimized mouse-like vertebrate during actuation, related to Figure 6D (*t*4)

## Discussions

The virtual computational platform proposed in this paper is grounded in the STTE theory, which serves as a foundational optimization model to mimic the adult morphology of virtual vertebrates based on any given digital embryonic organism. This model integrates multiple dimensions, including cell-like voxel activity, topological assembly, growth theory, and function-oriented optimization, thus enabling the simulation of virtual embryonic development over an extended period. At this stage, elucidating the physical principle underlying the conserved yet diverse body shapes of vertebrates represent a significant task, making a critical advancement in the of the virtual digital development platform.

Our approach constructs a multi-voxel system composing of active contracting voxels (e.g., skeletal muscle cells) and passive support voxels. We define the maximum vertical mobility of the head as the optimization objective for the digital embryonic organism, thereby ensuring that the multi-voxel organism at this developmental stage exhibits the morphological characteristics of vertebrates. Driven by spatiotemporal growth effects, the multi-voxel organism inherits the topologies and functions from the preceding moment, ultimately displaying significant vertebrate features such as the head, neck, spine, and limbs, along with stable driving functions upon reaching maturity. Moreover, the STTE theory introduces concepts such as phylogenetic characteristic periods, allometric growth, and malformation, which facilitate the prediction of the diverse morphologies observed in vertebrates.

We have printed virtual adult vertebrates (Figures 1A and S31) and found that their morphology aligns with various extant terrestrial vertebrates, such as bipedal birds, upright penguins, and quadrupedal mammals, thus fully reflecting the reliability of the STTE approach. Additionally, we investigate the impact of allometric growth on vertebrate morphology. Our research indicates that when the growth rate in height surpasses that in length, the size of the vertebrate’s neck increases significantly, while head size gradually decreases. This finding theoretically elucidates the functional selection processes, such as the adaptation for feeding leaves from tall trees, which influences the long neck development seen in sauropod dinosaurs. The model further predicts abnormal developmental phenomena, including two-headed vertebrates, a prediction supported by findings from the Early Cretaceous fossil of *Hyphalosaurus lingyuanensis*.^53^ We hope that this paper would attract much interest in the establishment of virtual embryonic development platforms and yield valuable insights across broader domains, including developmental biology, species discovery,^58^ bionic vertebrate-like robotics design,^59^^,^^60^ and biomedical research in general.

### Limitations of the study

The proposed model is based on the topology assembly theory of multi-voxel systems during embryonic. It is highlighted that this approach still has some limitations. The effects of the initial patterns of red and green voxels, the boundary conditions, and the initial geometrical shapes on the morphogenesis need further considerations. For example, the topological patterns in digital multi-voxel embryo (as shown in Figure 6) neglect the spatial genomic^61^^,^^62^^,^^63^ and bio-chemical information over time (influencing physical properties of voxels, Figure S32), hindering quantitative comparisons with cellular patterns observed in real developmental experiments. Looking ahead, our goal is to systematically integrate spatial omics^64^^,^^65^ and cellular interactions^66^ into the theoretical model to reveal further developmental mechanism.^28^^,^^67^ Additionally, due to computation constraints, the sizes of the optimized digital vertebrates are much smaller than those of real embryoids and mature vertebrates. As the number of voxels in digital embryos increases, the large-scale computational demands^10^ associated with the STTE method are expected to rise significantly. In this process, the scaling growth law across virtual animals (Figures S34–S36) requires further investigations.

## Resource availability

### Lead contact

Further information and requests for resources and reagents should be directed to and will be fulfilled by the lead contact, Xi-Qiao Feng (fengxq@tsinghua.edu.cn).

### Materials availability

This study did not generate any new, unique materials.

### Data and code availability

• This study did not generate new data.
• Original codes have been deposited and made publicly available (https://zenodo.org/records/18197885?token=eyJhbGciOiJIUzUxMiJ9.eyJpZCI6IjZiOWI2OTIyLTg4MmQtNDk2OC1iMDBhLTMzNWYwZDZjMTMyMyIsImRhdGEiOnt9LCJyYW5kb20iOiI1YzhhODA5MjcxZWQwODRiNjFkNjE0MTJlOTQyOWIxMiJ9.bIyrY6IZ_Wwz6n58mj3s41GAJxPEzSHsfzP7sTO1lhpP8B3EwEGs884URDyjkQqNgvCqsmgPqEV0HSNMASC_CA).
• Additional information required to reanalyze the data in this paper is available from the lead contact upon request.

## Acknowledgments

H.-K.Z. would like to express heartfelt gratitude to professor X.-Q.F. for invaluable support during the author’s free explorations over the past years. The first author is also deeply thankful to his parents, Mr. Zhang and Mrs. Sun, for their unwavering encouragement and support. This work is dedicated to the memory of his beloved grandmother. Additionally, the authors extend their sincere appreciation to Professor A.M. Meng, B. Li, and Associate Professor F. Zhou from Tsinghua University for their insightful discussions. H.-K.Z. is also grateful to Professor H.C. Zhao and PhD student, Q. Shao from Tsinghua University for their assistance with 3D printing of our optimized results. The authors acknowledge the support from the National Natural Science Foundation of China (grant no. T2488101) and the China Postdoctoral Innovation Talent Support Program (grant no. 043214012).

## Author contributions

H.-K.Z., conceived this project and performed the research, conceptualization, funding acquisition, methodology, project administration, supervision, and writing – review and editing; X.X., conceptualization, data analysis, and writing – review and editing; X.-Q.F., conceptualization, funding acquisition, project administration, resources, supervision, and writing – review and editing.

## Declaration of interests

The authors declare no competing interests.

## STAR★Methods

### Key resources table

**Table:** 

REAGENT or RESOURCE	SOURCE	IDENTIFIER
**Biological samples**		

Aves and dinosauria (Figure 4)	Reptile evolution	https://reptileevolution.com/
Sauropod dinosaurs (Figure 4)	The online palaeo-art community	https://blogevolved.blogspot.com/2009/11/sauropod-gallery.html
Daanosaurus (Figure 4)	Daanosaurus pictures and facts	https://dinosaurpictures.org/Daanosaurus-pictures
3D mouse model (Figure 6A)	Published paper	Hsu et al.^56^

**Software and algorithms**		

MATLAB R2021b	MathWorks	https://www.mathworks.com/
Abaqus 2018	Dassault Systems	https://www.3ds.com/products/simulia/abaqus
Spatiotemporal topology evolution (STTE)	Presented in this paper (Data and code availability)	https://zenodo.org/records/18197885?token=eyJhbGciOiJIUzUxMiJ9.eyJpZCI6IjZiOWI2OTIyLTg4MmQtNDk2OC1iMDBhLTMzNWYwZDZjMTMyMyIsImRhdGEiOnt9LCJyYW5kb20iOiI1YzhhODA5MjcxZWQwODRiNjFkNjE0MTJlOTQyOWIxMiJ9.bIyrY6IZ_Wwz6n58mj3s41GAJxPEzSHsfzP7sTO1lhpP8B3EwEGs884URDyjkQqNgvCqsmgPqEV0HSNMASC_CA

### Method details

#### Ontogenetic growth model

The organ growth can be described through the principles of continuum mechanics,^25^^,^^36^^,^^68^ vertex dynamics,^69^^,^^70^^,^^71^ particle system,^72^ and mathematical approaches^29^^,^^30^ methods. In continuum mechanics, the volumetric change of an organ is characterized by a deformation gradient tensor **F**=**F**_e_·**F**_g_, which integrates both elastic deformation **F**_e_ and volume growth components **F**_g_, respectively.^73^ Volumetric growth is influenced by various factors, including external nutrient supply^74^ and active stresses.^75^^,^^76^ These growth theories have been extensively applied to investigate morphological development in various organs, such as the brain,^77^^,^^78^^,^^79^ skin,^80^^,^^81^ esophagus,^82^ intestine,^83^ blood vessels,^84^^,^^85^^,^^86^ nerves,^87^ tumors,^88^^,^^89^^,^^90^ mollusk shells,^91^ organoids,^75^ and plant leaves.^68^ However, prior theoretical investigations have often neglected the intricate topological transformations that occur during the 3D growth process of organs. Recent advancements *in vitro* multicellular experiments have made it feasible to observe and manipulate the 2D or 3D topological evolution within multicellular organoids during self-organizing development phases.^92^^,^^93^ However, existing theoretical models typically focus on organ or cellular spheroid morphogenesis without adequately considering special biological or biomechanical functions.

To elucidate the topological evolution of ontogenetic development from the embryonic stage to the adulthood, this section introduces mathematical models that quantitatively describe growth. This model facilitates the comprehensive description of embryo mass and cell count at various growth intervals. Furthermore, it explains how the design space for the subsequent growth phase is updated based on predefined initial design parameters and growth rules (Figure S4).

The ontogenetic growth model, which incorporates the metabolic energy contained within the existing organism along with the production of new biomass,^29^ can be represented as follows:

(Equation 1) $$\frac{dm}{dt}=am^{3/4}-bm=am^{3/4}\left[1-{\left(\frac{m}{M_{\max}}\right)}^{1/4}\right],$$

where *M*_*max*_=(*a*/*b*)^4^ denotes the maximum mass that can be obtained when *dm*/*dt* = 0. $a=B_{0}m_{c}/{\tilde{E}}_{c}$ and $b={\tilde{E}}_{c}/B_{c}$, where *B*_c_, ${\tilde{E}}_{c}$, and *m*_c_ represent the metabolic rate of a single cell, the energy required for cell division, and the cellular mass, respectively. In this equation, the growth of an organism is predominantly governed by the number of cells rather than the volume of individual cells, and the corresponding parameters are *B*_0_ = 1.9 × 10^-2^ W, *m*_c_ = 3 × 10^-9^ g, and ${\tilde{E}}_{c}=2.1\times 10^{-5}J$, respectively.

To accurately capture the topological growth during ontogenesis, a voxel topology optimization method is employed to evolve the multicellular embryoid. The design domain is discretized into cubic voxels (Figure S4), each representing a 3D cell.^11^ According to the ontogenetic model, the total cell count within the growing body can be expressed as

(Equation 2) $$N\left(t\right)={\left\{1-\left[1-{\left(\frac{n_{0}}{N_{\max}}\right)}^{1/4}\right]e^{\left[\left(-\tilde{a}t/4\right)N_{\max}^{1/4}\right]}\right\}}^{4}N_{\max}f^{*}\text{,}$$

where *N*_*max*_ is the maximum total cell count within the design space, and *f*∗ refers to the living cell volume fraction within the design domain. Consequently, the final volume of the organism can be represented as *V*(*t*) = *N*(*t*)*V*_c_, and $V_{c}=l_{e}^{3}=0.1\times 0.1\times 0.1\;{\text{mm}}^{3}$ approximating the volume of a *Xenopus laevis* cell,^11^^,^^94^ where *l*_e_ is the edge length of the cubic voxel element. $\tilde{a}$ and *f*∗ are 0.2 days^−1^ and 20%, respectively. The size of the unit voxel is determined by the computational capacity of the computer. If the cubic voxel size *l*_e_ is reduced, for instance, to 20 μm, the height of the adult digital vertebrates will consequently decrease. In future studies focused on mouse embryonic development, a voxel size of 20 μm will be utilized, necessitating a more powerful computer equipped with 256 GB or 512 GB of RAM to ensure optimal performance (see scaling analysis in Figures S34–S36).

#### Spatiotemporal topology evolution theory

The topology optimization method aims to identify the optimal material distribution within a specified design space, guided by a particular objective.^10^^,^^95^ In nature, many structures exhibit diverse ranges of forms, particularly during processes like organ growth and embryo development. Consequently, it is essential to consider changing design boundaries throughout the optimization process, which may involve factors such as pressure dependence^96^^,^^97^^,^^98^ and the growth of plant roots.^99^ Additionally, topological transformations are frequently observed in organ evolution; however, the integrating topological evolution within the context of organ growth presents a complex challenge. To address this issue, we propose a topology optimization method that incorporates spatiotemporal memory effects to explore the evolution of ontogenesis.

##### Discrete growth strategy of the digital organisms

As shown in Figure S7 at a given growth time *t*_*k*-1_, the design domain exhibits a specific function that maximizes displacement output at a point, aimed at stimulating head motion in vertebrate structure. At the subsequent growth time *t*_*k*_, the multi-voxel organism must maintain a similar functional capacity while accommodating body growth and topology evolution, akin to the development changes observed in various organs such as the heart,^100^^,^^101^ the leaf of Monstera obliqua,^102^ the inner ear of zebrafish,^103^ and organoids.^63^^,^^93^^,^^104^ As a result, the optimized outcome at time *t*_*k*-1_ serves as a reference input for the red domain at time *t*_*k*_ (Figure S7).

In the voxel optimization model, the unit voxel increments along the edges of the design domain at time *t*_*k*_ are determined by the following equation:

(Equation 3) $$\begin{array}{l} \left[n_{x}\left(t_{k-1}\right)+\Delta n_{x}\right]\times \left[n_{y}\left(t_{k-1}\right)+\Delta n_{y}\right]\times \left[n_{z}\left(t_{k-1}\right)+\Delta n_{z}\right]=N\left(t_{k}\right)\to \\ \left[n_{x}\left(t_{k-1}\right)+g_{x}\Delta t\right]\times \left[n_{y}\left(t_{k-1}\right)+g_{y}\Delta t\right]\times \left[n_{z}\left(t_{k-1}\right)+g_{z}\Delta t\right]=N\left(t_{k}\right)\text{,} \end{array}$$

where *g*_*x*_, *g*_*y*_ and *g*_*z*_ represent the growth rates along the respective axes, and Δ*t* signifies the time increment. Additionally, Δ*n*_*x*_, Δ*n*_*y*_ and Δ*n*_*x*_ denote the increase in voxel counts along each growth direction, with the volume fraction suggested as *f*∗ = 20%. The edge voxels along the *x*, *y* and *z*-directions are defined as *n*_*x*_, *n*_*y*_ and *n*_*z*_, respectively.

##### Biomechanical model and finite element

The digital organism at *t*_0_ is composed of two types of voxels, as illustrated in Figure S3. The red has active contraction capacities, while the green voxels do not. We adopt a linear elastic constitutive and small strain (linear geometry) assumption to represent the active stresses generated by cardiomyocytes (red voxels), the active constitutive relation has the following form^11^

(Equation 4) $$\sigma_{\text{ela}}=D\left(\varepsilon -\varepsilon_{a}\right)=\sigma -\sigma_{a,}$$

in this equation **σ** = **Dε** is the total stress, **σ**_ela_ and **σ**_a_ = **Dε**_a_ denotes the elastic and active stresses, respectively, **D**(*v*,*E*_c_) is the elastic matrix with *v* and *E*_c_ being the Poisson’s ratio and elastic modulus of the red voxels, respectively. The Young’s modulus for the red active contraction voxel (representing the skeleton muscle cell) is denoted as *E*_c_ = 100 kPa^11^ (*Xenopus laevis*). It is assumed that each cell maintains a uniform Poisson’s ratio *ν* = 0.3.

**D**(*v*,*E*_c_) is written as

(Equation 5) $$D=\frac{E_{c}}{\left(1+v\right)\left(1-2v\right)}\left[\begin{array}{cccccc} 1-v & v & v & 0 & 0 & 0\\ v & 1-v & v & 0 & 0 & 0\\ v & v & 1-v & 0 & 0 & 0\\ 0 & 0 & 0 & \left(1-2v\right)/2 & 0 & 0\\ 0 & 0 & 0 & 0 & \left(1-2v\right)/2 & 0\\ 0 & 0 & 0 & 0 & 0 & \left(1-2v\right)/2 \end{array}\right]\text{,}$$

**ε** and **ε**_a_ are the total and the active strain, respectively. The active strain is written as

(Equation 6) $$\varepsilon_{a}=\Delta C{\left[\alpha_{x},\alpha_{y},\alpha_{z},0,0,0\right]}^{T}\text{,}$$

where Δ*C* is the nutrient concentration (e.g., ATP or Ca^2+^ ions), which can be adjusted. *α*_*x*_, *α*_*y*_, and *α*_*z*_ are the contraction coefficients of the red voxel in the *x*, *y*, and *z*-directions, respectively. In this study, we set *α*_*x*_ = *α*_*y*_ = *α*_*z*_*α* = −0.1. If the nutrient concentration Δ*C* is set to zero, the active strain ε_a_ will vanish. leading to the stress of passive supporting green voxels in Equation 4 reducing to **σ**_ela_ = **D**(*v*,*E*_e_)**ε** = **σ**, where *E*_e_ = 10 kPa is the elastic modulus of supporting voxels (representing the epidermal cells or bone cells). Based on the total potential energy function of the organism,^11^ we can derive the governing equation in a finite element form

(Equation 7) $$\text{Ku}=P+P_{\text{bio}-\text{chemo}}\to u=K^{-1}\left(P+P_{\text{bio}-\text{chemo}}\right)\text{,}$$

where **K**(*v*,*E*_c_,*E*_e_) and **u** represent the global stiffness matrix and displacement vector, respectively. The equation can be solved using the Preconditioned Conjugate Gradient method (PCG) in MATLAB. The global stiffness matrix is expressed as

(Equation 8) $$K\left(v,E_{c},E_{e}\right)=\sum_{i=1}^{N}k_{i}=\sum_{i=1}^{N}\int_{\Omega_{i}}B^{T}D\left(v,E_{c},E_{e}\right)Bd\Omega \text{,}$$

where **k**_*i*_ is the stiffness matrix of the *i*th element; The strain matrix is given by

(Equation 9) B=LN,

where **L** and **N** are the differential operator and shape function matrix, and they have the following forms for 3D problems:

(Equation 10) $$L={\left[\begin{array}{cccccc} \frac{\partial }{\partial x} & 0 & 0 & \frac{\partial }{\partial y} & 0 & \frac{\partial }{\partial z}\\ 0 & \frac{\partial }{\partial y} & 0 & \frac{\partial }{\partial x} & \frac{\partial }{\partial z} & 0\\ 0 & 0 & \frac{\partial }{\partial z} & 0 & \frac{\partial }{\partial y} & \frac{\partial }{\partial x} \end{array}\right]}^{T}\text{,}$$

(Equation 11) $$N=\left[N_{2},N_{2},N_{3},...,N_{8}\right]\text{,}$$

(Equation 12) $$N_{k}=\left[\begin{array}{ccc} N_{k} & 0 & 0\\ & N_{k} & 0\\ 0 & 0 & N_{k} \end{array}\right],k=1,2,...,8\text{,}$$

(Equation 13) $$\begin{array}{l} N_{1}=\left(1-\varsigma_{1}\right)\left(1-\varsigma_{2}\right)\left(1-\varsigma_{3}\right)/8,N_{2}=\left(1+\varsigma_{1}\right)\left(1-\varsigma_{2}\right)\left(1-\varsigma_{3}\right)/8\\ N_{3}=\left(1+\varsigma_{1}\right)\left(1+\varsigma_{2}\right)\left(1-\varsigma_{3}\right)/8,N_{4}=\left(1-\varsigma_{1}\right)\left(1+\varsigma_{2}\right)\left(1-\varsigma_{3}\right)/8\\ N_{5}=\left(1-\varsigma_{1}\right)\left(1-\varsigma_{2}\right)\left(1+\varsigma_{3}\right)/8,N_{6}=\left(1+\varsigma_{1}\right)\left(1-\varsigma_{2}\right)\left(1+\varsigma_{3}\right)/8\\ N_{7}=\left(1+\varsigma_{1}\right)\left(1+\varsigma_{2}\right)\left(1+\varsigma_{3}\right)/8,N_{8}=\left(1-\varsigma_{1}\right)\left(1+\varsigma_{2}\right)\left(1+\varsigma_{3}\right)/8 \end{array}\text{,}$$

where (*ς*_1_,*ς*_2_,*ς*_3_) is the natural coordinate system in the finite element method.^105^ The nodal force **P** is defined as

(Equation 14) $$P=\sum_{i=1}^{N}\int_{\Omega_{i}}N^{T}p\;d\Omega_{i,}$$

where **p** is the external loads. The biomechanical active force is expressed as:

(Equation 15) $$P_{\text{bio}-\text{chemo}}=\sum_{i=1}^{N}\int_{\Omega_{i}}\text{BD}\left(E_{i}\right)\varepsilon_{a}d\;\Omega_{i}\text{.}$$

##### Topology design variables for different tissues

To distinguish different voxel types in the digital tissue, we introduce two vectors $x_{1}={\left(x_{1,1},x_{1,2},...,x_{1,N}\right)}^{T},x_{1,i}\in \left[0,1\right]$ and $x_{2}={\left(x_{2,1},x_{2,2},...,x_{2,N}\right)}^{T},\;x_{2,\;i}\in \left[0,1\right]$. Various types of voxels can be defined through a logical combination of **x**_1_ and **x**_2_ using a topology optimization method.^11^ The topology design variables for the finite element at growth time *t*_*k*_ are denoted as $x_{1<t_{k}>}$ and $x_{2<t_{k}>}$. The *i*th voxel element represents void or voxel, characterized by topological design variables $x_{1,i<t_{k}>}=0$ or $x_{1,i<t_{k}>}=1$. Specially, the *i*th voxel element is defined as the red or green voxels based on values of $x_{1,i<t_{k}>}\;\text{and}\;x_{1,i<t_{k}>}\;x_{2,i<t_{k}>}=1$ or $x_{1,i<t_{k}>}\;\text{and}\;x_{1,i<t_{k}>}\left(1-x_{2,i<t_{k}>}\right)=1$, respectively.^11^

The global stiffness matrix and bio-chemo force at *t*_*k*_ are expressed as

(Equation 16) $$K\left(v,E_{i_{<t_{k}>}}\right)=\sum_{i=1}^{N}k_{i<t_{k}>}=\sum_{i=1}^{N}\int_{\Omega_{i}}B^{T}D\left(v,E_{i_{<t_{k}>}}\right)Bd\Omega \text{,}$$

(Equation 17) $$P_{\text{bio}-\text{chemo}<t_{k}>}=\sum_{i=1}^{N}\int_{\Omega_{i}}\text{BD}\left(E_{i<t_{k}>}\right)\varepsilon_{a}\left(\alpha_{i<t_{k}>}\right)d\;\Omega_{i}\text{,}$$

(Equation 18) $$u_{<t_{k}>}=K_{<t_{k}>}^{-1}\left(P_{<t_{k}>}+P_{\text{bio}-\text{chemo}<t_{k}>}\right)\text{,}$$

where Young’s modulus ${E_{i}}_{<t_{k}>}$ and active contractile coefficients $\alpha_{i<t_{k}>}$ of the *i*th element at *t*_*k*_ growth step are represented as^11^

(Equation 19) $${E_{i}}_{<t_{k}>}\left({x_{1}}_{<t_{k}>},x_{2<t_{k}>}\right)=\rho_{1,i}^{\eta_{1}}\left({x_{1}}_{<t_{k}>},x_{2<t_{k}>}\right)\left\{\rho_{2,i}^{\eta_{2}}\left(x_{1<t_{k}>},x_{2<t_{k}>}\right)E_{c}+\left[1-\rho_{2,i}^{\eta_{2}}\left(x_{1<t_{k}>},x_{2<t_{k}>}\right)\right]E_{e}\right\}\text{,}$$

(Equation 20) $$\alpha_{i<t_{k}>}\left(x_{1<t_{k}>},x_{2<t_{k}>}\right)=\rho_{1,i}^{\eta_{1}}\left(x_{1<t_{k}>},x_{2<t_{k}>}\right)\rho_{2,i<t_{k}>}^{\eta_{2}}\left(x_{1},x_{2}\right)\alpha \text{,}$$

where *E*_c_ and *E*_e_ are the basal elastic modulus of the red and green voxels (representing the cardiomyocyte and epidermal cells), respectively. For instance, *E*_c_ = 100 kPa and *E*_e_ = 10 kPa(*Xenopus laevis*).^11^ During the optimization process, penalty factors defined as *η*_1_ = *η*_2_ = 4.0 are introduced in the material interpolation model to guide the values of **x**_1_ and **x**_2_ toward to 0 or 1.

To avoid the checkboard patterns commonly encountered in the solid - interpolation - material - penalty (SIMP) method, filtering operators are employed

(Equation 21) $$\rho_{1,i<t_{k}>}=\frac{\sum_{j\in N_{i}}w\left(r_{i},r_{j}\right)v_{j}x_{1,j<t_{k}>}}{\sum_{j\in N_{i}}w\left(r_{i},r_{j}\right)v_{j}}\text{,}$$

(Equation 22) $$\rho_{2,i<t_{k}>}=\frac{\sum_{j\in N_{i}}w\left(r_{i},r_{j}\right)v_{j}x_{2,j<t_{k}>}}{\sum_{j\in N_{i}}w\left(r_{i},r_{j}\right)v_{j}}\text{,}$$

where $N_{i}=\left\{\;j=|\left\|r_{i}-r_{j}\right\|\leq R\left(t_{k}\right)\;\right\}$, *R*(*t*_*k*_) represents the filtering radius, which evolves over time according to the relation *R*(*t*_*k*_) = *n*_*x*_(*t*_*k*_)/*n*_*x*_(*t*_0_)*R*_0_, where *R*_0_ is the initial filtering radius of the embryoid and can be modified in different scenarios. ***r***_*i*_ and ***r***_*j*_ are the position vectors of elements *i* and *j* (*j*∈*N*_*i*_), respectively. $w\left(r_{i},r_{j}\right)=\max\left(0,R\left(t\right)-\left\|r_{i}-r_{j}\right\|\right)$ is the weight factor. After filtering, the physical densities are also constrained within the interval [0, 1]. Under this condition, we have

(Equation 23) $${E_{i}}_{<t_{k}>}\left({\rho_{1}}_{<t_{k}>},\rho_{2<t_{k}>}\right)=\rho_{1,i}^{\eta_{1}}\left({\rho_{1}}_{<t_{k}>},x_{2<t_{k}>}\right)\left\{\rho_{2,i}^{\eta_{2}}\left(\rho_{1<t_{k}>},\rho_{2<t_{k}>}\right)E_{c}+\left[1-\rho_{2,i}^{\eta_{2}}\left(\rho_{1<t_{k}>},\rho_{2<t_{k}>}\right)\right]E_{e}\right\}\text{,}$$

(Equation 24) $$\alpha_{i,m_{2<t_{k}>}}\left(\rho_{1<t_{k}>},\rho_{2<t_{k}>}\right)=\rho_{1,i}^{\eta_{1}}\left(\rho_{1<t_{k}>},\rho_{2<t_{k}>}\right)\rho_{2,i<t_{k}>}^{\eta_{2}}\left(\rho_{1},\rho_{2}\right)\alpha \text{.}$$

##### Spatiotemporal topology optimization

At the growth time *t*_*k*_, the optimization formula of the organ design space is given by

(Equation 25) $$\begin{array}{l} \text{for}\;t_{k}=t_{0}:t_{G}\;\left(\%\;\text{growth}\right)\\ \begin{array}{l} \text{find}:x_{1<t_{k}>}={\left(x_{1,1},x_{1,2},...,x_{1,N}\right)}^{T},x_{2<t_{k}>}={\left(x_{2,1},x_{2,2},...,x_{2,N}\right)}^{T}\\ \max_{x_{1},x_{2}}:J_{\left(t_{k}\right)}=L^{T}u_{t_{k}}\\ s.t.:\left\{ \begin{array}{l} K_{\left(t_{k}\right)}\left({\rho_{1}}_{<t_{k}>},\rho_{2<t_{k}>}\right)u_{t_{k}}=P_{\text{bio}-\text{chemo}<t_{k}>}\left({\rho_{1}}_{<t_{k}>},\rho_{2<t_{k}>}\right),\\ V_{c\left(t_{k}\right)}\left({\rho_{1}}_{<t_{k}>},\rho_{2<t_{k}>}\right)=\sum_{i=1}^{N}{\rho_{1}}_{<t_{k}>}{\rho_{2}}_{<t_{k}>}\leq V_{1}^{*},\\ V_{e\left(t_{k}\right)}\left({\rho_{1}}_{<t_{k}>},\rho_{2<t_{k}>}\right)=\sum_{i=1}^{N}{\rho_{1}}_{<t_{k}>}\left(1-{\rho_{2}}_{<t_{k}>}\right)\leq V_{2}^{*},\\ x_{\min}\leq x_{1,i<t_{k}>}\leq 1,x_{\min}\leq x_{2,i<t_{k}>}\leq 1,i\in \left[1,2,\cdots ,N\right], \end{array}\right. \end{array}\\ x_{1<t_{k}>},x_{2<t_{k}>}\to x_{1<t_{k+1}>},x_{2<t_{k+1}>}\\ \text{end} \end{array}\text{,}$$

where $K_{<t_{k}>}$ and $u_{<t_{k}>}$ denote the global stiffness matrix and displacement vector at *t*_*k*_ moment,^11^ respectively; **L** signifies the corresponding nodal index vector. $J_{\left(t_{k}\right)}$ is the optimization objective at *t*_*k*_ growth step. At each growth time *t*_*k*_, the volume fraction of the red and green voxels in the updated design space is $V_{1}^{*}=10\%$ and $V_{2}^{*}=10\%$, respectively. A small damping factor *x*_*min*_ = 10^−6^ is introduced to prevent the numerical singularity. During the optimization, the nodal force **p** is neglected. The intermediate objective is to obtain the converged design variables $x_{1<t_{k}>}$ and $x_{2<t_{k}>}$, with the initial values of them at t_0_ being random values between (0, 1].

To derive the optimization results in Equation 25, the topology design variables $x_{1<t_{k}>}$ and $x_{2<t_{k}>}$ need to be updated iteratively. Using gradient-based optimization method, such as Method of Moving Asymptotes (MMA),^106^ the optimal search directions are determined by the gradient information of the objective function and constraints with respect to the design variables.

(Equation 26) $$\begin{array}{l} \frac{\partial J}{\partial x_{1,i<t_{k}>}}=\sum_{j\in N_{i}}-{u_{j}}^{T}\frac{\partial E_{j}\left(\rho_{1<t_{k}>},\rho_{2<t_{k}>}\right)}{\partial x_{1,i<t_{k}>}}k_{i}u_{i}+...\\ \sum_{j\in N_{i}}{u_{j}}^{T}\frac{\partial \left[E_{j}\left(\rho_{1<t_{k}>},x_{2<t_{k}>}\right)\alpha_{j}\left(\rho_{1<t_{k}>},\rho_{2<t_{k}>}\right)\right]}{\partial x_{1,i<t_{k}>}}{\tilde{P}}_{i,\text{bio}-\text{chemo}<t_{k}>}\text{,}\\ \forall j\in \left\{j=1,2,3,...,n|\text{dist}\left(i,j\right)<R\left(t_{k}\right)\right\},i=1,2,...,N \end{array}$$

(Equation 27) $$\begin{array}{l} \frac{\partial J}{\partial x_{2,i<t_{k}>}}=\sum_{j\in N_{i}}-{u_{j}}^{T}\frac{\partial E_{j}\left(\rho_{1<t_{k}>},\rho_{2<t_{k}>}\right)}{\partial x_{2,i<t_{k}>}}k_{i}u_{i}+...\\ \sum_{j\in N_{i}}{u_{j}}^{T}\frac{\partial \left[E_{j}\left(\rho_{1<t_{k}>},x_{2<t_{k}>}\right)\alpha_{j}\left(\rho_{1<t_{k}>},\rho_{2<t_{k}>}\right)\right]}{\partial x_{2,i<t_{k}>}}{\tilde{P}}_{i,\text{bio}-\text{chemo}<t_{k}>} \end{array}\text{,}$$

where the terms $\partial E_{i<t_{k}>}/\partial x_{k,i<t_{k}>}$ and $\partial \left(E_{i<t_{k}>}\alpha_{i<t_{k}>}\right)/\partial x_{k,i<t_{k}>}$ can be computed from Equations 23 and 24 using the chain rule, and ${\tilde{P}}_{\text{bio}-\text{chemo}<t_{k}>}=P_{\text{bio}-\text{chemo}<t_{k}>}/\left(E_{i<t_{k}>}\alpha_{i<t_{k}>}\right)$; $\partial \left(\rho_{1,i<t_{k}>}\rho_{2,i<t_{k}>}\right)/\partial x_{1,i<t_{k}>}$, $\partial \left(\rho_{1,i<t_{k}>}\rho_{2,i<t_{k}>}\right)/\partial x_{2,i<t_{k}>}$ and $\partial \left[\rho_{1,i<t_{k}>}\left(1-\rho_{2,i<t_{k}>}\right)\right]/\partial x_{1,i<t_{k}>}$, $\partial \left[\rho_{1,i<t_{k}>}\left(1-\rho_{2,i<t_{k}>}\right)\right]/\partial x_{2,i<t_{k}>}$ can be calculated from (Equation 21), (Equation 22).

Additionally, the derivates of the volume fractions of red voxels and green voxels with respect to the design variables are

(Equation 28) $$\frac{\partial V_{c}\left(\rho_{1<t_{k}>},\rho_{2<t_{k}>}\right)}{\partial x_{1,i<t_{k}>}}=\sum_{j\in N_{i}}\frac{\partial \left(\rho_{1,j<t_{k}>}\rho_{2,j<t_{k}>}\right)\;}{\partial x_{1,i<t_{k}>}}\frac{s_{j}}{\Omega_{<t_{k}>}}\text{,}$$

(Equation 29) $$\frac{\partial V_{c}\left(\rho_{1<t_{k}>},\rho_{2<t_{k}>}\right)}{\partial x_{2,i<t_{k}>}}=\sum_{j\in N_{i}}\frac{\partial \left(\rho_{1,j<t_{k}>}\rho_{2,j<t_{k}>}\right)\;}{\partial x_{2,i<t_{k}>}}\frac{s_{j}}{\Omega_{<t_{k}>}}\text{,}$$

(Equation 30) $$\frac{\partial V_{e}\left(\rho_{1<t_{k}>},\rho_{2<t_{k}>}\right)}{\partial x_{1,i<t_{k}>}}=\sum_{j\in N_{i}}\frac{\partial \left[\rho_{1,j<t_{k}>}\left(1-\rho_{2,j<t_{k}>}\right)\right]\;}{\partial x_{1,i<t_{k}>}}\frac{s_{j}}{\Omega_{<t_{k}>}}\text{,}$$

(Equation 31) $$\frac{\partial V_{e}\left(\rho_{1<t_{k}>},\rho_{2<t_{k}>}\right)}{\partial x_{2,i<t_{k}>}}=\sum_{j\in N_{i}}\frac{\partial \left[\rho_{1,j<t_{k}>}\left(1-\rho_{2,j<t_{k}>}\right)\right]\;}{\partial x_{2,i<t_{k}>}}\frac{s_{j}}{\Omega_{<t_{k}>}}\text{,}$$

##### Optimization process

At the initial time *t*_0_, the dimensions of the initial design space are defined with *l*_*x*0_ = *l*_*z*0_ = 0.2 mm and *l*_*z*0_ = 0.4 mm, along with the specification of the number of cubic finite elements with *n*_*x*0_ = *n*_*z*0_ = 20 and *n*_*y*0_ = 40 (case 8 in Figure 2A). Following the growth rule outlined in Equation 3, the evolving embryoid exhibits an increase in voxel numbers over time (Figure S8). The edge increment for each growth step along different directions is denoted as Δ*l*_*i*_ = *g*_*i*_Δ*tl*_e_(*i* = *x*,*y*,and*z*), indicating an isotropic growth rule applied to the embryoid with *g*_*x*_ = *g*_*y*_ = *g*_*z*_ and Δ*l*_*x*_ = Δ*l*_*z*_. Importantly, the element increments along the *x* and *z* directions are even numbers. As a result, the modified edge increments are presented, and the element increments are calculated in Table S2 ($\Delta n_{i}=\Delta {\tilde{l}}_{i}/l_{e},i=x,y,\text{and}\;z$). For instance, the mature individual reaches its maximum height in the *y*-direction, measuring *l*_*y*_ = 7.2 mm. Correspondingly, the count of elements aligned with the *y* direction is *n*_*y*_ = 72. The height is constrained by the 64 GB of the computer RAM; with improved RAM capacity, the height of the mature optimized vertebrate can reach centimeters (Figure S34). The entire optimized workflow for the spatiotemporal topological evolution is depicted in Figures S7 and S9, and consists of two primary components.

1. Algorithm 1 illustrates a topological optimization approach (as elaborated in the subsequent section) for the design of a multi-voxel functional embryoid (at *t*_0_) within a defined design space (Figure S4). During each growth iteration, the convergence criterion for to the topology optimization process can be met by reaching a maximum iteration count of *L* ≤ 100.
  Algorithm 1 **Optimization for digital embryoid at t0**
  *n*_*x*_,*n*_*y*_,*n*_*z*_,Δ*C*,*α*,*E*_c_,*E*_e_,*v* % Initial parameters
  $R_{0},\eta_{1},\eta_{2},V_{1}^{*},V_{2}^{*}$  % Optimization parameters
$x_{1<t_{0}>}=\left\{x_{1,i<t_{0}>}=0.6,i=1,2,...,N\right\},x_{2<t_{0}>}=\left\{x_{2,i<t_{0}>}=0.6,i=1,2,...,N\right\}$
  While L ≤ 100
  $K_{\left(t_{0}\right)}\left({\rho_{1}}_{<t_{0}>},\rho_{2<t_{0}>}\right)u_{\left(t_{0}\right)}=P_{\text{bio}-\text{chemo}<t_{0}>}\left({\rho_{1}}_{<t_{0}>},\rho_{2<t_{0}>}\right),\to u_{\left(t_{0}\right)}$ % Compute displacement fields
  $V_{c\left(t_{0}\right)}\left({\rho_{1}}_{<t_{0}>},\rho_{2<t_{0}>}\right),V_{e\left(t_{0}\right)}\left({\rho_{1}}_{<t_{0}>},\rho_{2<t_{0}>}\right)$  % Compute volume fraction
  $\begin{array}{l} \partial J/\partial x_{1,i<t_{0}>},\partial J/\partial x_{2,i<t_{0}>},i=1,2,\ldots ,N\\ \partial V_{c<t_{0}>}/\partial x_{1,i<t_{0}>},\partial V_{c<t_{0}>}/\partial x_{2,i<t_{0}>},\partial V_{e<t_{0}>}/\partial x_{1,i<t_{0}>},\partial V_{e<t_{0}>}/\partial x_{2,i<t_{0}>} \end{array}$ % Compute sensitivity information
  start the optimization operator MMA  % Inner optimization starts
  $x_{1<t_{0}>}^{\text{new}},x_{2<t_{0}>}^{\text{new}}\to \rho_{1<t_{0}>}^{\text{new}},\rho_{2<t_{0}>}^{\text{new}}$  % Update design variables
  end
  Output virtual vertebrate embryoids
2. The optimized configuration is then integrated into the spatiotemporal evolution module (Algorithm 2), which incorporates the growth memory effect. The design space is updated according to Equation 3, with previous optimization results serving as inputs for the new design space. This updated design space is visually represented in Figures S7 and S8. The same topology optimization methodology is then applied to this updated design space, and the optimization process continues until convergence is achieved. Following convergence, the optimization outcomes are assessed to ensure compliance with the growth termination conditions. If these conditions are not met, the design space is further updated, and a new optimization cycle begins. Conversely, if the growth termination conditions are satisfied, the program concludes, providing the results of the spatiotemporal topological evolution. All numerical optimization scenarios are executed using MATLAB on a workstation equipped with Intel(R) Core (TM) i9-10900X CPU @ 3.70 GHz and 64 RAM.
  Algorithm 2 **Spatiotemporal topology optimization with k ≥ 1**
  while *t*_*k*_ ≤ *t*_*M*_  % *M* is the maximum growth steps
  $n_{x<t_{k}>},n_{y<t_{k}>},n_{z<t_{k}>}$, $R\left(t_{k}\right),V_{1<t_{k}>}^{*},V_{2<t_{k}>}^{*}$
  $\rho_{1<t_{k-1}>},\rho_{2<t_{k-1}>}\to x_{1<t_{k}>},x_{2<t_{k}>}$  % Initial values of design values at *t*_*k*_
  While L≤100
  $u_{t_{k}}=K_{t_{k}}^{-1}\left({\rho_{1}}_{<t_{k}>},\rho_{2<t_{k}>}\right)P_{\text{bio}-\text{chemo}<t_{k}>}\left({\rho_{1}}_{<t_{k}>},\rho_{2<t_{k}>}\right)$ % Compute displacement fields
  $V_{c\left(t_{k}\right)}\left({\rho_{1}}_{<t_{k}>},\rho_{2<t_{k}>}\right),V_{e\left(t_{k}\right)}\left({\rho_{1}}_{<t_{k}>},\rho_{2<t_{k}>}\right)$  % Compute volume fraction
  $\begin{array}{l} \partial J/\partial x_{1,i<t_{k}>},\partial J/\partial x_{2,i<t_{k}>},i=1,2,\ldots ,N\\ \partial V_{c<t_{k}>}/\partial x_{1,i<t_{k}>},\partial V_{c<t_{k}>}/\partial x_{2,i<t_{k}>},\partial V_{e<t_{k}>}/\partial x_{1,i<t_{k}>},\partial V_{e<t_{k}>}/\partial x_{2,i<t_{k}>} \end{array}$ % Compute sensitivity information
  start the optimization operator MMA  % Inner optimization starts
  $x_{1<t_{k}>}^{\text{new}},x_{2<t_{k}>}^{\text{new}}\to \rho_{1<t_{k}>}^{\text{new}},\rho_{2<t_{k}>}^{\text{new}}$  % Update design variables
  end
  Output results: $\rho_{1<t_{k}>}^{\text{new}},\rho_{2<t_{k}>}^{\text{new}}$, optimization history, virtual vertebrate vertebrates at *t*_*k*_
  *k* = *k*+1
  end
  Output virtual mature vertebrates

### Quantification and statistical analysis

In this study, morphological-geometric parameters of vertebrate animal samples were quantified from publicly available images (Figure 4). For each animal, image regions covering the head-to-dorsal spine were cropped and normalized to a uniform image height prior to measurement (Figure S21). The spinal profile extending from the head to the back region was traced as a curve using the curve-drawing tool in Microsoft PowerPoint (Figures S21 and S22); all subsequent measurement of dimensionless size parameters *h* and *w* were performed directly on this traced curve using the distance-measuring tool of the same software (Figure S23). *K*_S_ and $l_{h\_b}$ are the slope and relative height from animal’s back to its head, respectively. Each independent animal sample was measured once, and more than 60 vertebrates were included in this analysis (Figure S22; Tables S4 and S6).

## Supplemental information

Document S1. Figures S1–S36 and Tables S1–S5

Table S6. The morphological parameters of *K*_S_ and $\Delta l_{h\_b}$ are provided for each vertebrate, related to Figure 4
