# Supplementary material for: Biomechanics-guided computational design of virtual terrestrial vertebrate morphogenesis
Source: iScience. 2026 Oct 15;29(11):117876. doi: 10.1016/j.isci.2026.117876 (PMC13691557; doi:10.1016/j.isci.2026.117876)
Supplement: Supplementary file 1 [file mmc1.pdf]

iScience, Volume *29*

Supplemental information

Biomechanics-guided computational design of virtual terrestrial vertebrate morphogenesis

Hui-Kai Zhang, Xi-Qiao Feng, and Xing Xu

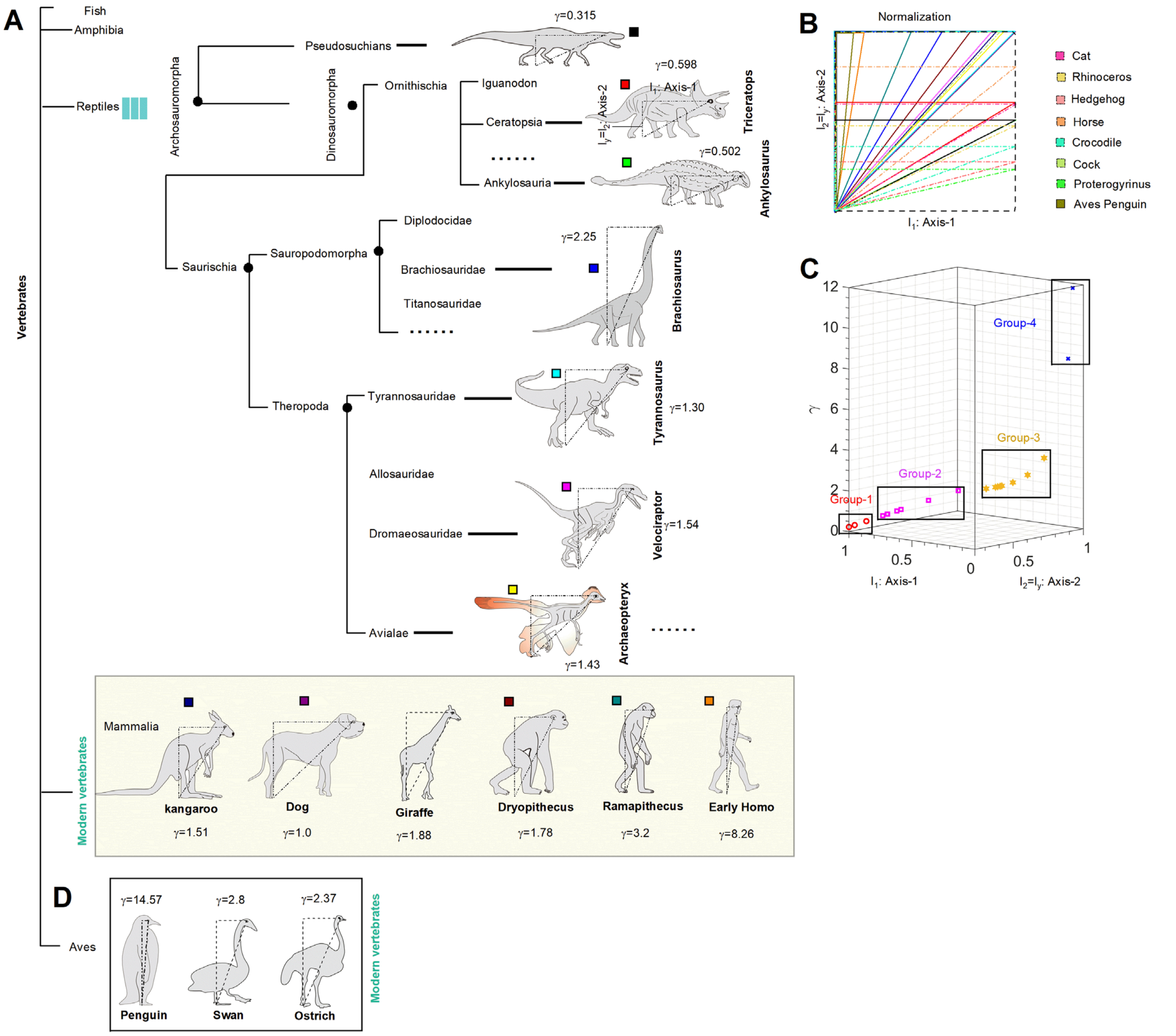

Figure S1. Analysis of body characteristics across ancient to modern vertebrates. (A) Representative vertebrate species (Animals are drawn by the first author based on the 3D animal models; Dinosaurs are from https://reptileevolution.com/). (B) and (C) Description of body characteristics in crawling vertebrates with $\gamma = l_2 / l_1$ (Table S1). The first group includes crocodiles, cynops, and lizards; the second and third groups represent the most common bipedal and quadrupedal crawling vertebrates, respectively; the fourth group encompasses upright-walking organisms such as Homo sapiens, humans, and penguins. (D) Modern birds represented from left to right: penguins, swans, and ostriches.

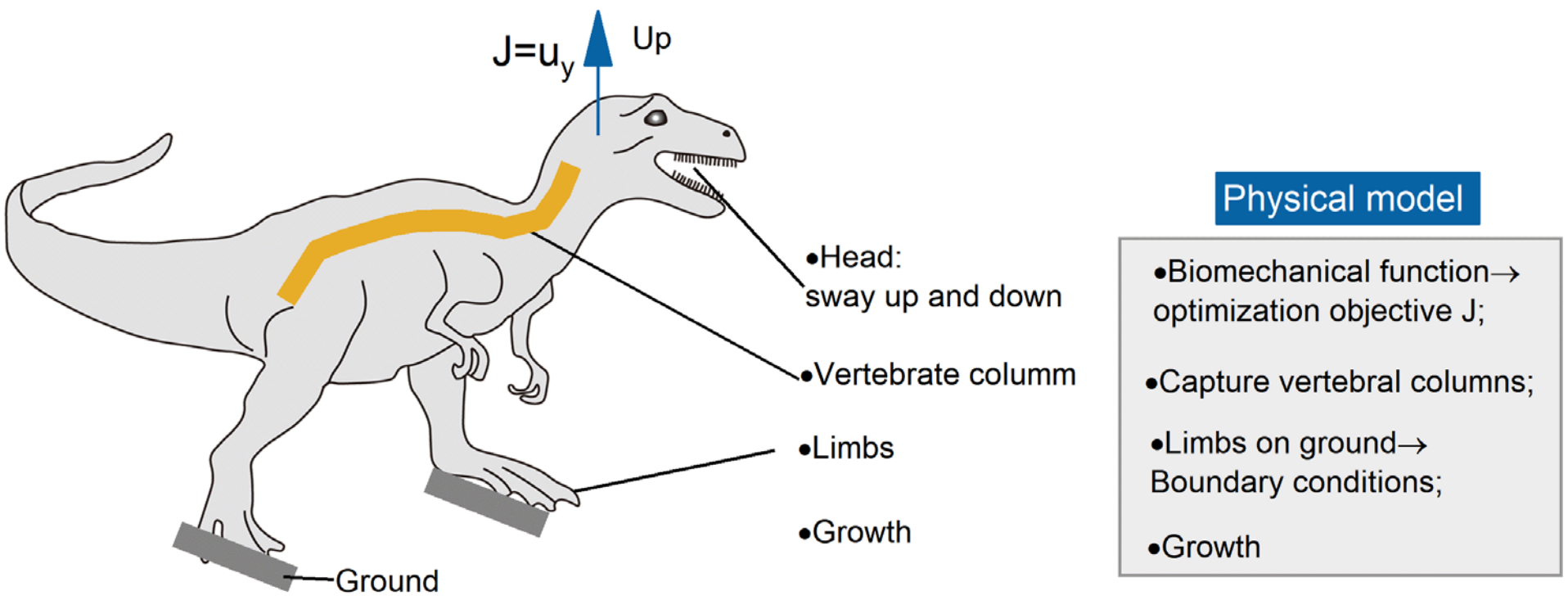

Figure S2. The biomechanical optimization model (right) should have the capacity to generate all the primary body characteristics of the vertebrate (left).

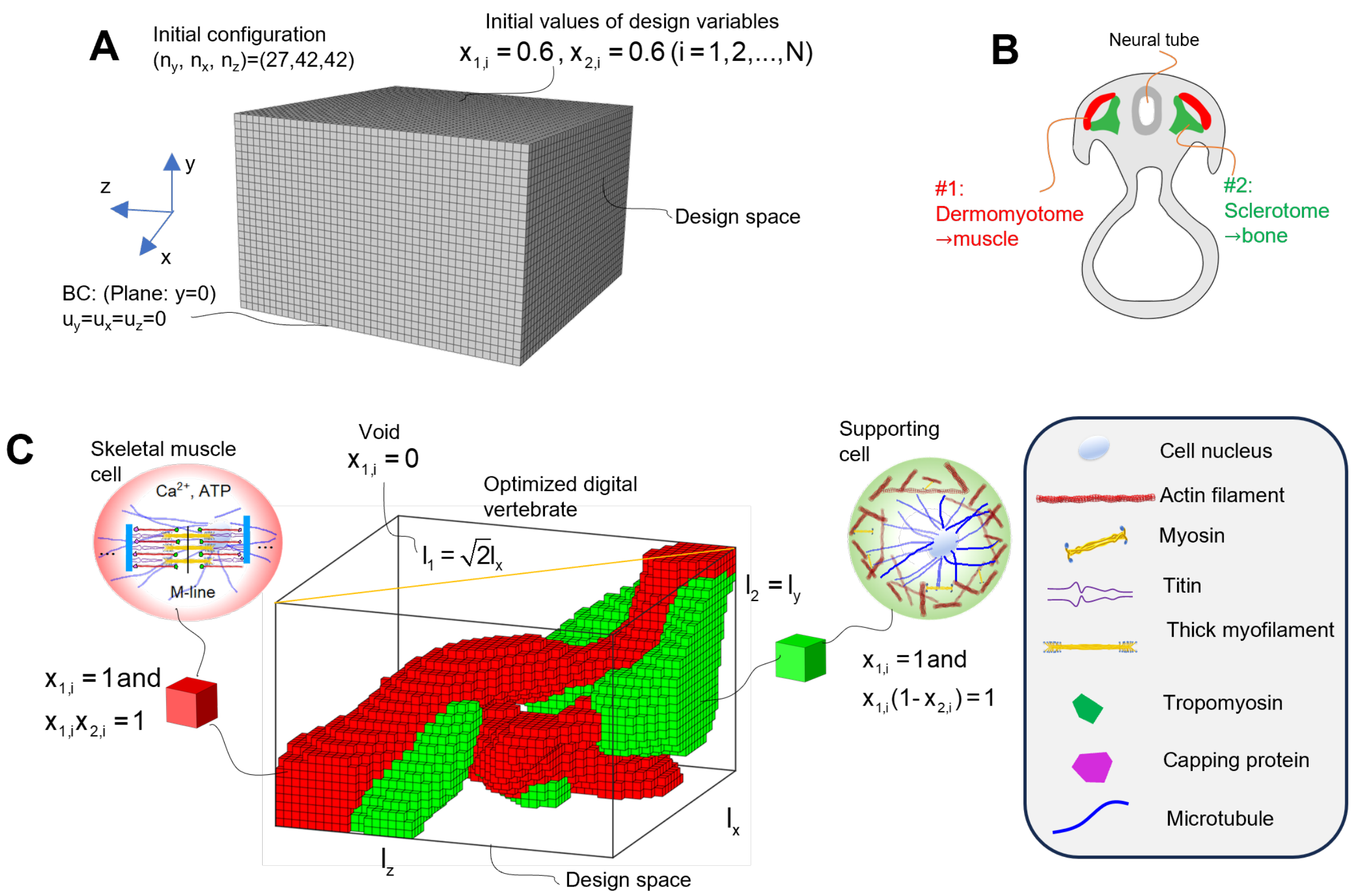

Figure S3. Topological variables to define different types of voxels. (A) Initial design space. (B) The original places of muscle (red) and bone (green) tissues in the early vertebrate embryo. (C) Red and green cubic voxel represents the skeleton and supporting voxels through the combination of design variables $x_{1,i}$ $(i = 1, 2, \ldots, N)$ and $x_{2,i}$; Red voxel has $x_{1,i} = 1.0$ and $x_{1,i} x_{2,i} = 1.0$; green voxel has $x_{1,i} = 1.0$ and $x_{1,i}(1 - x_{2,i}) = 1.0$, and void region has $x_{1,i} = 0$.

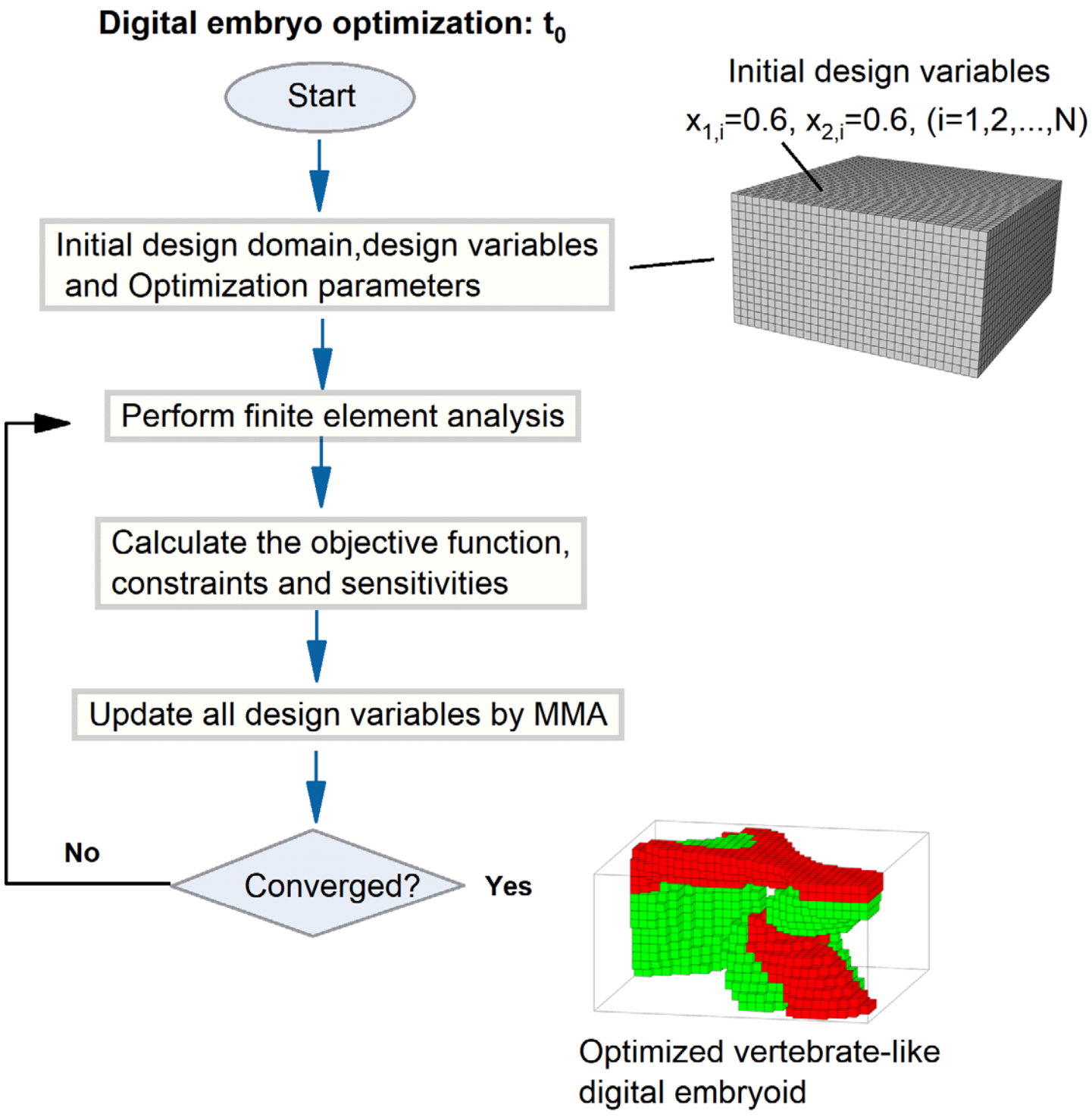

Figure S4. Diagram of the optimization process for the virtual vertebrate-like embryoids. At this state, the growth time is t_0.

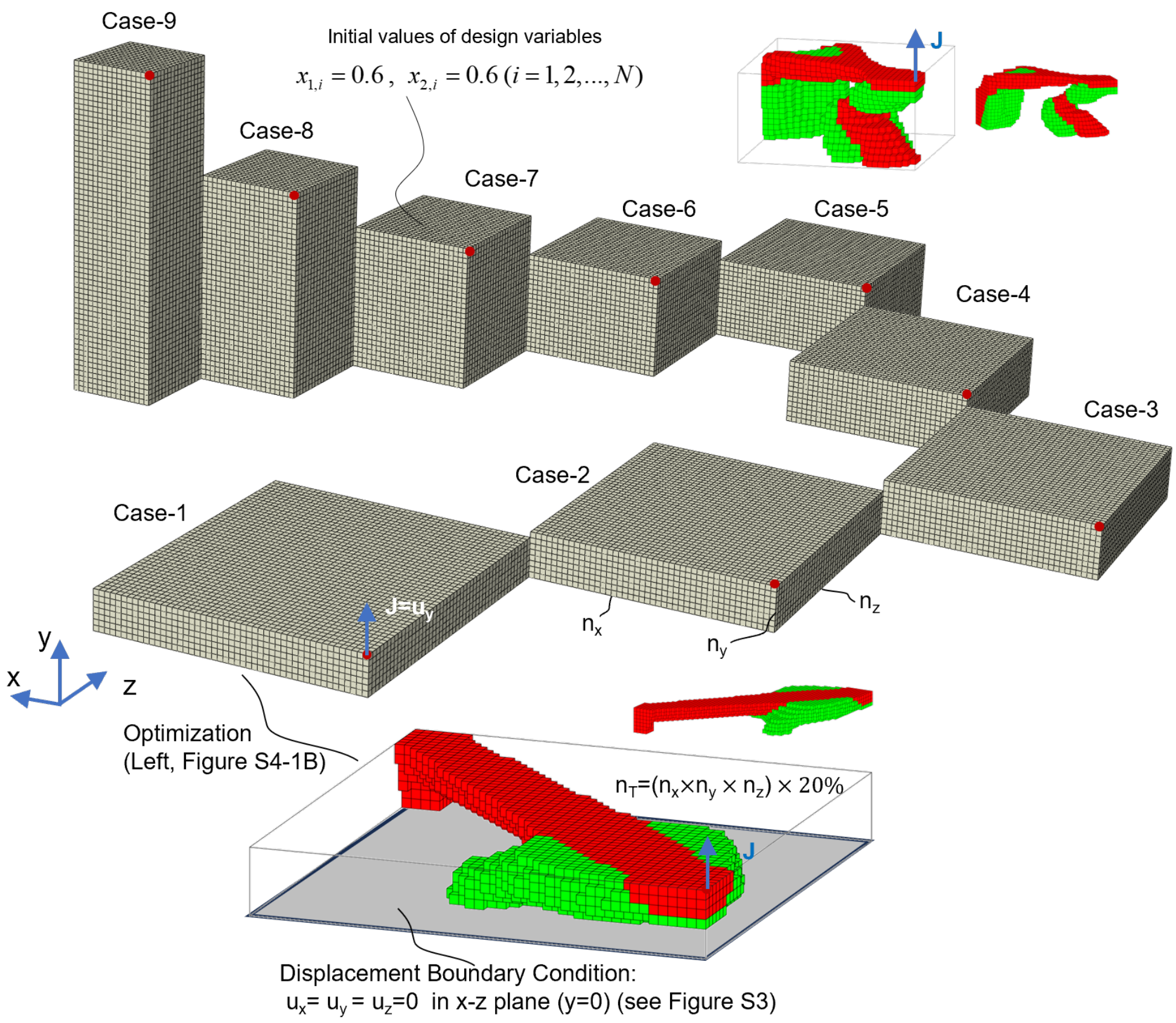

Figure S5. Initial optimization spaces for different digital embryoids (Figure 2A), which have the approximately about 1600 voxels. The values of (n_y, n_x, n_z) are listed in Table S2. Body parameters γ_{E} is computed as $\gamma_{\mathrm{E}} = n_y / \left(\sqrt{2} n_x\right)$. The optimization objective is defined as $J = \mathrm{u}_y$, u_y is the head displacement alongside the *y*-direction.

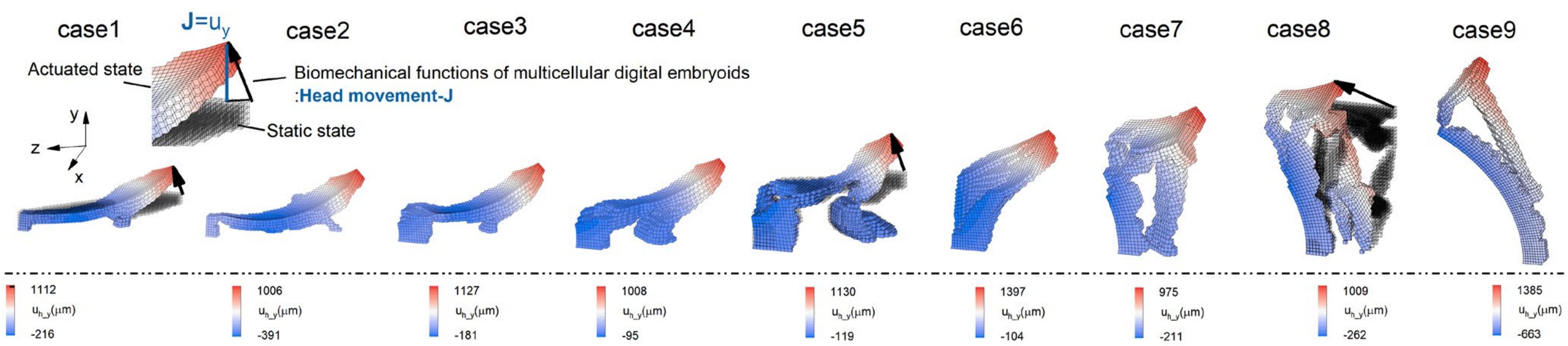

Figure S6. Whole body active actuations (Biomechanical functions) of the optimized vertebrate-like virtual embryoids.

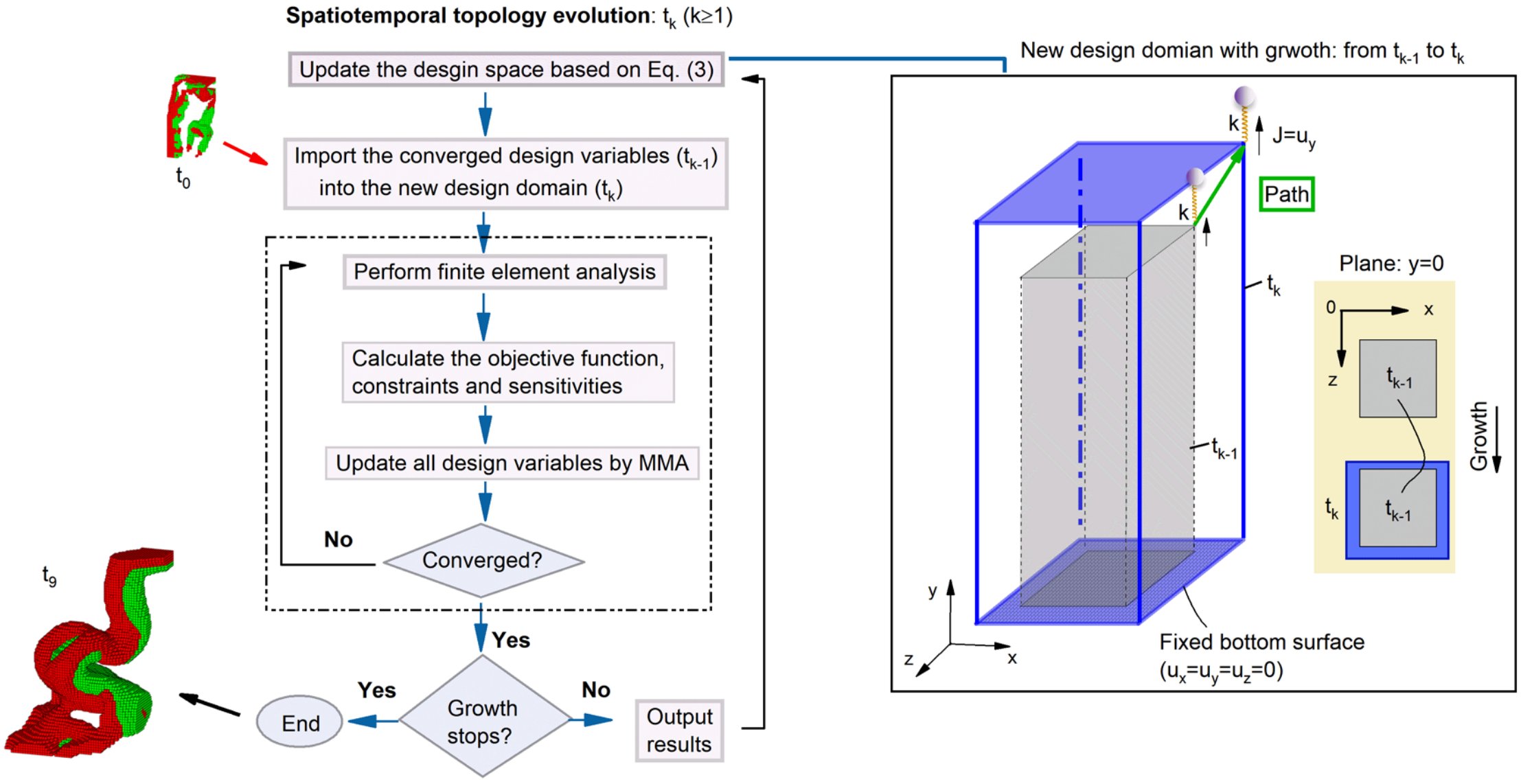

Figure S7. Diagram of the theory of spatio-temporal topological evolution. For example, at t_1 growth state, one optimized digital embryoids (at t_0) in Figures S5 should be imported to the new design space at t_1; consequently, it has $\boldsymbol{\rho}_{1<t_0>} \subseteq \mathbf{x}_{1<t_1>}, \boldsymbol{\rho}_{2<t_0>} \subseteq \mathbf{x}_{1<t_2>}$. This process continues until the growth stops.

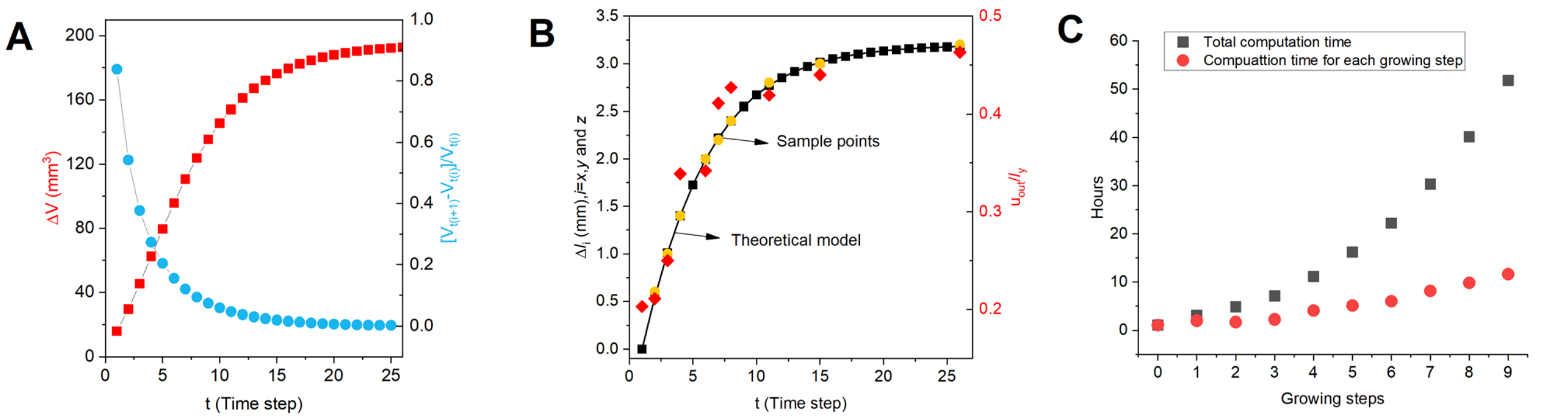

Figure S8. Changes of optimization spaces in Case 8. (A) Relationship between design space volume increments and volume change rates. (B) Edge increments over growth time. (C) Computational time for each growth step.

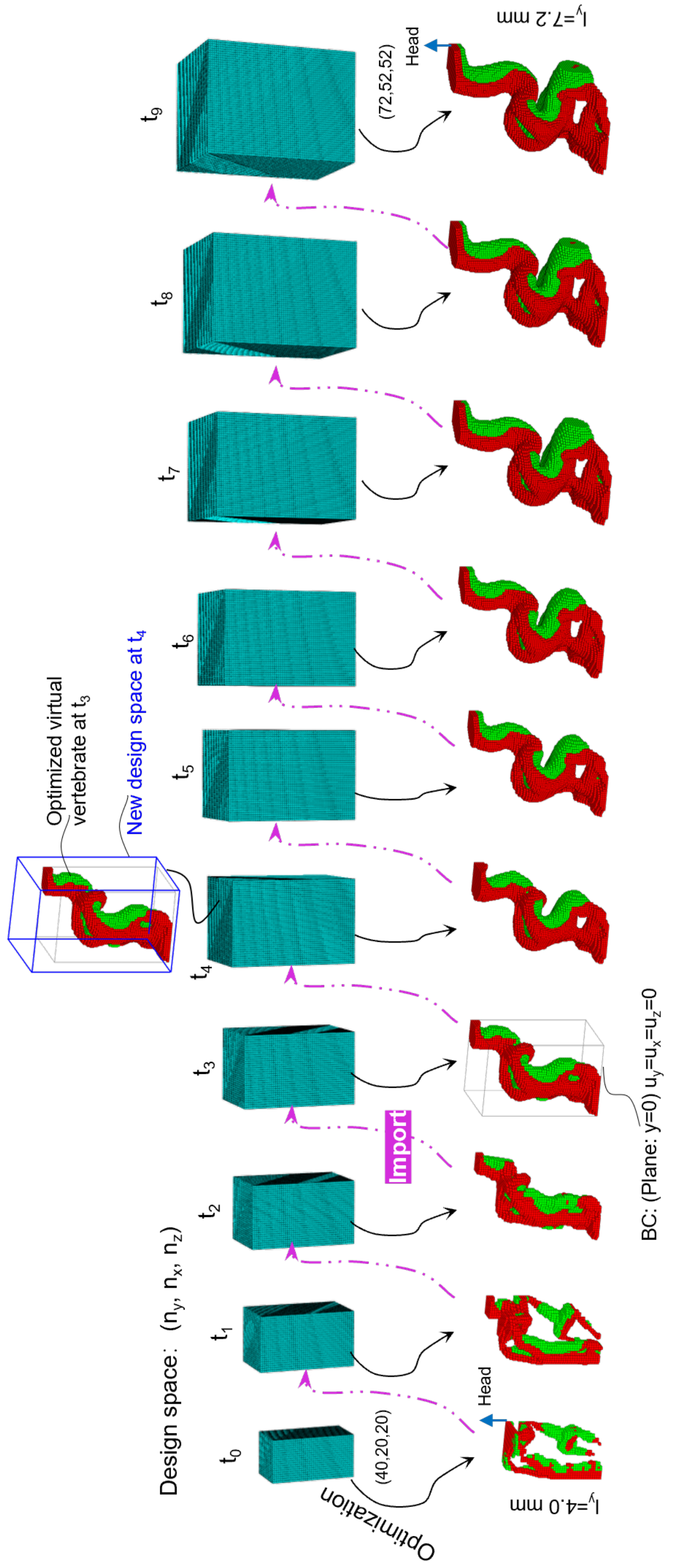
Design space: (n_y, n_x, n_z)
t_0
t_1
t_2
t_3
t_4
t_5
t_6
t_7
t_8
t_9
(40,20,20)
Optimization
Head
l_y=4.0 mm
Import
BC: (Plane: y=0) u_y=u_x=u_z=0
Optimized virtual vertebrate at t_3
New design space at t_4
(72,52,52)
Head
l_y=7.2 mm

Figure S9. Growth process of multi-voxel vertebrates predicted by the spatio-temporal topology evolution theory (Case 8, Figure 2A).

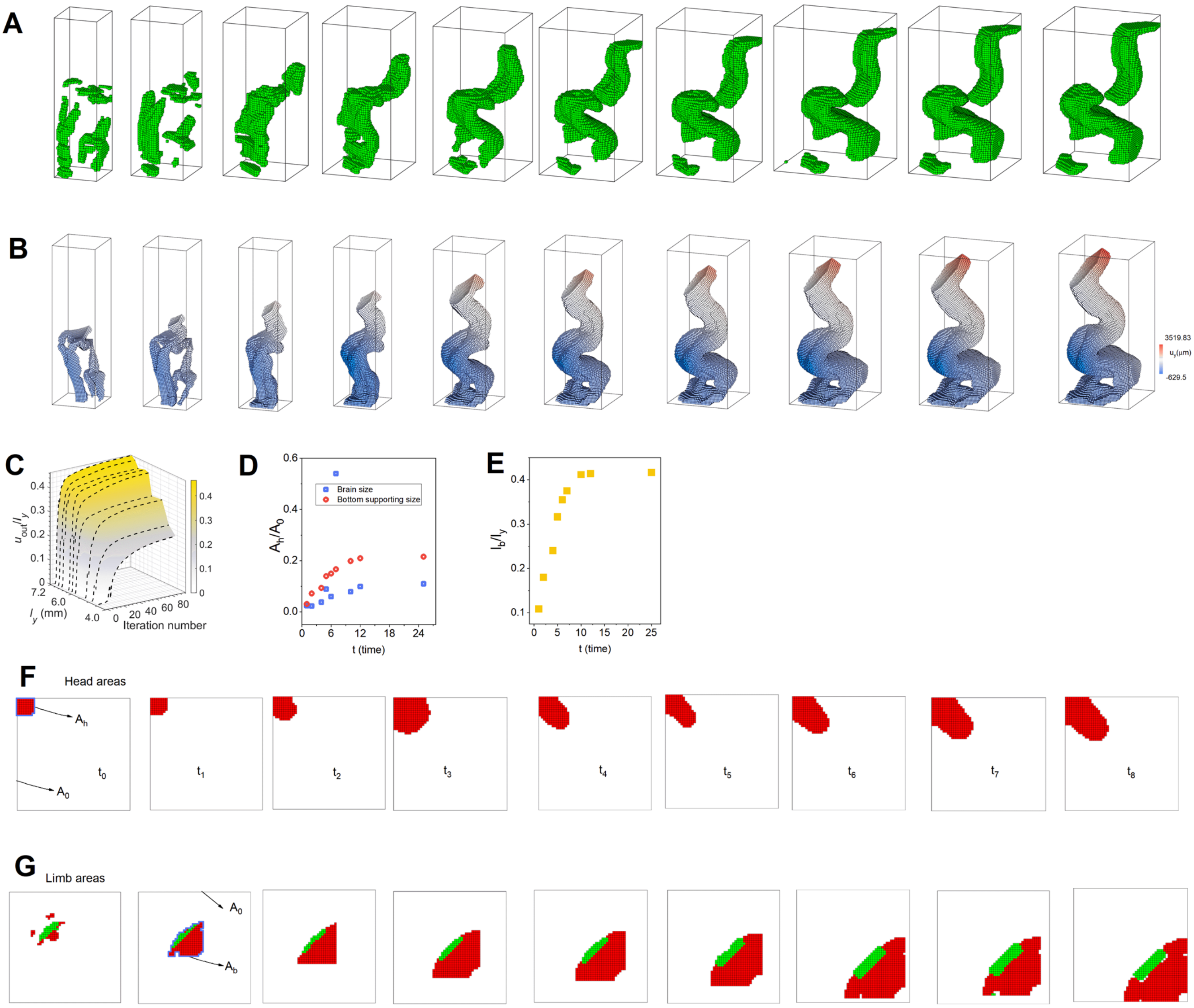

Figure S10. Analysis of optimization result. (A) Changes in the topological structure of supporting voxels during growth. (B) Deformation analysis of multi-voxel vertebrate organisms at different growth stages. (C) Functional output histories at different growing stages. (D) Head sizes A_b / A_0 with growth time. (E) Dorsal height l_b with growth time. (F) and (G) Multi-voxel topological changes in the top head and bottom limb with growth time.

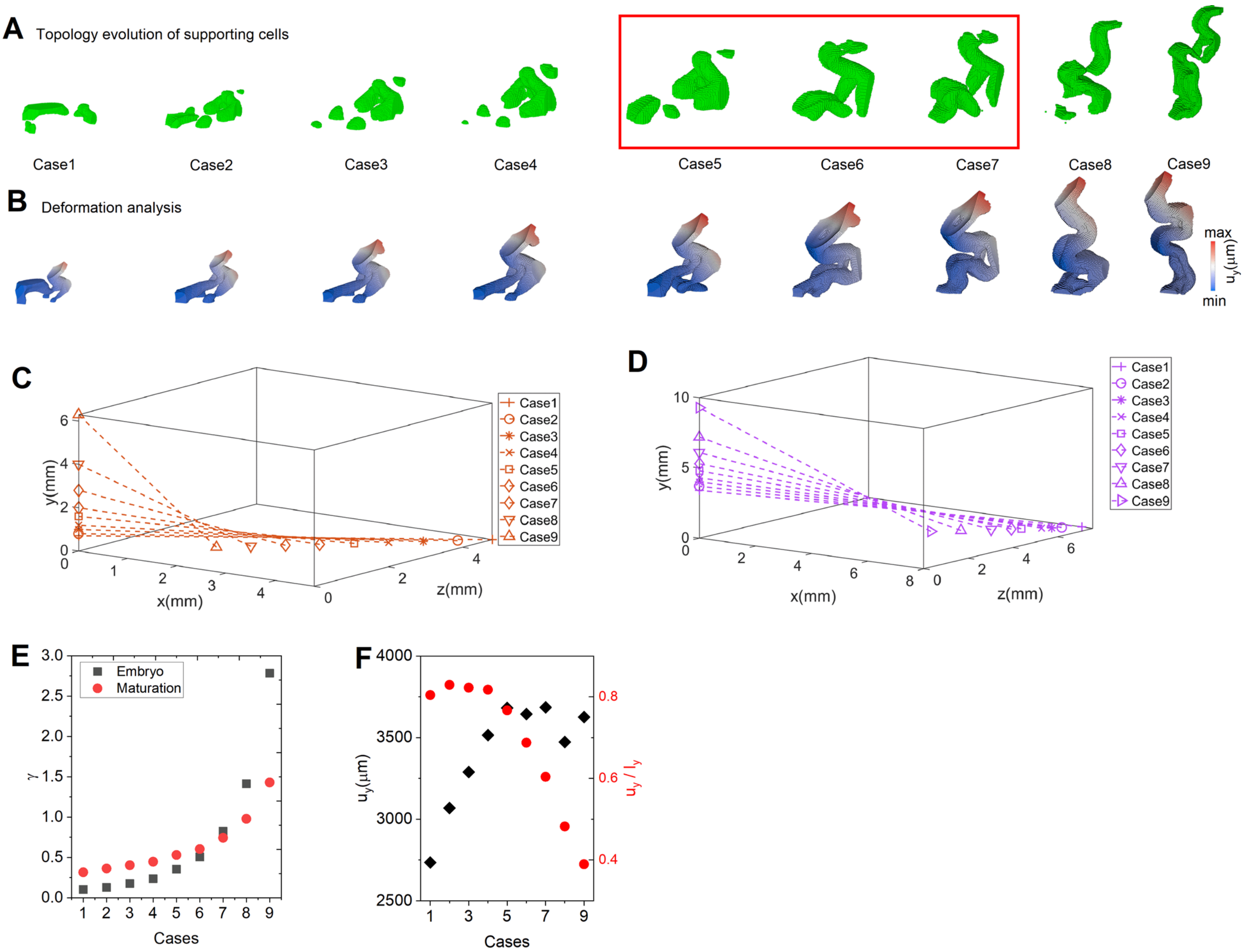

Figure S11. Vertebrate body plans. (A) Topological differences in supporting voxels driven by distinct embryoids. The topological evolution highlighted in the red box alters the limb characteristics of vertebrates. (B) Deformations of optimized vertebrates. (C), (D), and (E) Distribution of body plan characteristics in embryonic and mature vertebrates. (F) Head propulsions across all cases.

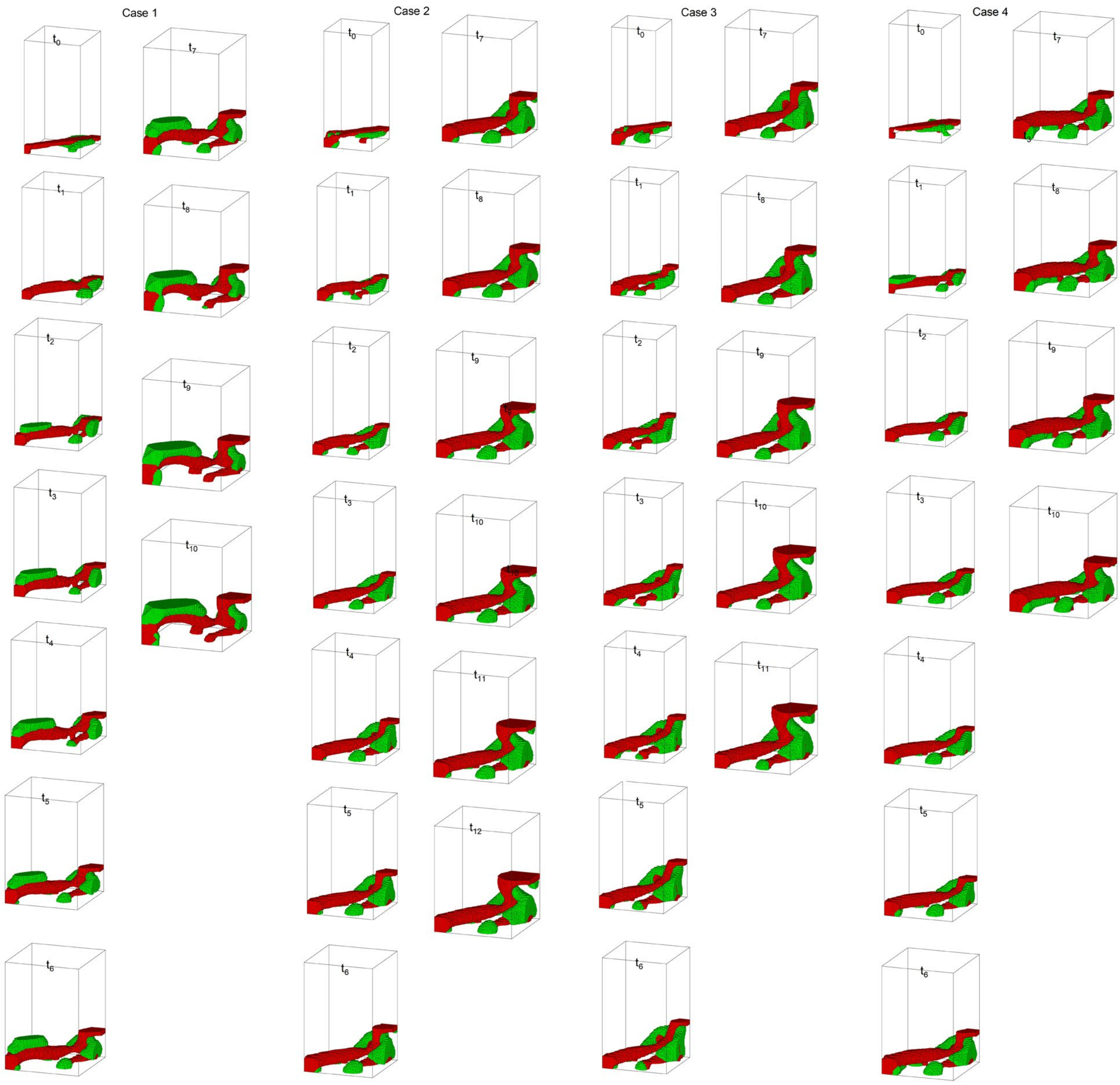

Figure S12. Topological evolutions for various optimized vertebrates.

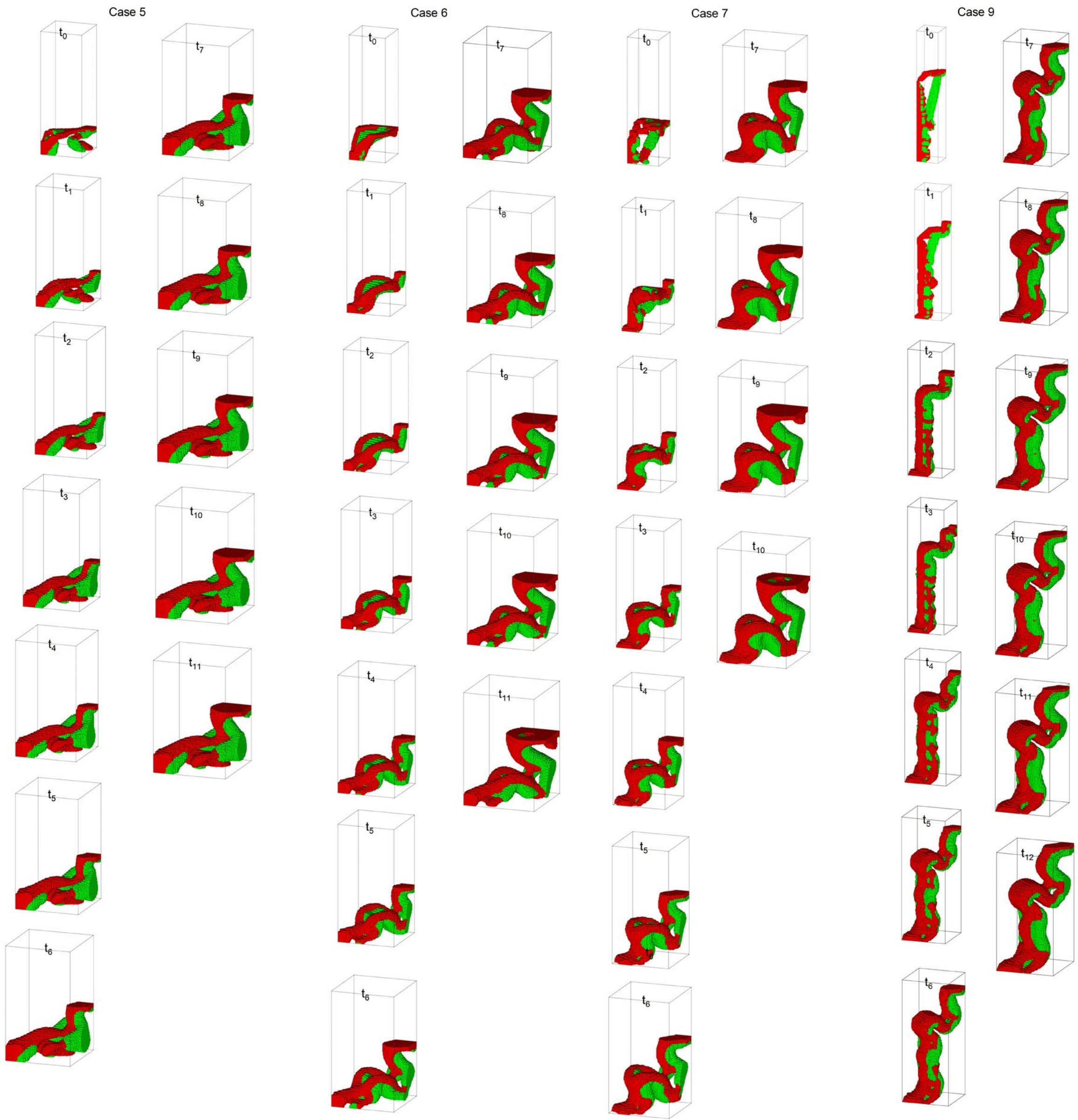

Figure S13. Topological evolutions for various optimized vertebrates.

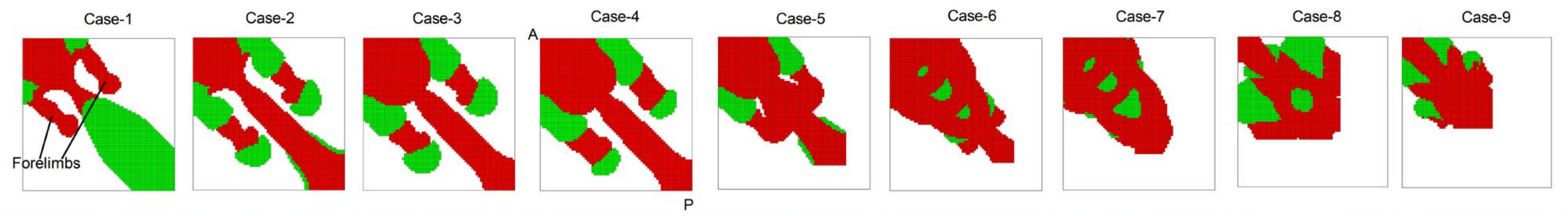

Figure S14. Vertical view of Figure 2A (Adult stage).

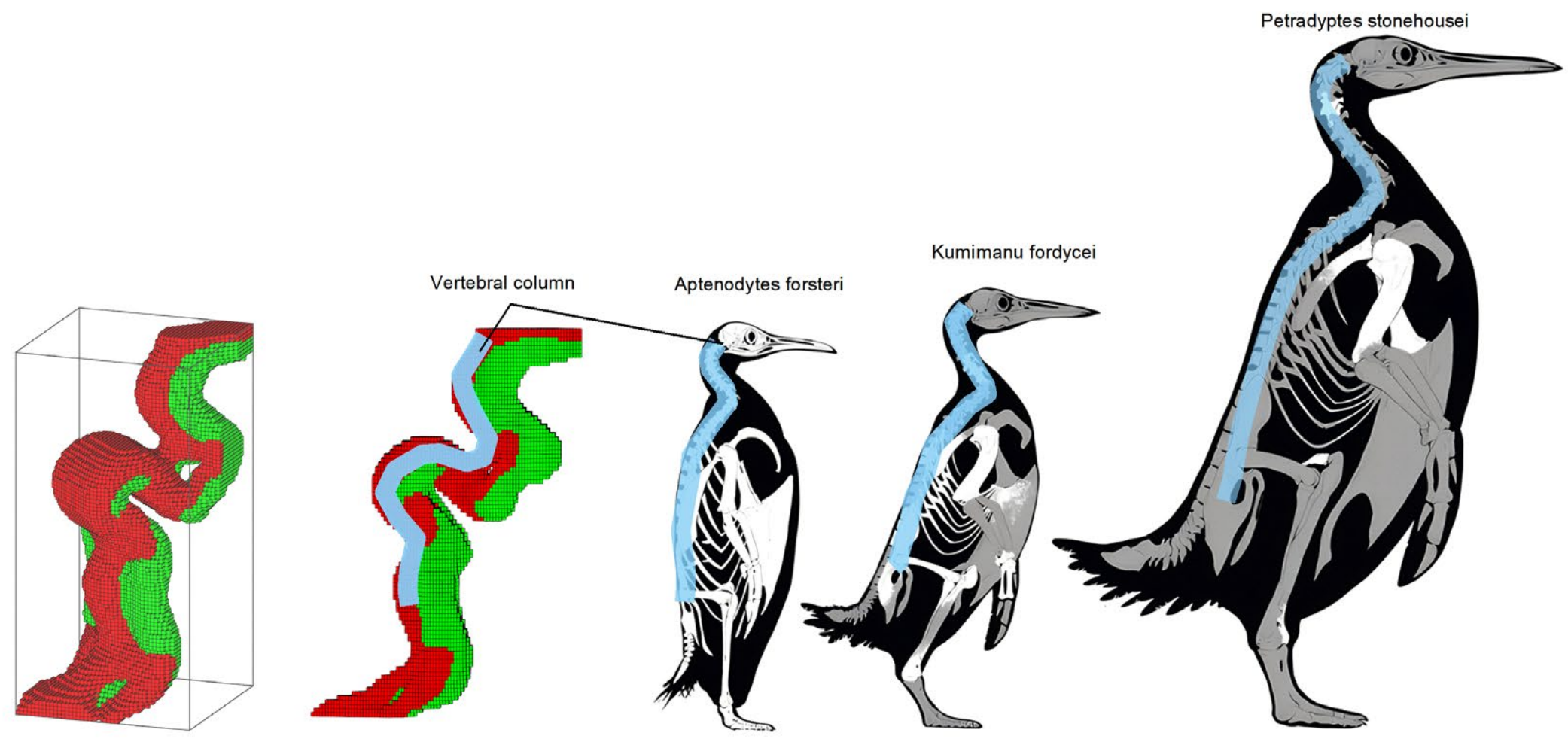

Figure S15. Optimized results in case 9 (Figure 2A) with the skeletal structure of penguins [1].

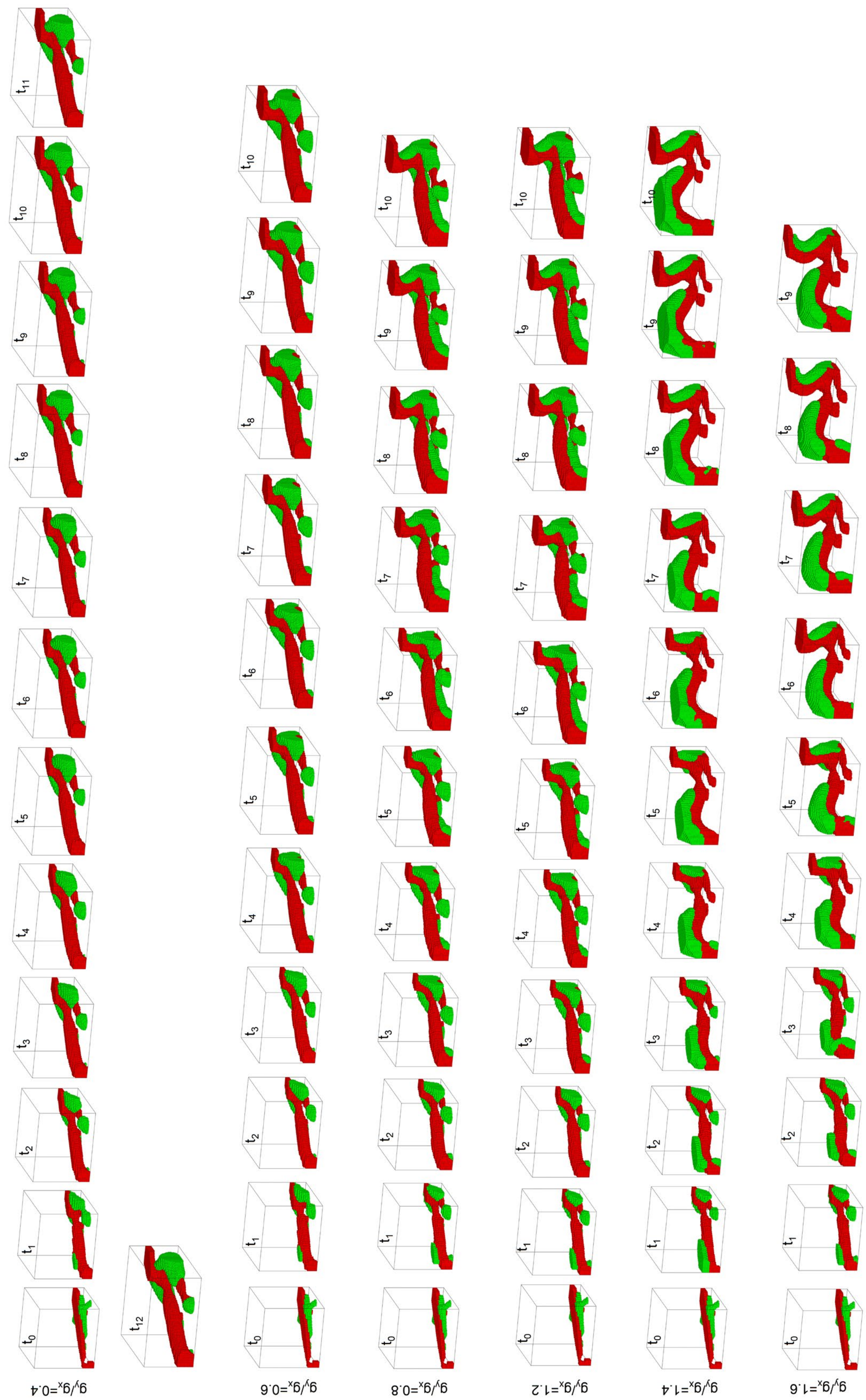

Figure S16. Multi-voxel embryoids with $\gamma_{\mathrm{E}} = 0.098$, and the evolutionary histories for various optimized vertebrates in the allometric growth model.

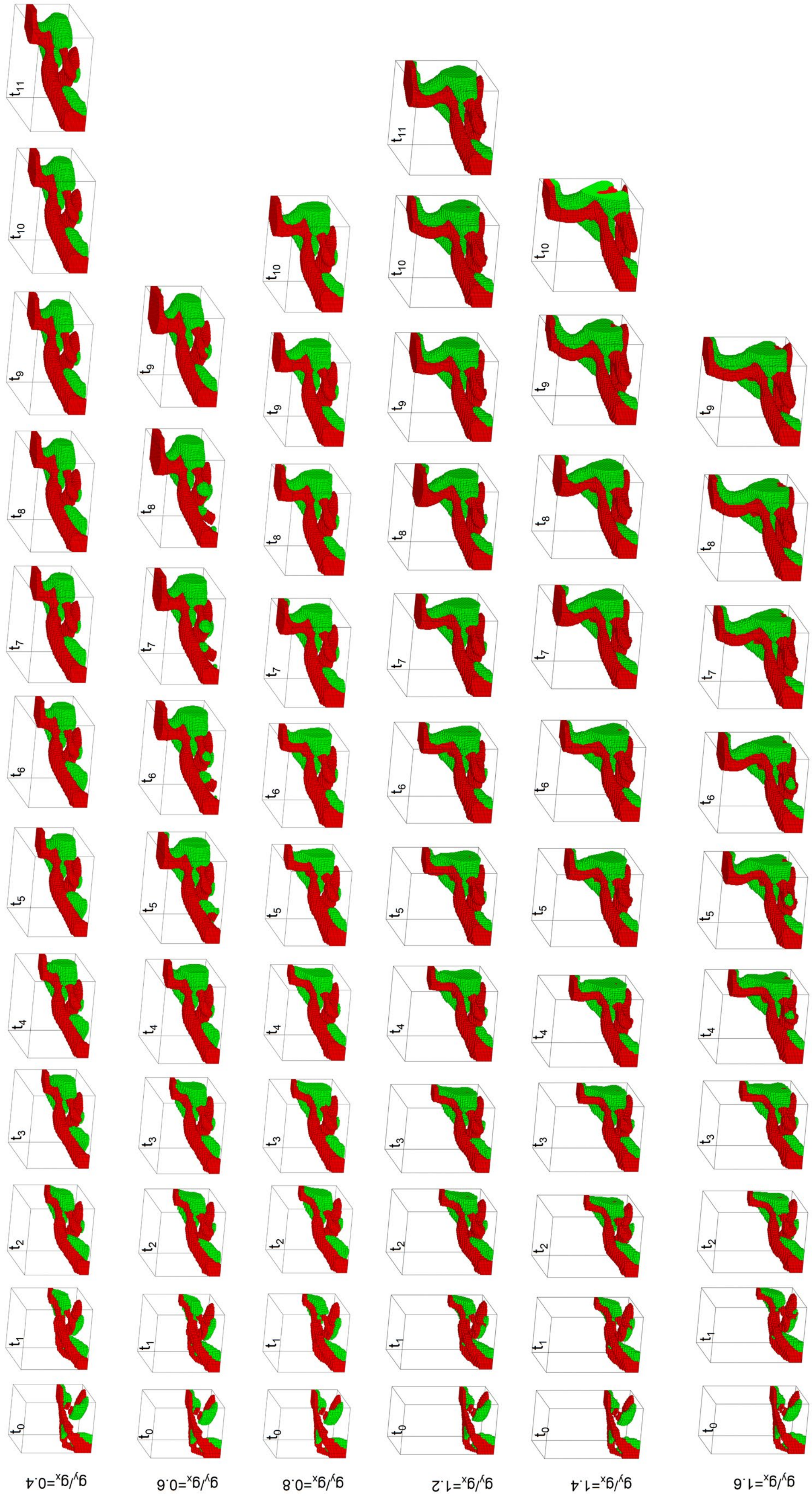

Figure S17. **Multi-voxel embryoids with $\gamma_{\mathrm{E}} = 0.354$, and the evolutionary histories for various optimized**

vertebrates in the allometric growth model.

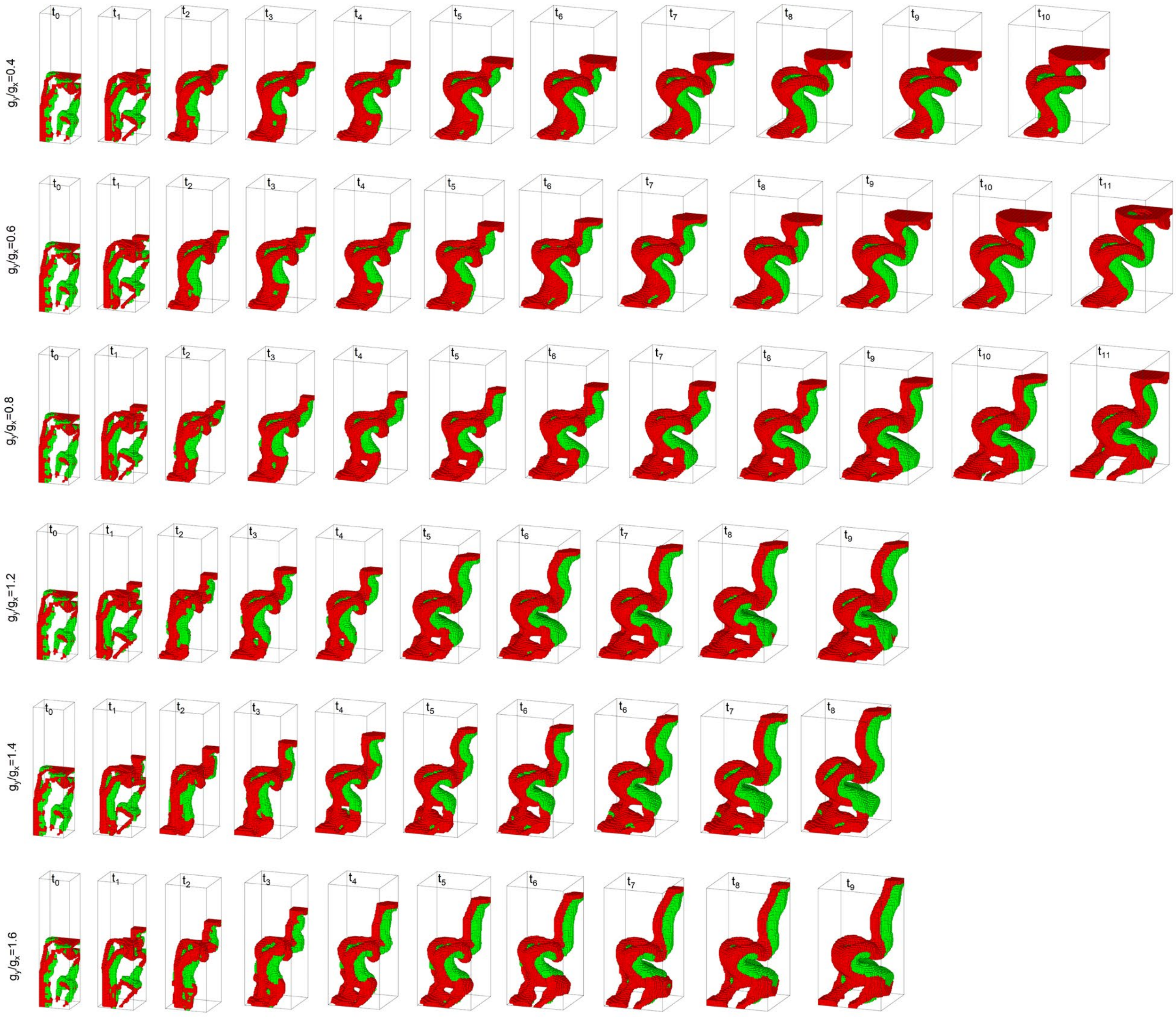

Figure S18. Multi-voxel embryoids with $\gamma_{\mathrm{E}} = 1.141$, and the evolutionary histories for various optimized vertebrates in the allometric growth model.

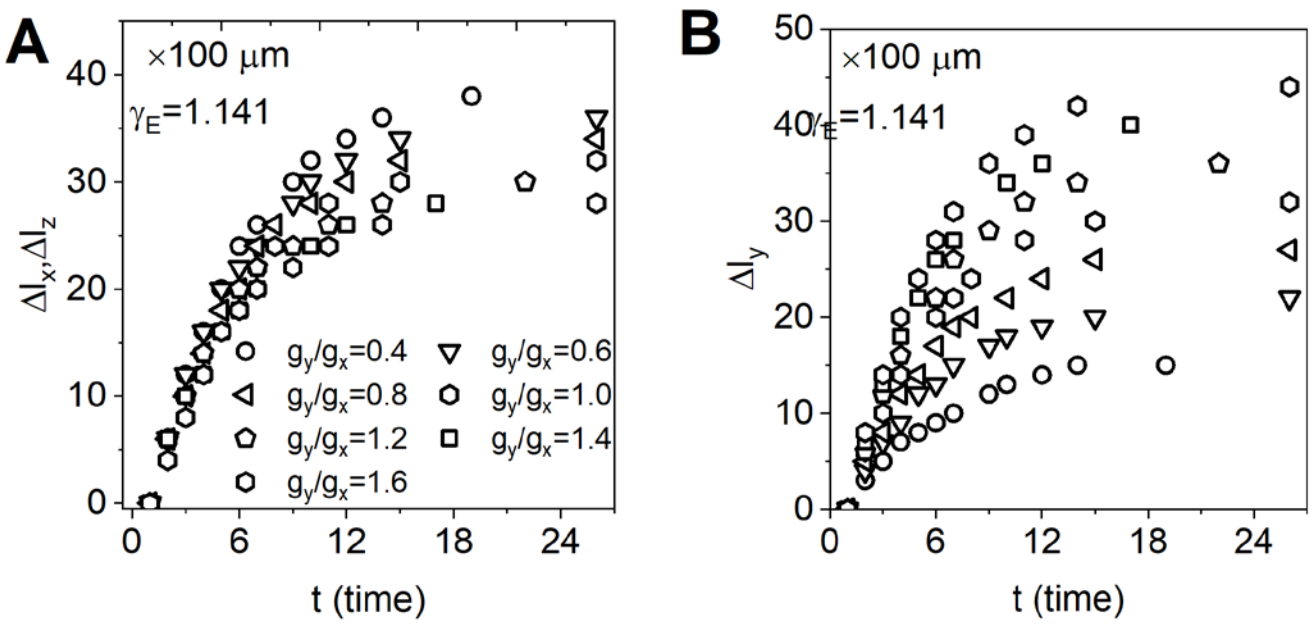

Figure S19. Voxel increments in the *x* or *z*-direction (A) and *y*-direction (B) varying with growth rates.

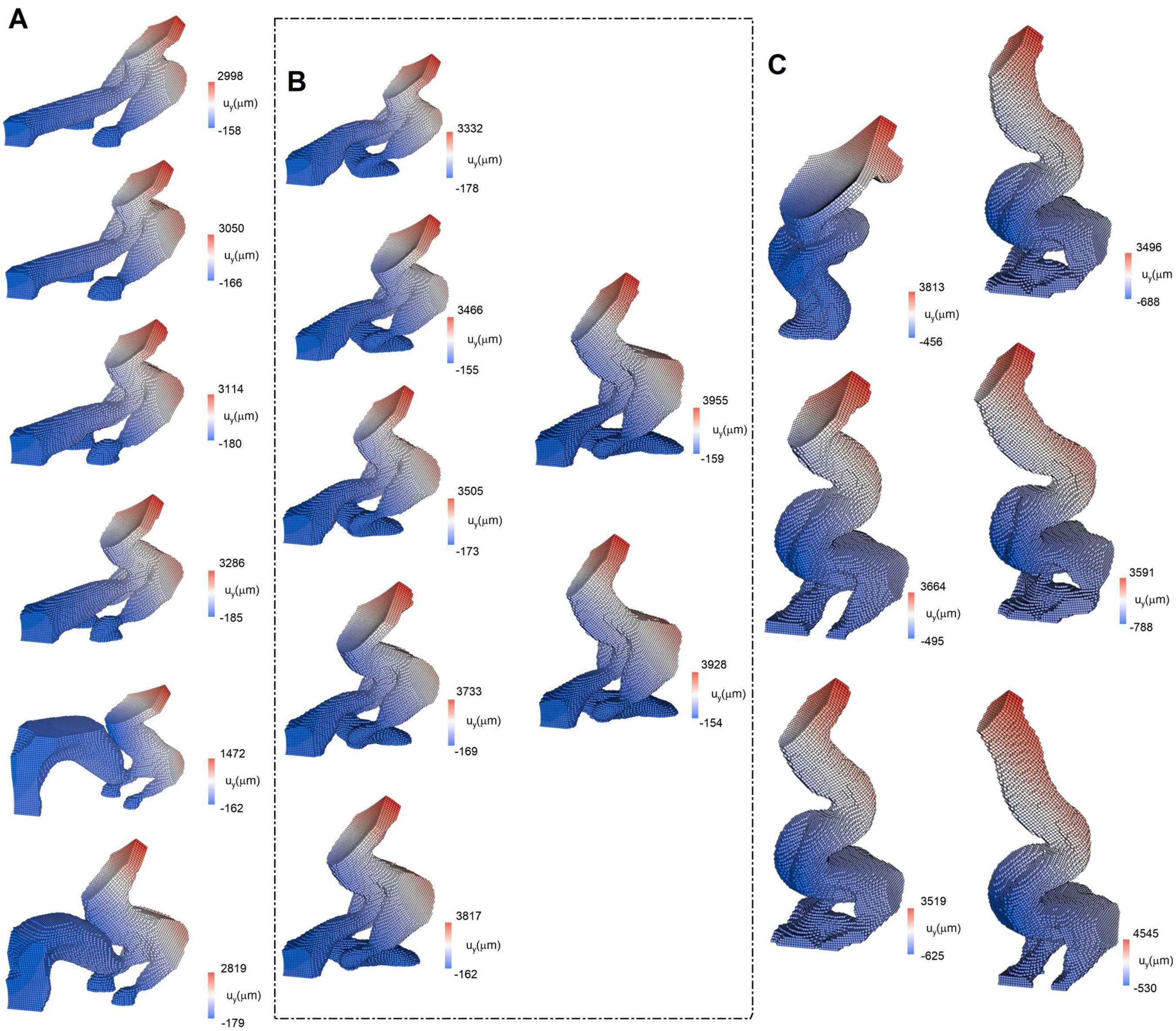

Figure S20. Biomechanical propulsions of the optimized vertebrates in Figure 3.

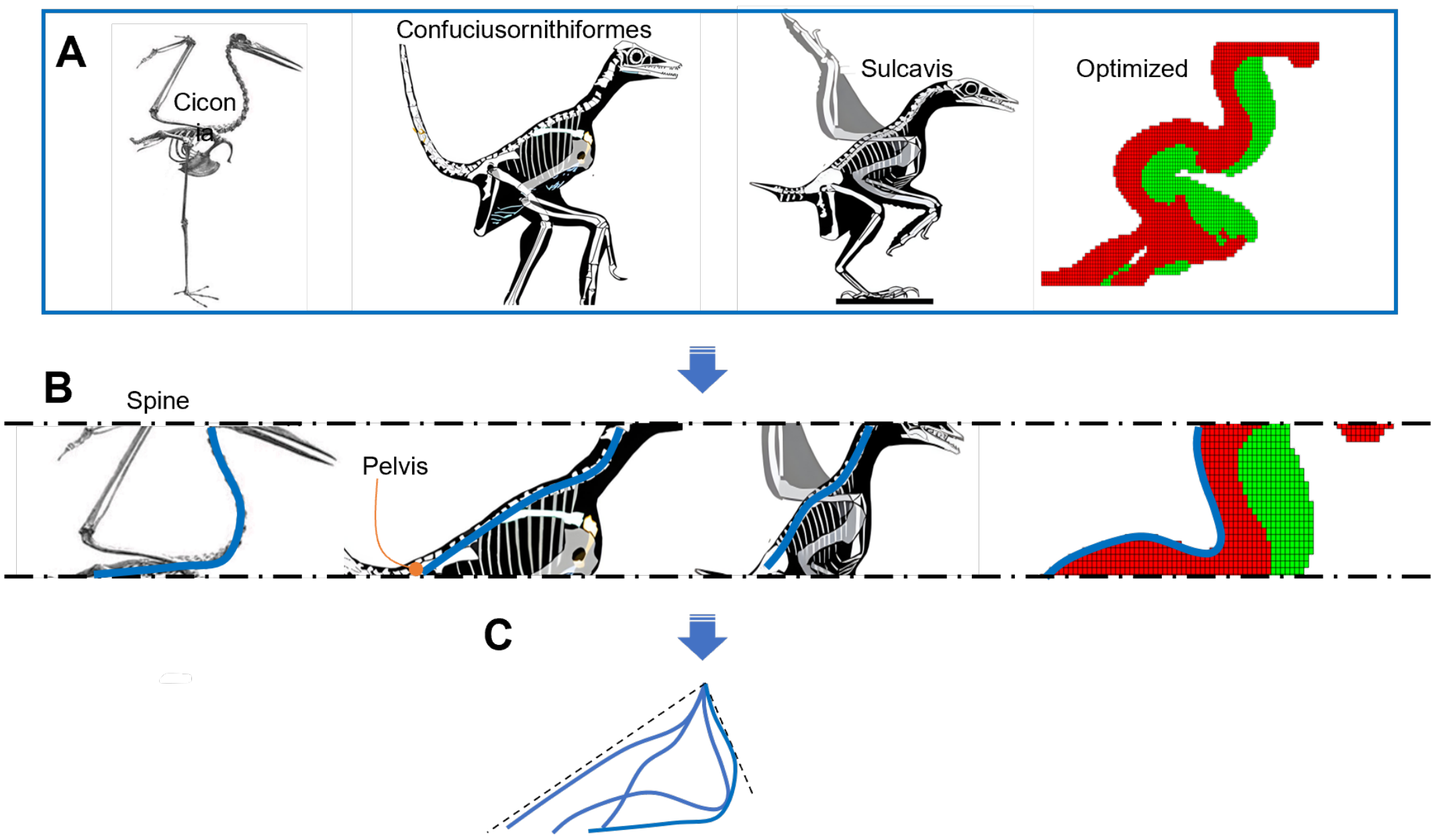

Figure S21. **The method for the extraction of animal vertebral morphology**.

(A) *Cicon* is from https://reptileevolution.com/cariama.htm; *Confunciusornithiformes* is from https://reptileevolution.com/confuciusornis.htm ;

(B) Sulcavis is from https://reptileevolution.com/enantiornithes1.htm. (B, C) Spine samples.

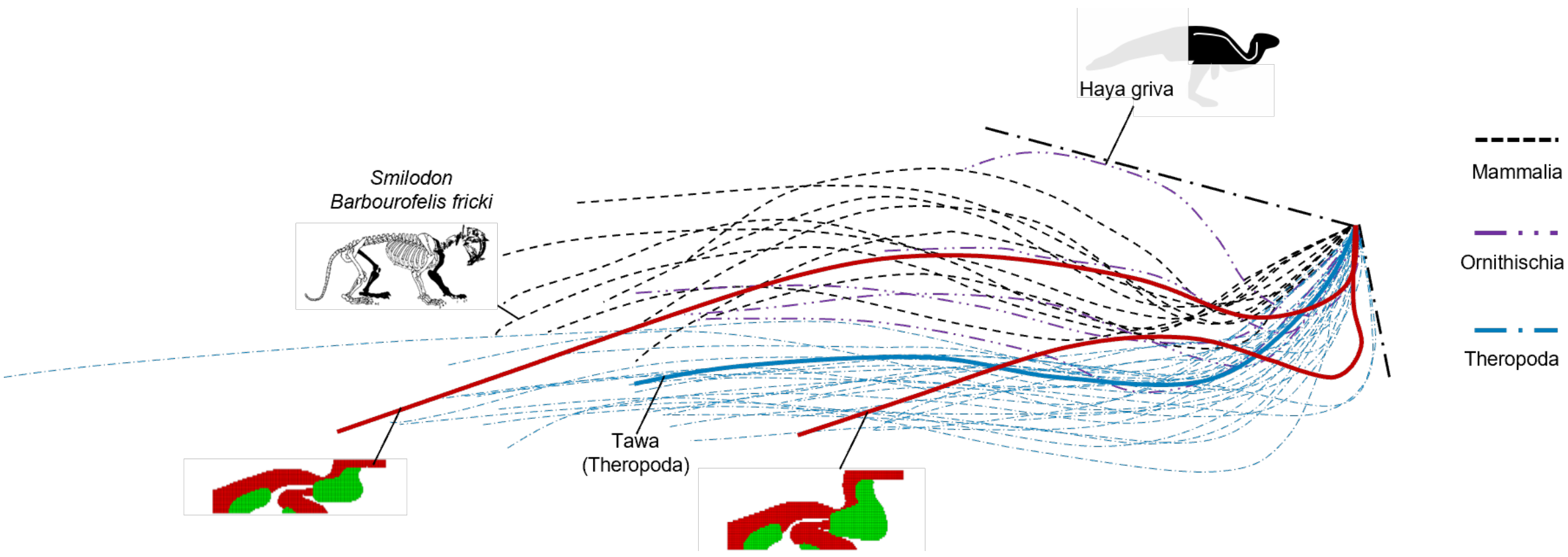

Figure S22. **Spine morphologies of Mammalia, Ornithischia, and Thereopoda**. (A) Barbourofelis fricki is from https://reptileevolution.com/smilodon.htm.

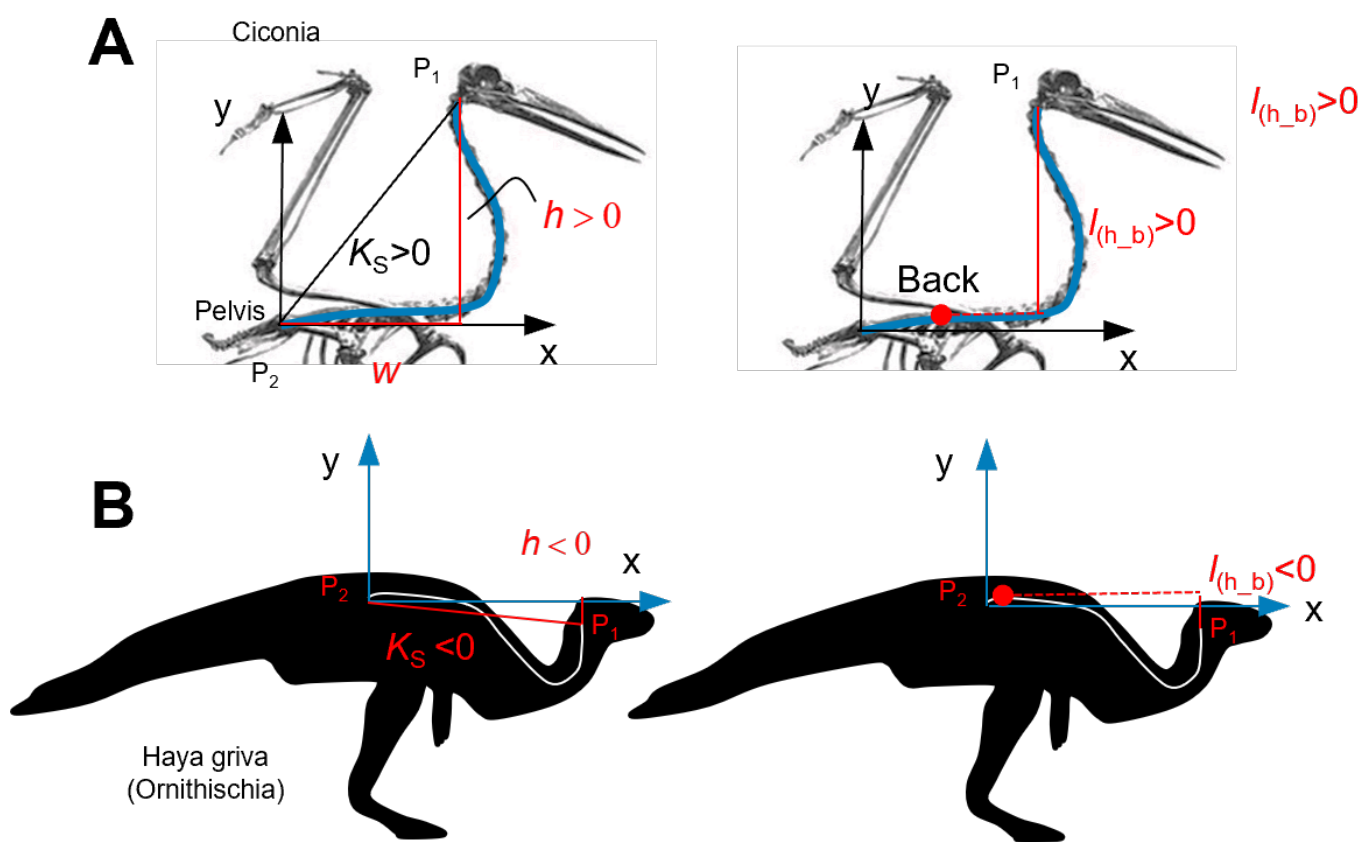

Figure S23. **Two cases of computed spine shape parameters** K_S **and** $\Delta l_{h\_b} = l_{h\_b} / h$. In (A), the height of animal head is larger than its back with $K_S = h / w > 0$, while in (B), the head height is smaller than its back, yielding $K_S < 0$.

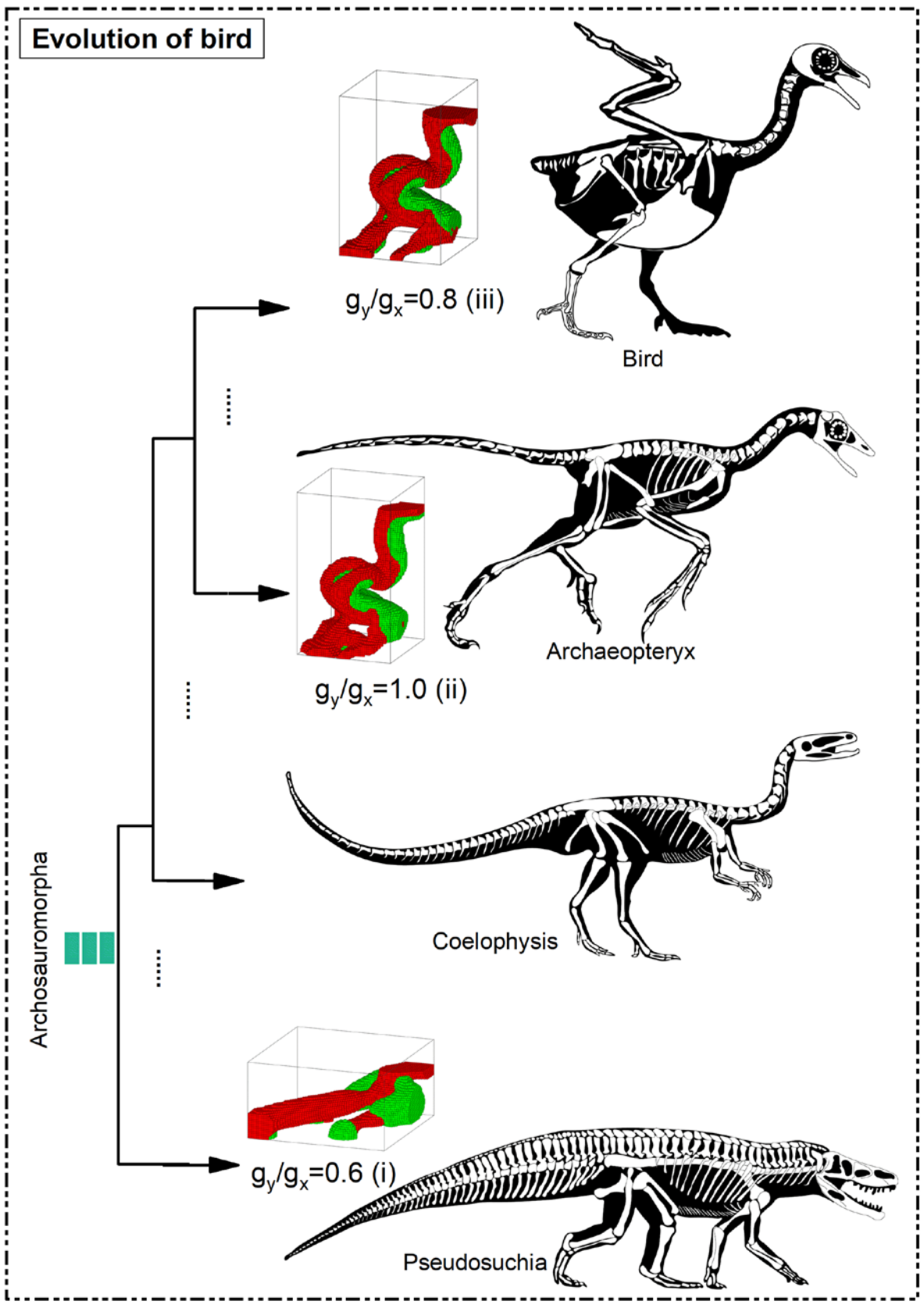

Figure S24. Conceptual diagram illustrating the evolutionary lineage of birds, from bottom to top: *Pseudosuchia*, *Coelophysis*, *Archaeopteryx*, modern birds [2].

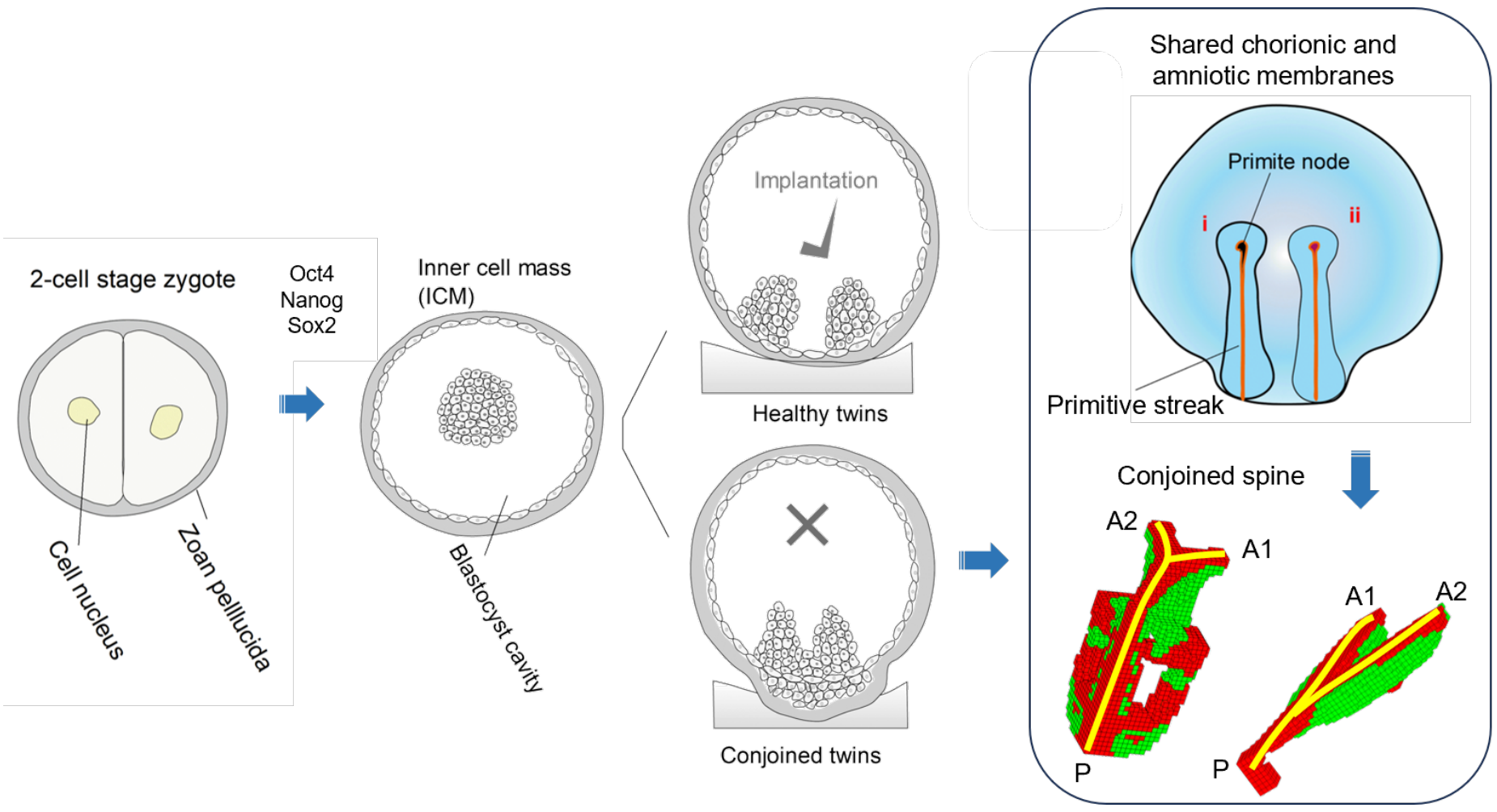

Figure S25. The development of malformation. In the blastula stage of embryo development, if the cell cluster successfully separates into sub-subcellular balls after implantation, twins will form; Conversely, if the separation fails, a malformed fetus will develop. The malformed embryo develops two primitive streaks, which will form a conjoined spine. The optimized digital embryoids can also have different conjoined spines with two heads.

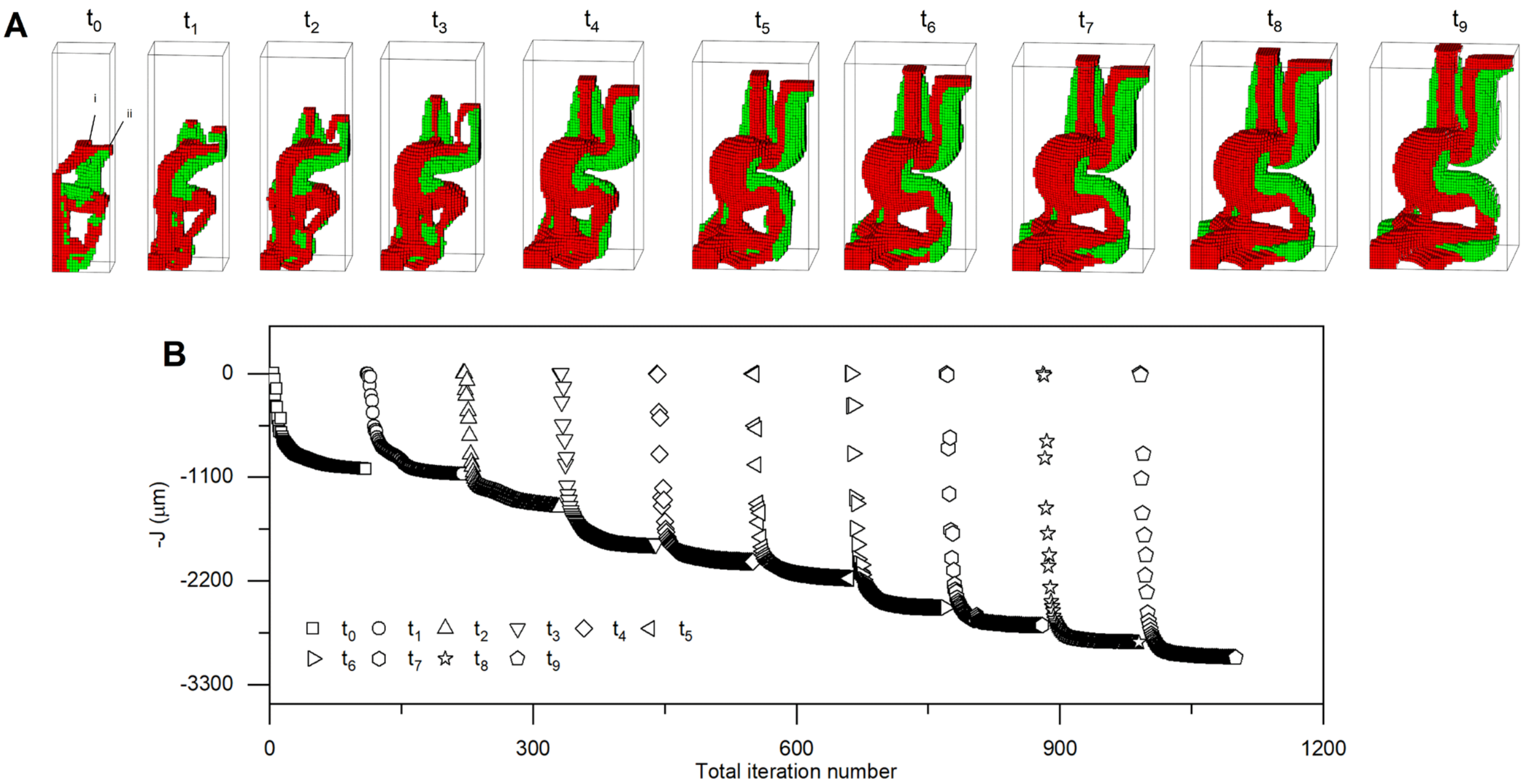

Figure S26. Topological evolution process of the two-head virtual vertebrates. (A) Optimized results. (B) Optimization history.

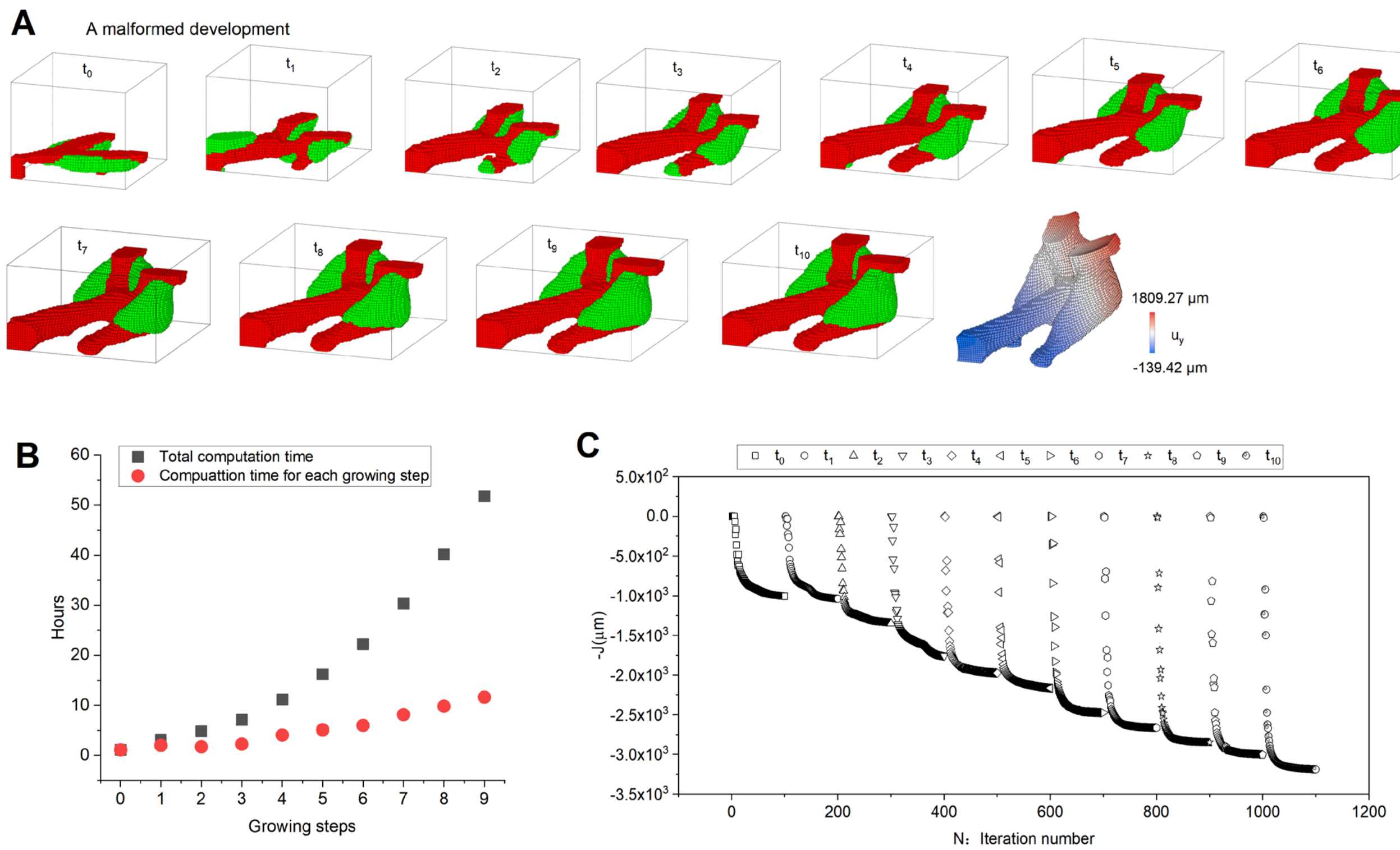

Figure S27. Developmental evolution of digital bicephalic vertebrates. (A) Optimized outcomes and a modern two-head crocodile. (B) Computation time. (C) Evolutionary history of the objective function.

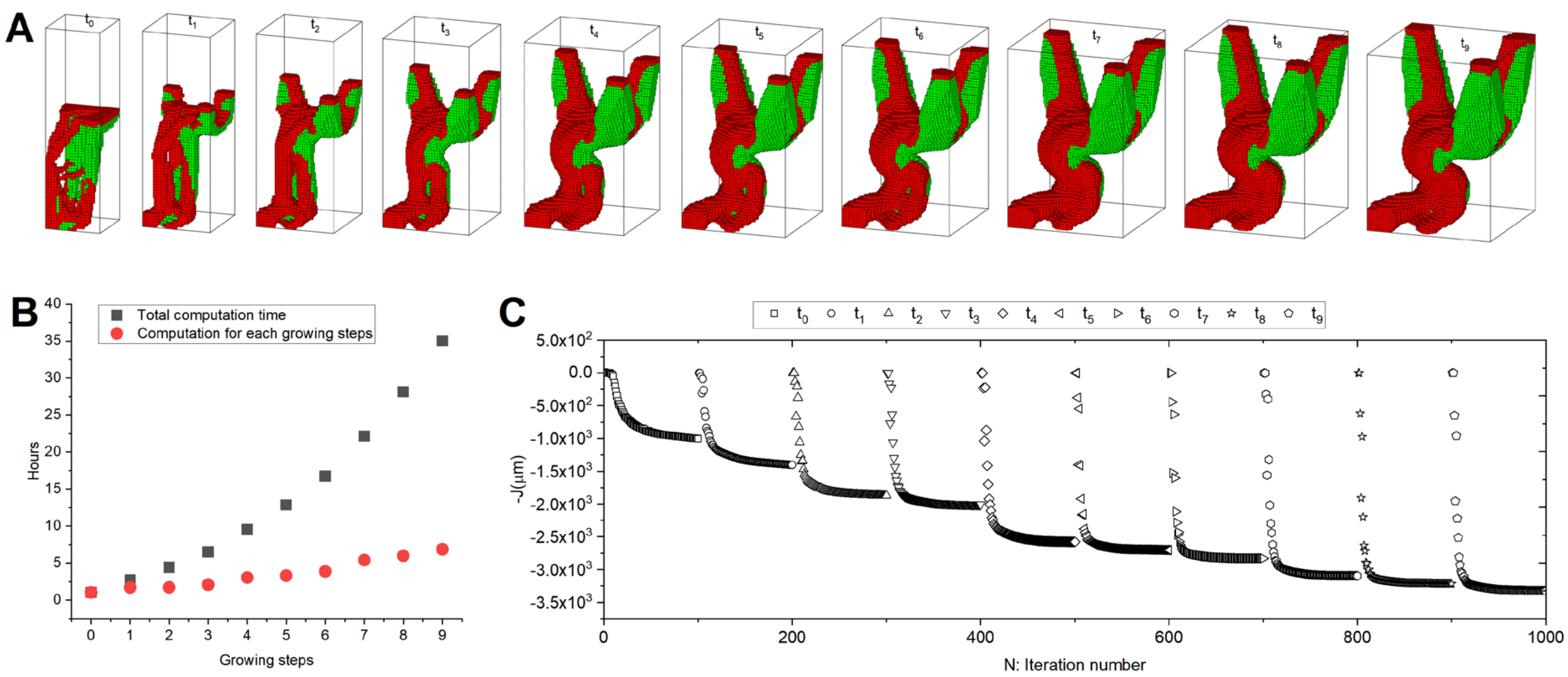

Figure S28. Three-head virtual vertebrates. (A) Optimized results. (B) Computational time. (C) Evolutionary history of the objective function.

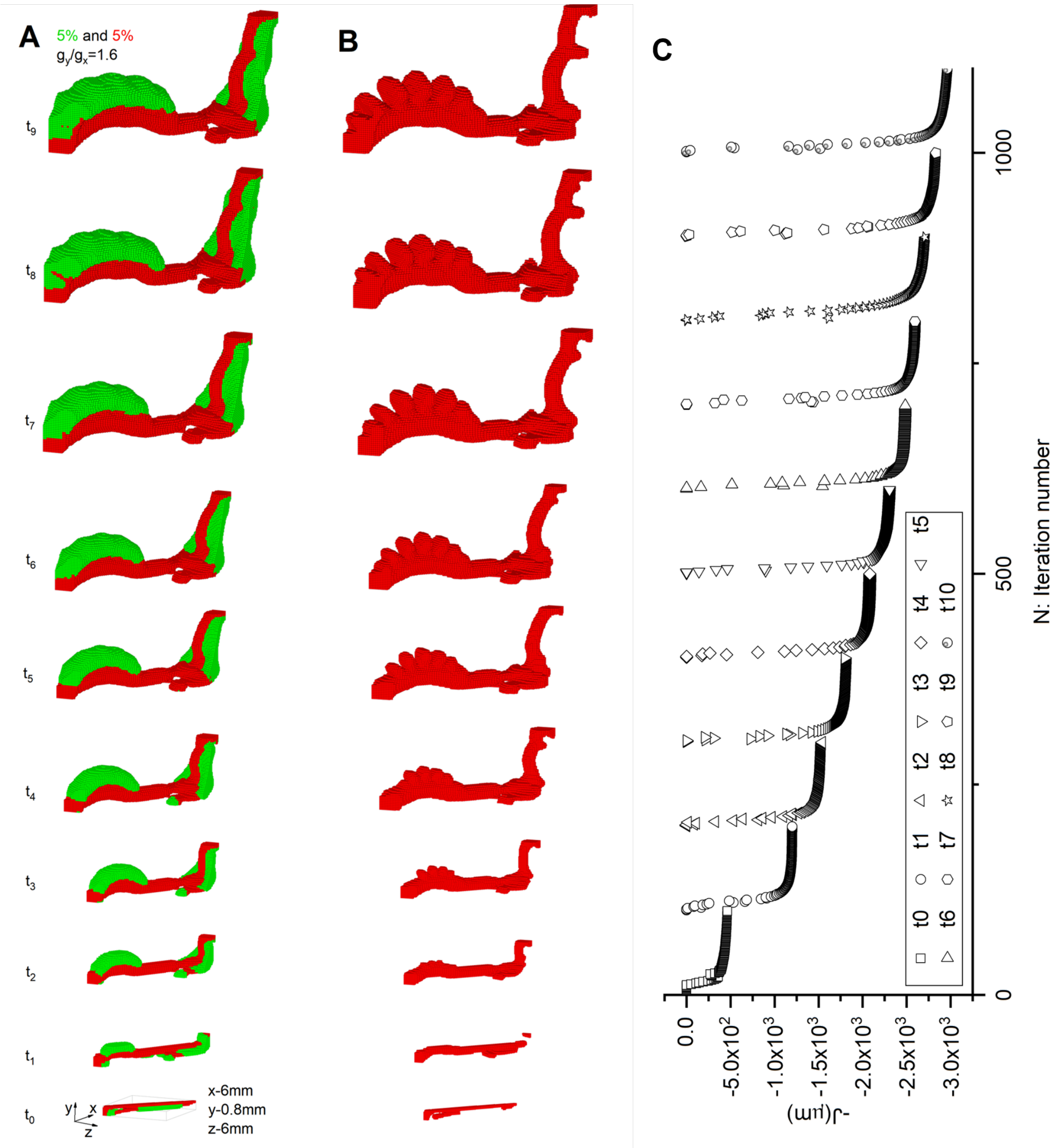

Figure S29. Exotic vertebrates. (A) and (B) Optimization processes of the multi-voxel vertebrate. (C) Optimization history.

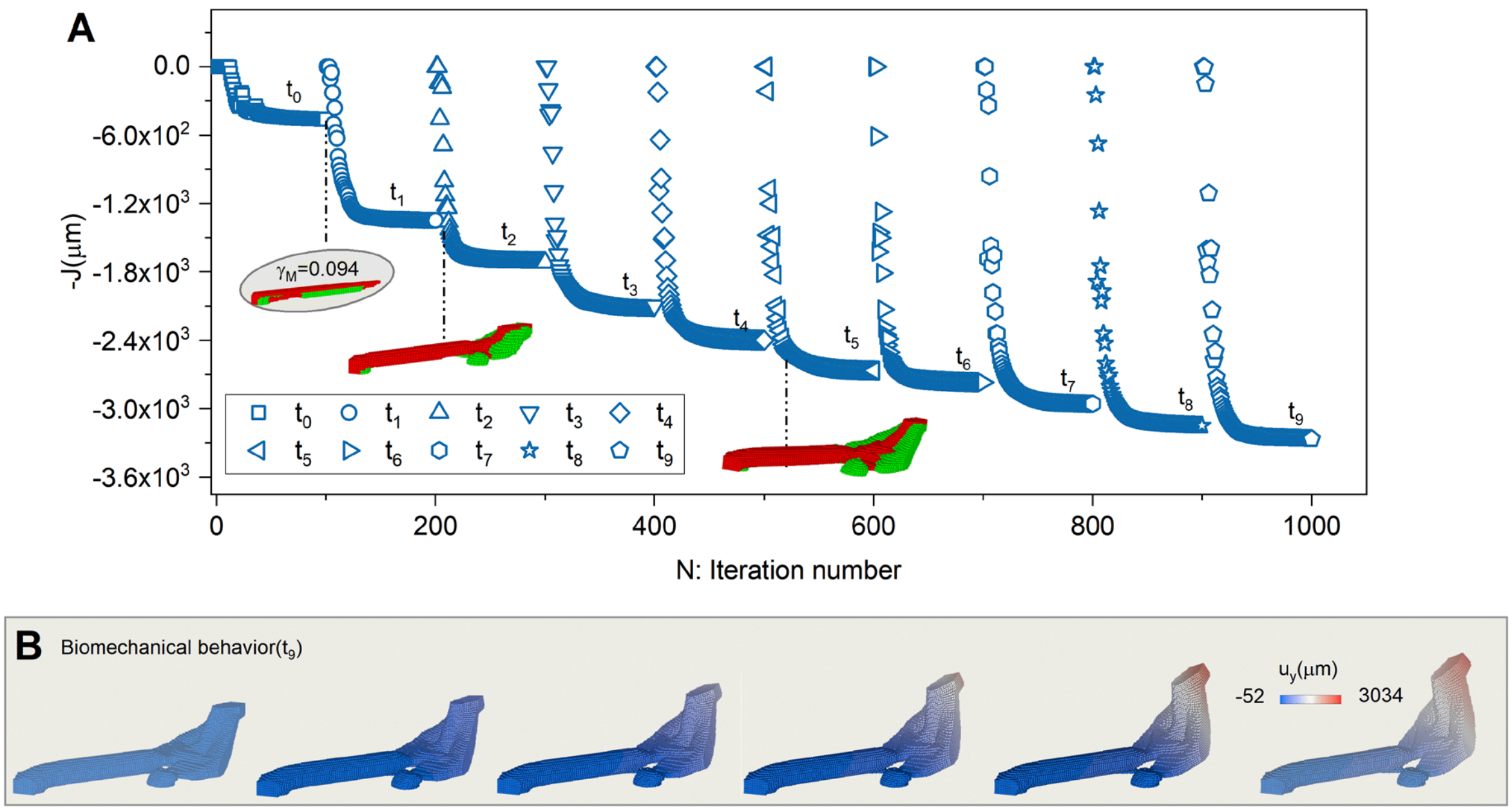

Figure S30. Optimization history. (A) The entire spatiotemporal optimization process. (B) Active behavior exhibited by the optimized virtual vertebrates.

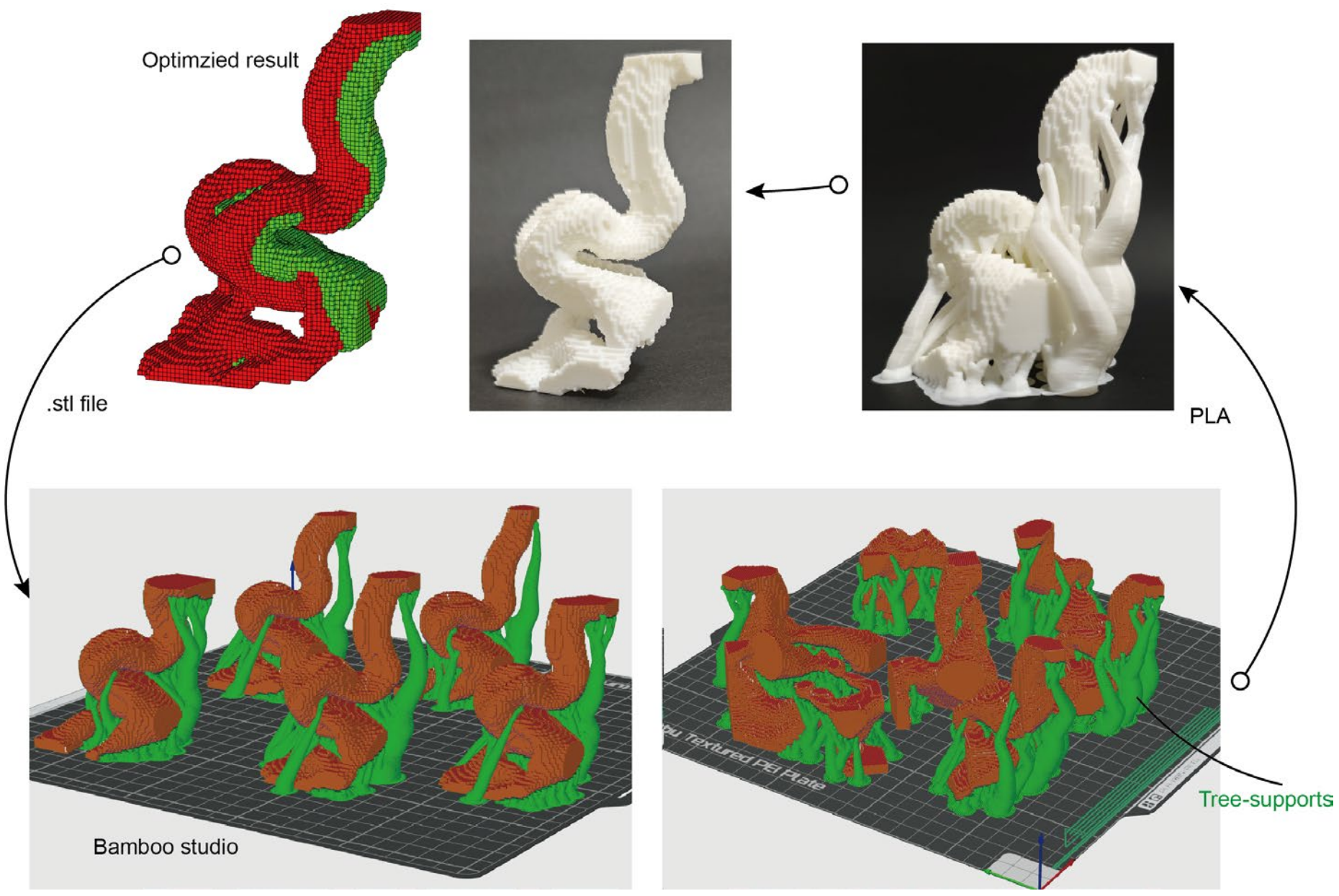

Figure S31. 3D printed animals with PLA materials.

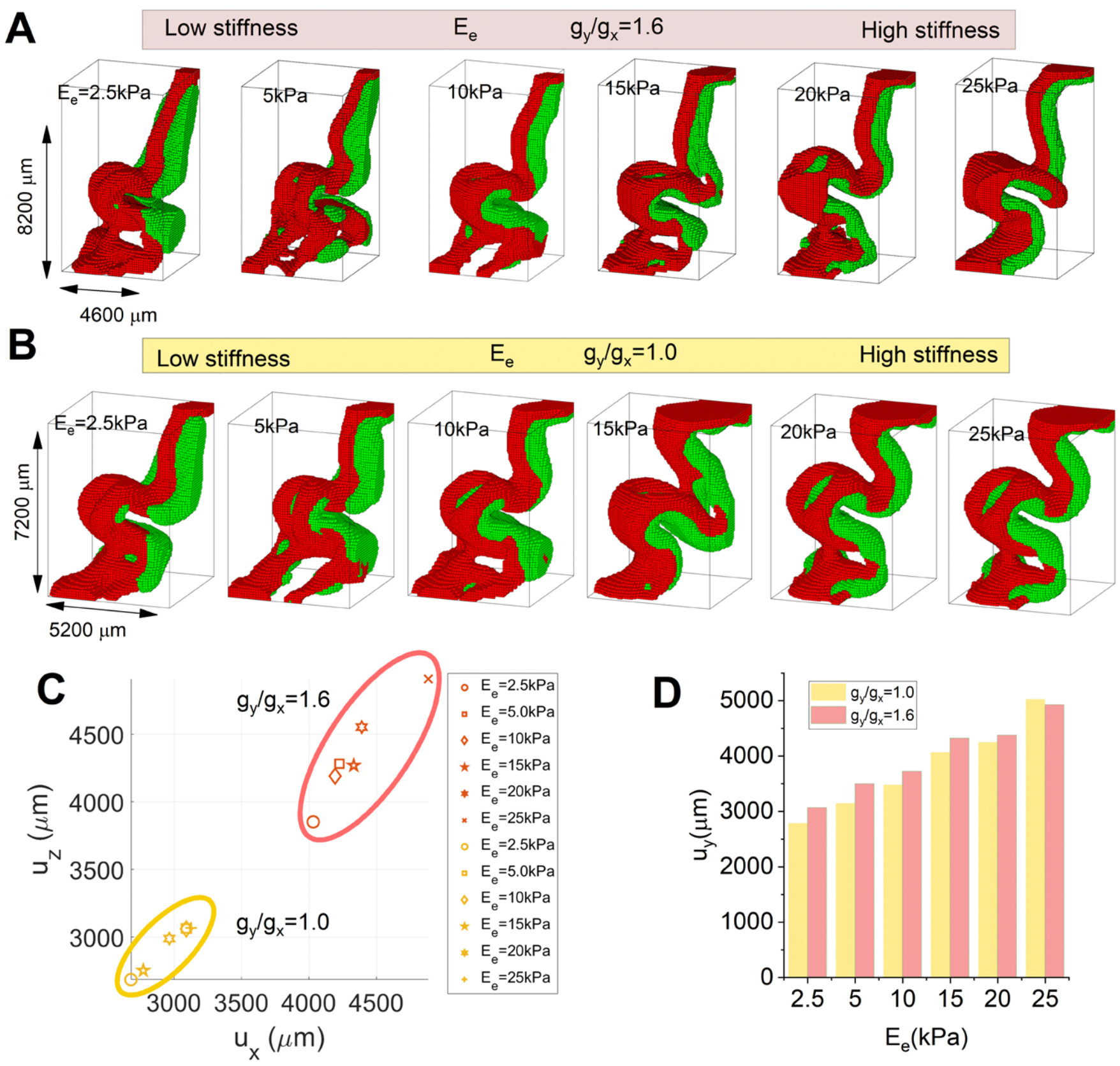

Figure S32. Impact of variations in voxel modulus on the evolution of optimized multi-voxel vertebrates. (A) and (B) Topological changes in body characteristics under allometric growth (upper) and isotropic growth (lower). (C) and (D) Head propulsions.

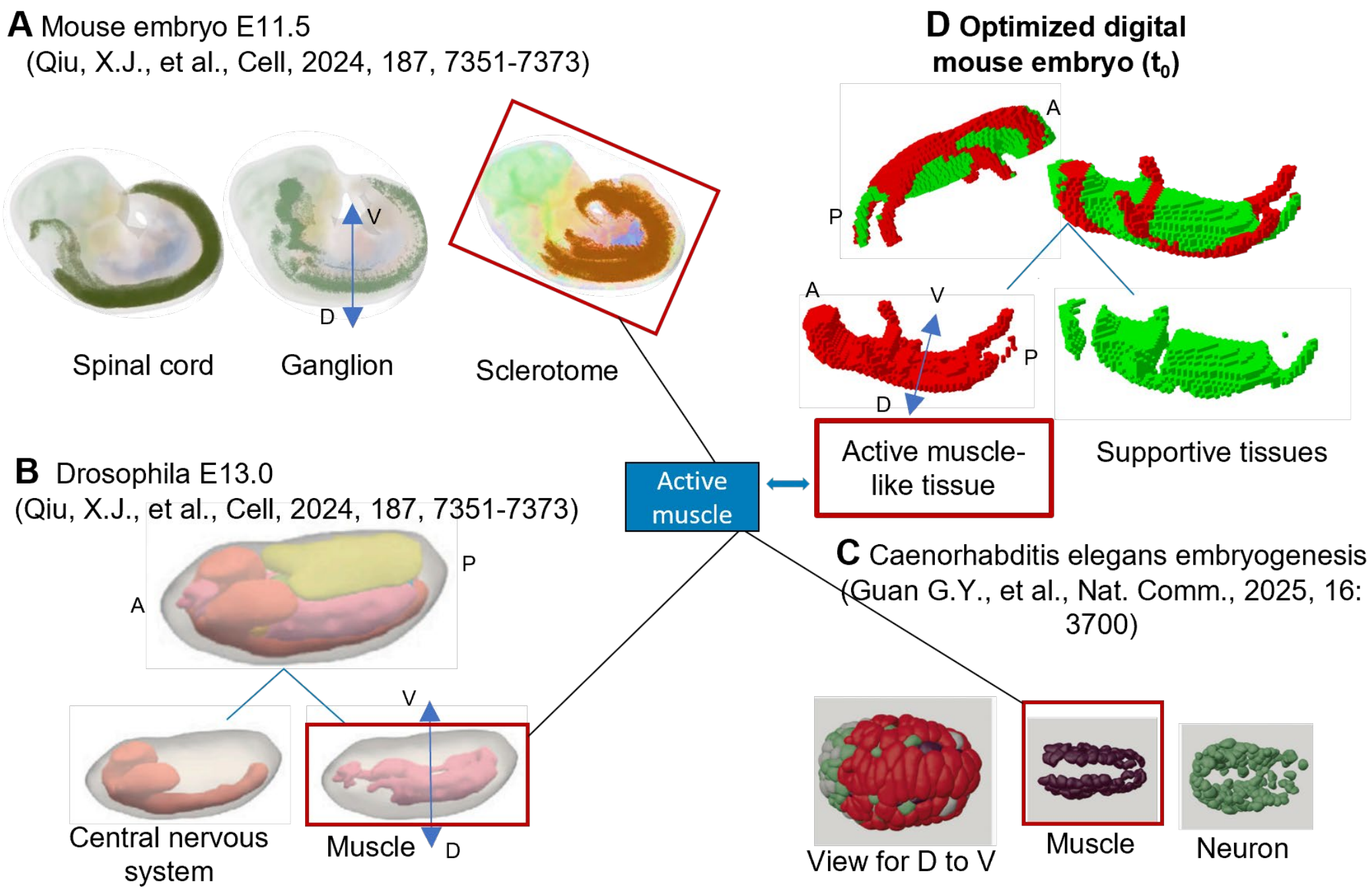

Figure S33. The optimized active red tissues (D) have the similar morphology to the muscle-like tissue in the mouse, drosophila and elegans embryo (A, B, C) [3, 4].

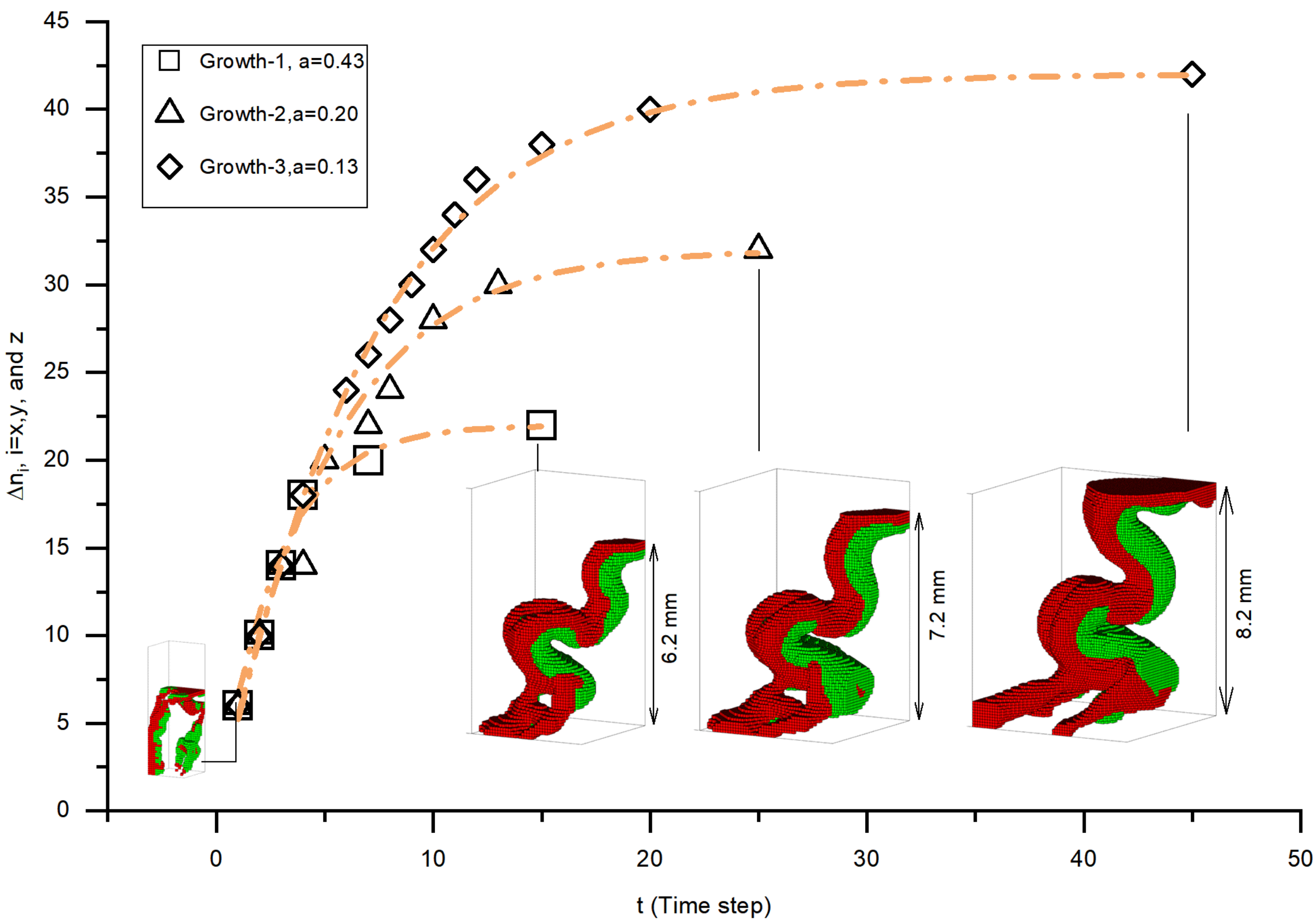

Figure S34. Scaling analysis for the virtual growth with various voxels, with similar body morphologies. Parameter *a* affects the slope of the curves, which is shown in Equation (1). In Figure S36,

the growth velocity is not identical, with distinct body morphologies.

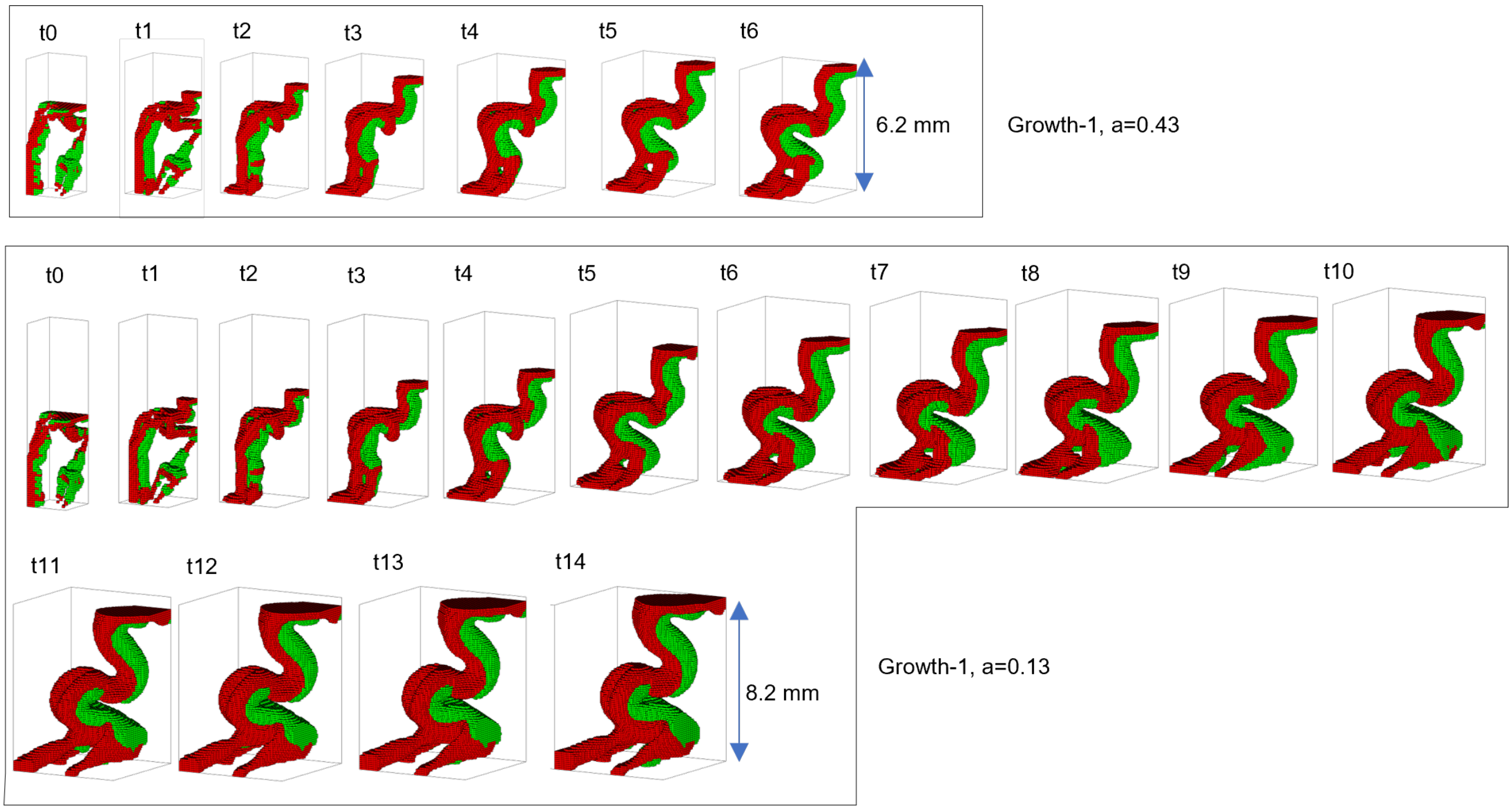

Figure S35. Scaling growth histories in Figure S34.

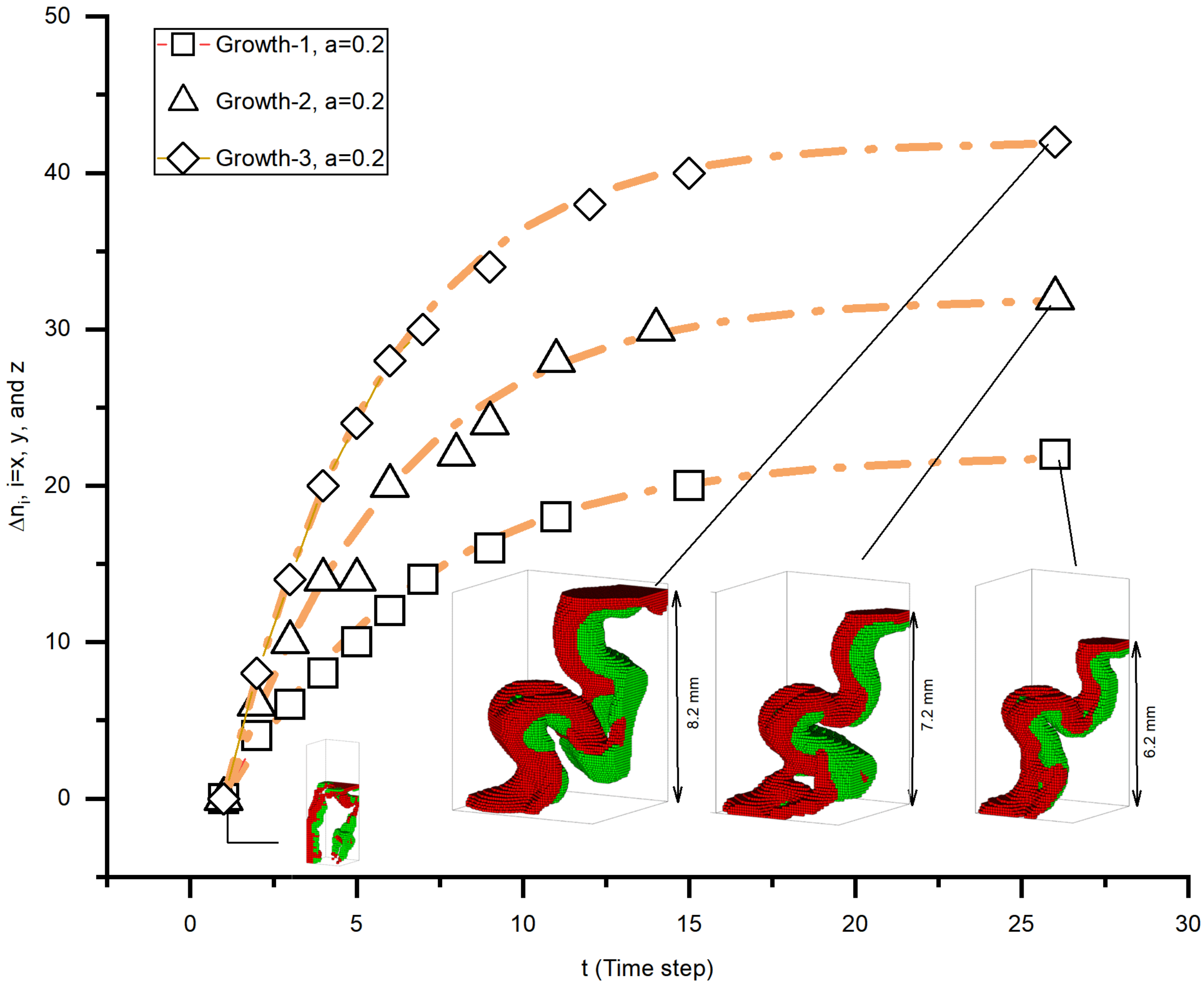

Figure S36. Scaling analysis for the virtual growth with various voxels, with various body morphologies.

Table S1. Statistical analysis of morphological parameters of ancient and modern vertebrates (Figure S2).

	Name	Height	Length	Slope: γ
Group-1	Proterogyrinus	0.227	1	0.227
	Hedgehog	0.268	1	0.268
	Crocodile	0.351	1	0.351
Group-2	Rhinoceros	0.468	1	0.468
	Pseudosuchinas	0.499	1	0.499
	Ankylosaurus	0.502	1	0.502
	Cat	0.568	1	0.568
	Triceratops	0.598	1	0.598
	Horse	0.794	1	0.794
	Dog	1	0.994	1.006036217
Group-3	Tyrannosaurus	1	0.772	1.295336788
	Archaeopteryx	1	0.6.98	1.432664756
	Cock	1	681	1.468428781
	Kangaroo	1	0.661	1.512859304
	Velociraptor	1	0.648	1.543209877
	Dryopithecus	1	0.56	1.785714286
	Brachiosaurus	1	0.443	2.257336343
	Ramapithecus	1	0.312	3.205128205
Group-4	Early homo	1	0.121	8.26446281
	Penguin	1	0.085	11.76470588

Table S2. The number of 3D voxels incorporated in the design space for multi-voxel vertebrates, spanning from the embryonic stage to the mature stage (Figure 2A), and each voxel size is $l_e^3 = 100^3\ \mu\mathrm{m}^3$.

Embryoids

Case	1	2	3	4	5	6	7	8	9
n_x	48	44	40	36	32	28	24	20	16
n_z	48	44	40	36	32	28	24	20	16
n_y	7	8	10	12	16	20	28	40	63
n_{T}	16128	15488	16000	15552	16384	15680	16128	16000	16128

Mature individuals

	#1	2	3	4	5	6	7	8	9
n_x	76	72	70	68	64	62	58	52	46
n_z	76	72	70	68	64	62	58	52	46
n_y	34	37	40	43	48	53	61	72	93
n_{T}	196384	191808	196000	198832	196608	203372	205204	194688	196788

Table S3. Heights along the y-direction of optimized multi-voxel vertebrates (Figure 3).

g_y / g_x	Case2	Case5	Case8
	l_y : ×100μm		
0.4	25	34	55
0.6	29	39	62
0.8	33	43	67
1.0	36	48	72
1.2	39	51	76
1.4	41	55	80
1.6	43	58	82

Table S4. Animals in Figure 4.

https://reptileevolution.com/	
Figure 4A (62 animals)	Figure 4B (14 animals)<br>Breviparopus, Sauroposeidon, Sonorasaurus, Cedarosaurus, Pleurocoelus, Pelorosaurus, Archbishop, Brachiosaurus, Lusotitan, Bothriospondylus, Daanosaurus, Lapparentosaurus, Volkheimeria, Europasaurus
Aves/Birds	Figure S22
Birds (Archaeopteryx, Sapeornis, Scansoriopterygidae 1, Scansoriopterygidae 2,<br>Zhongornis-Confuciusornis, Enantiornithes 1, Archaeornithura, Hesperornis, Eogranivora, Yanornis-Ichthyornis, Gansus-Hongshanornis, Iteravis)	Theropoda (Tawa, Marasuchus, Procompsognathus, Sinocalliopteryx, Dracoraptor, Coelophysis, Scipionyx, Dilong, Guanlong, Deinocheirus, Struthiomimus,<br>Tianyuraptor, Zhenyuanlong, Gorgosaurus, Juravenator, Sinosauropteryx, Falcarius, Limusaurus, Khaan, Ornitholestes, Saurornitholestes, Microraptor, Sinornithosaurus, Velociraptor, Haplocheirus, Shuvuuia, Sinornithoides)<br>Mammalia (Cynogale bennettii, Ailurus fulgens Bsmilodon-arbourofelis frick, Speothosveanticus Canis:Homotherium latidens, Protictitherium Eomaia scansorial, Didelphis. Vincelestes Paroodectes, Talpa europaea)<br>Ornithschia (Chilesaurus, Haya griva Heterodontosaurus tucki, Stegoceras validum Lesothosaurus diagnosticus)
Euornithes (Apteryx-Pseudocrypturus, Rhea-Casuarius, Struthio-alaelodus)	
Neognatha (Megapodius, Vultur)	
Falconiformes (Sagittarius-Terror Birds, Pandion, Falco-Didunculus, Tyto-Apus)	
Cuculiformes (Cariama-Phoenicopterus, Geococcyx, Oedicnemus, Vegavis, Pseudocrypturus, Apteryx, Rhynchotus, Changzuionis, Yanornis, Sagittarius, Ciconia, Fulica, Psophia, Corvus)	
Galliformes (Columba, Goura, Raphus, Gallus, Passer, Opisthocomus, Aptornis, Dinornis Ara-Gastornis)	
Corvidae (Corvus-yanocitta)	
Ralliformes (Crex, Charadrius, Rallus)	
Gruiformes (Grus-urypyga, Chroicocephalus, Himantopus, Archilochus)	
Coraciformes (Thalasseus, Uria-ptenodytes, Megaceryle-abiru, Ardea-iconia, Gavia)	
Scopidae (Scopus, Pteroglossus-Buceros, Cyrilavis, Urocolius)	
Anseriformes (Platalea, Presbyornis-Anas)	
Suliformes (Macronectes-Pelagornis, Fratercula-Pinguinus)	

Table S5. Voxels for the growth design domains of mouse (Figure 6).

	t_0	t_1	t_2	t_3	t_4	t_5	t_6
n_x	60	62	66	68	72	74	76
n_z	60	62	66	68	72	74	76
n_y	18	20	24	26	30	32	34

References:

1. Ksepka, D. T. et al. Largest-known fossil penguin provides insight into the early evolution of sphenisciform body size and flipper anatomy. Journal of Paleontology 97, 434-453, (2023).
2. Xu, X. et al. An integrative approach to understanding bird origins. Science 346, 1253293, (2014).
3. Qiu, X. et al. Spatiotemporal modeling of molecular holograms. Cell 187, 7351-7373. e7361, (2024).
4. Guan, G. Y., et al. Cell lineage-resolved embryonic morphological map reveals signaling associated with cell fate and size asymmetry. Nature Communications 16.1 3700, (2025).
